# GSNO-functionalized hydroxyapatite nanofertilizer for prolonged delivery, detoxification, and yield improvement in maize

**DOI:** 10.1038/s41467-026-77610-0

**Published:** 2026-09-16

**Authors:** Da-Sol Lee, Ho Young Yoon, Ashim Kumar Das, Jun-Seo Kang, Bong-Gyu Mun, Adil Hussain, In-Jung Lee, Jong-Rok Jeon, Byung-Wook Yun

**Affiliations:** 1 Department of Applied Biosciences, Kyungpook National University, Daegu, South Korea; 2 Laboratory of Natural Materials Technology, Åbo Akademi University, Turku, Finland; 3 Department of Applied Life Chemistry, Gyeongsang National University, Jinju, South Korea; 4 Institute of Agriculture & Life Science (IALS), Gyeongsang National University, Jinju, South Korea; 5 Department of Environmental and Biological Chemistry, Chungbuk National University, Cheongju, South Korea; 6 Department of Agriculture, Abdul Wali Khan University Mardan, Mardan, Khyber Pakhtunkhwa, Pakistan

**Keywords:** Plant stress responses, Nanoparticles, Plant biotechnology, Nanobiotechnology

## Abstract

The agricultural use of nitric oxide (NO) is restricted by the instability of conventional donors. Here, we develop a facile one-pot method to immobilize S-nitrosoglutathione (GSNO) onto humic acid-coated hydroxyapatite nanoparticles (GSNO-HAs-Fe-HAP NPs). Particle characterization confirms favorable properties for agricultural applications, including prolonged NO release (>6 days) and the mitigation of maize cadmium (Cd) stress. Release test verifies the corelease of humic acid and crop nutrients, while confocal microscopy demonstrates robust NP uptake in maize. Under Cd stress, GSNO-HAs-Fe-HAP outperforms commercial NPK fertilizer, improving photosynthetic efficiency and agronomic traits, reducing grain Cd accumulation by 86%, and upregulating phytochelatins, S-nitrosothiols, and metal transporters (*ZmPCR, ZmABCC1, and ZmABCG40*). The NP composite reduces ROS accumulation and enhances antioxidant enzyme activity, osmolytes, phenolics, flavonoids, and stress phytohormones. Furthermore, NPs significantly improve soil health parameters and the rhizosphere microbiome. Thus, GSNO-HAs-Fe-HAP is a promising nanofertilizer for prolonged NO delivery and Cd stress mitigation.

## Abstract (web summary)

Unstable nitric oxide donors limit agricultural uses. Here, the authors report on the immobilization of S-nitrosoglutathione onto humic acid-coated hydroxyapatite nanoparticles which reduces ROS levels, enhances antioxidant enzyme activity, and secondary metabolic synthesis while improving soil health.

## Introduction

Over the past few decades, nitric oxide (NO), a gaseous signaling molecule, has emerged as a key regulator in plant molecular biology, playing diverse roles in plant physiology^1,2^. NO acts both intra- and intercellularly, exerting positive or negative effects on physiological processes leading to changes in plant growth and development. Despite its promising applications in agriculture, the practical use of NO is constrained by the instability and short lifespan of its donor compounds. These compounds decompose rapidly under various environmental conditions, including elevated temperatures (e.g., 37 °C), light exposure, and the presence of catalytic metal ions, leading to the formation of NO, nitrite (NO_2_^−^), and oxidized glutathione (GSSG)^3^. Therefore, there is an urgent need for strategies that enhance the stability and sustained release of NO for agricultural applications.

To address this issue, various NO immobilization techniques have been developed. For instance, Yang, et al.^4^ demonstrated that a layer-by-layer assembly of sodium alginate and organoselenium-modified polyethyleneimine enables controlled, time-dependent release of S-nitrosothiols. Similarly, biodegradable polymersomes embedded within three-dimensional hydrogels have been shown to provide sustained NO release^5^. In agricultural settings, NO-encapsulated nanoparticles (NPs) have been applied to crops, such as maize^6,7^, cherry^8^, sugarcane^9^, and soybean^10^, offering protection against various environmental stresses.

However, most existing NO delivery systems focus solely on NO release and often rely on external reducing agents, such as reduced glutathione (GSH), to trigger NO release^5^. Moreover, many systems release most of their NO within 24 h, which is suboptimal for agricultural use^6,7^. These limitations underscore the need for NO delivery systems that offer prolonged release with minimal requirement for external triggers. We hypothesized that functionalizing nutrient-rich ceramic particles with both NO donors and intrinsic reducing agents could enable sustained NO release while simultaneously delivering essential nutrients to crops.

Hydroxyapatite (HAP), a calcium phosphate-based ceramic with a hexagonal crystal structure, is increasingly recognized for its potential in next-generation fertilizers^11^. HAP surface modifications can be broadly categorized into two approaches: (1) exploiting non-covalent interactions between surface-exposed calcium ions and oxygen-containing functional groups to induce mild surface acidity and control the slow release of nutrients like calcium and phosphorus^11,12^; and (2) coating HAP with metal–phenolic networks (MPNs), which are stable under alkaline conditions but disassemble in the presence of organic acids, particularly those found in root exudates, thus enabling time-sensitive nutrient and stimulant delivery^13^. Given that most NO donors, such as S-nitroso glutathione (GSNO), S-nitrosocysteine (CysNO), and S-nitroso-N-acetylpenicillamine (SNAP), contain electron-donating groups, they can potentially form organic–inorganic hybrid supramolecular assemblies with metal ions. These assemblies can also incorporate intrinsic reducing agents, contributing to prolonged NO release. Therefore, HAP presents a promising platform for the co-immobilization of NO donors and crop nutrients, responsive to soil microclimate and root exudates.

The addition of humic acids (HAs) further enhances this system. Due to their widespread oxygen-based functional groups (e.g., phenol-quinone cycling), HAs can act as mild reducing agents to trigger NO release from S-nitrosothiols^14,15^. Additionally, HAs are known for their beneficial effects on plant growth, including improving soil aggregation, increasing stress resistance, and promoting phosphorus solubilization^11,16^. In this study, we report the development of a multifunctional, soil-friendly, and root-exudate-responsive nanoparticle composite by engineering hydroxyapatite (HAP), nitric oxide (NO) donor GSNO, humic acid (HAs), and iron (Fe), resulting in GSNO-HAs-Fe-HAP, NPs designed to release plant nutrients and NO simultaneously. Additionally, to evaluate the practical applicability of the composite, maize (*Zea mays*) was selected as a model crop due to its global agricultural significance^17^.

The direct discharge of untreated industrial effluents into water bodies, often used for crop irrigation, poses a serious threat to agriculture^18^. Moreover, both phosphate fertilizers and pollutants from synthetic fertilizers significantly alter soil chemistry, thereby increasing the bioavailability of heavy metals, such as cadmium (Cd)^19^. Cd exposure in maize can severely hinder growth and result in increased Cd accumulation in edible tissues^20,21^. Even at low concentrations, cadmium (Cd) induces oxidative stress, disrupts cellular functions, and can lead to programmed cell death^22^. It also impairs hormonal balance, particularly in the auxin and cytokinin pathways^23^, and interferes with the uptake of essential nutrients, such as iron, magnesium, and calcium^24^. These challenges are further compounded by the overuse of chemical fertilizers to meet global food demand. This has further led to nutrient leaching, environmental pollution, and soil degradation^25^. Together, these factors necessitate the broader context of nutrient management and environmental sustainability that can address heavy metal toxicity while supporting long-term soil and crop productivity for climate-resilient agriculture.

In this context, the GSNO-HAs-Fe-HAP NPs present a promising dual-function solution, mitigating heavy metal toxicity while enhancing nutrient use efficiency. This study invovles the synthesis and structural characterization of GSNO-HAs-Fe-HAP nanoparticle composite, followed by the investigation of nutrient release profiles, NO release kinetics, plant uptake studies, physiological and biochemical analyses and molecular analyses of GSNO-HAs-Fe-HAP NPs on maize under Cd stress, including their influence on growth performance, redox homeostasis, osmoprotectant regulation, secondary metabolism, phytochelatin synthesis, hormone signaling, gene expression, and soil health. This study advances the understanding of these multifunctional composites as next-generation fertilizers, while also evaluating their environmental safety and agricultural potential in promoting sustainable crop production.

## Results

### Synthesis and characterization of GSNO-HAs-Fe-HAP NPs

Surface modifications of HAP using HAs or metal phenole networks (MPNs) are known to induce distinct morphological and functional changes in these materials, allowing for the integration of complementary characteristics^11,13^. In this study, we developed a one-pot synthesis strategy to engineer the HAP by incorporating GSNO, soluble HAs, and ferric ions under alkaline conditions, resulting in the formation of GSNO-HAs-Fe-HAP nanoparticle composites.

Transmission electron microscopy (TEM) revealed that the particles consisted of approximately 50 nm primary nanoparticle composites that were highly aggregated into irregular clusters. The primary particles appeared as dark, electron-dense domains with roughly spherical morphologies, which were likely attributable to HAP. GSNO-HAs-Fe-HAP NPs possess a large surface area and an agglomerated structure, indicative of interparticle bonding (Supplementary Fig. 1).

SEM images revealed globular particles connected to wedge-shaped aggregates, consistent with the TEM observations. GSNO-HAs-Fe-HAP NPs were uniformly distributed, with sizes ranging from 10 to 100 nm and surfaces characterized by rough textures, protrusions, and distinct patterns (Supplementary Fig. 2A‒D). Considering the molecular formulas of GSNO and HAP, the significant S and Fe signals detected by EDX further confirmed the presence of GSNO and Fe on the surface of GSNO-HAs-Fe-HAP NPs, whereas the O, P, and Ca signals were likely derived from HAP (Supplementary Fig. 2E–G).

**Fig. 1:**
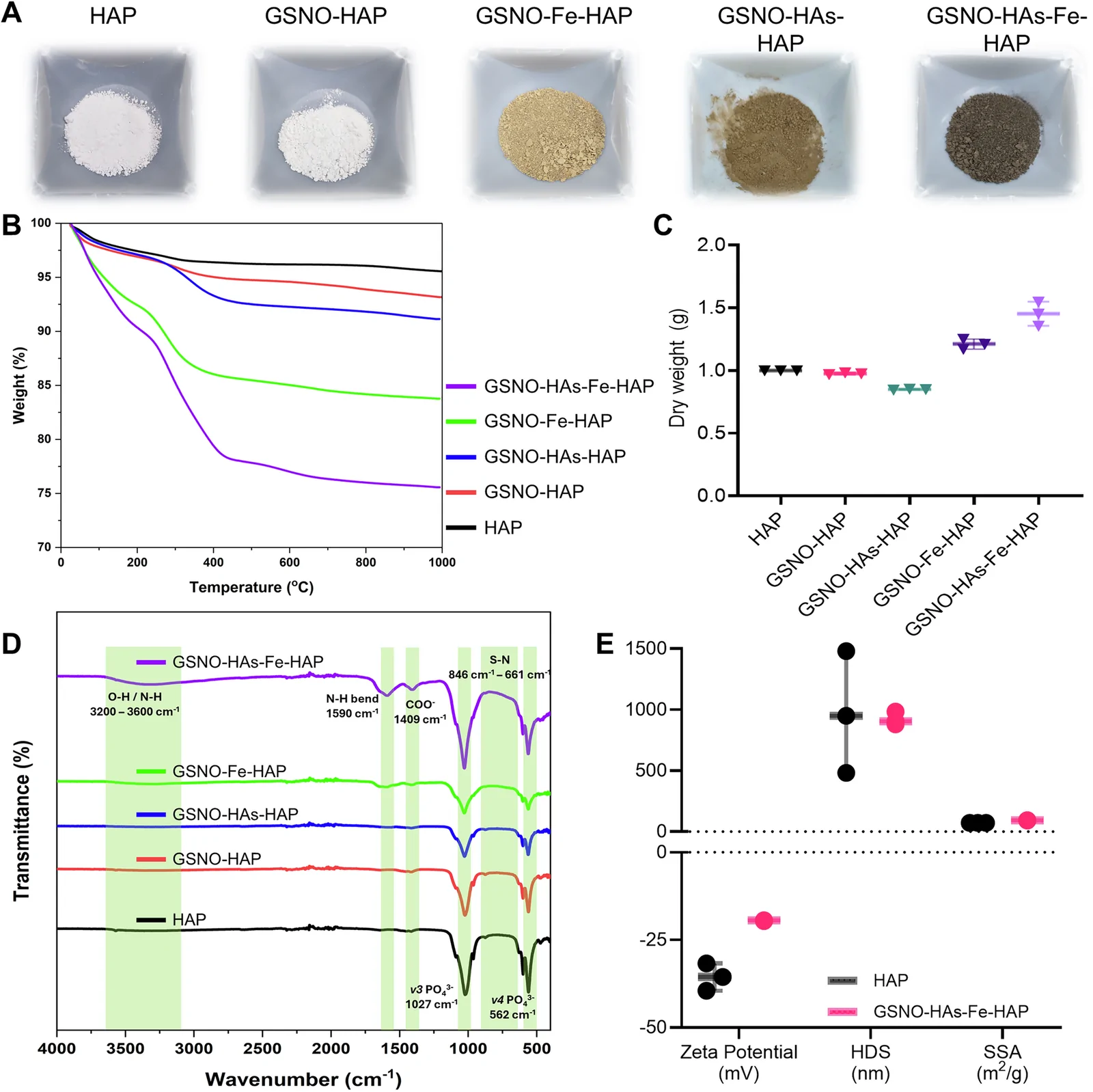
One-pot synthesis and characterization of GSNO-HAs-Fe-HAP nanoparticle composite. Characterization of synthesized hydroxyapatite (HAP)-based composites. **A** Photographic images showing visible color changes in HAP upon functionalization with S-nitroso glutathione (GSNO), humic acids (HAs), and/or Fe ions. **B** Results of thermogravimetric analysis (TGA) showing mass changes indicative of organic compound incorporation. **C** Total dry weight measurements demonstrating increased formation of coating layers on GSNO-HAs-Fe-HAP. **D** Fourier transform infrared (FT-IR) spectra confirm the presence of characteristic functional groups from GSNO and the phosphate in HAP. **E** Zeta potential, hydrodynamic size (HDS, Z-average), and specific surface area (SSA) based on Brunauer–Emmett–Teller (BET) analysis, indicating preserved structural characteristics of HAP post-modification. The experiment was performed independently in triplicate. Data are presented as mean ± standard deviation (*n* = 3).

As shown in Fig. 1A, bare HAP and GSNO-HAP particles appeared white, whereas GSNO-HAs-HAP and GSNO-HAs-Fe-HAP were brown and dark-brown, respectively, suggesting the successful integration of the dark-colored humic substances^11,13^. On the other hand, GSNO-Fe-HAP composites were light brown (Fig. 1A).

TGA was used to characterize the coating layers formed on HAP, which were composed of Fe, GSNO, and/or HAs. Differences in the weight loss associated with organic matter decomposition at 200 °C–600 °C and in the S content among HAP, GSNO-HAP, and the other composites (Fig. 1B, C and Supplementary Table 3) suggest that GSNO alone has a limited ability to attach to the HAP surface. Similarly, the addition of HAs alone resulted in negligible GSNO attachment. In contrast, ferric ions promoted GSNO recruitment, possibly through Fe-mediated bridging between the HAP surface and oxygen-containing functional groups of GSNO. When HAs and Fe were co-treated, the amount of attached GSNO was approximately 1.4-fold higher than that achieved with Fe alone, suggesting that Fe–HA interactions may further enhance the physical trapping of GSNO. Furthermore, a marked increase in the total dry weight of GSNO-HAs-Fe-HAP NPs was observed compared with the other composites (Fig. 1C), further supporting the efficient attachment of organic molecules, including GSNO and HAs, and Fe onto the HAP surface.

The carbon contents determined by elemental analysis were consistent with the TGA results (Supplementary Table 2), supporting the efficient Fe- and HA-mediated coating of GSNO onto HAP. FT-IR spectroscopy showed distinct GSNO-associated peaks in GSNO-HAs-Fe-HAP, including N–H stretching at 1590 cm^−1^, COO^−^ stretching at 1409 cm^−1^, and S–N stretching at 846 cm^−1^. These spectral signatures support the successful incorporation of GSNO in the presence of Fe ions and HAs. Peaks corresponding to phosphate groups, including ν_3_ and ν_4_ PO_4_^3−^ at 1027 and 562 cm^−1^, respectively, were also observed, confirming the presence of HAP (Fig. 1D). Zeta potential and hydrodynamic size measurements demonstrated that the one-pot synthesis approach did not compromise the structural integrity or particle size of the original HAP matrix (Fig. 1E).

### Behavior and release kinetics of GSNO-HAs-Fe-HAP NPs

**Fig. 2:**
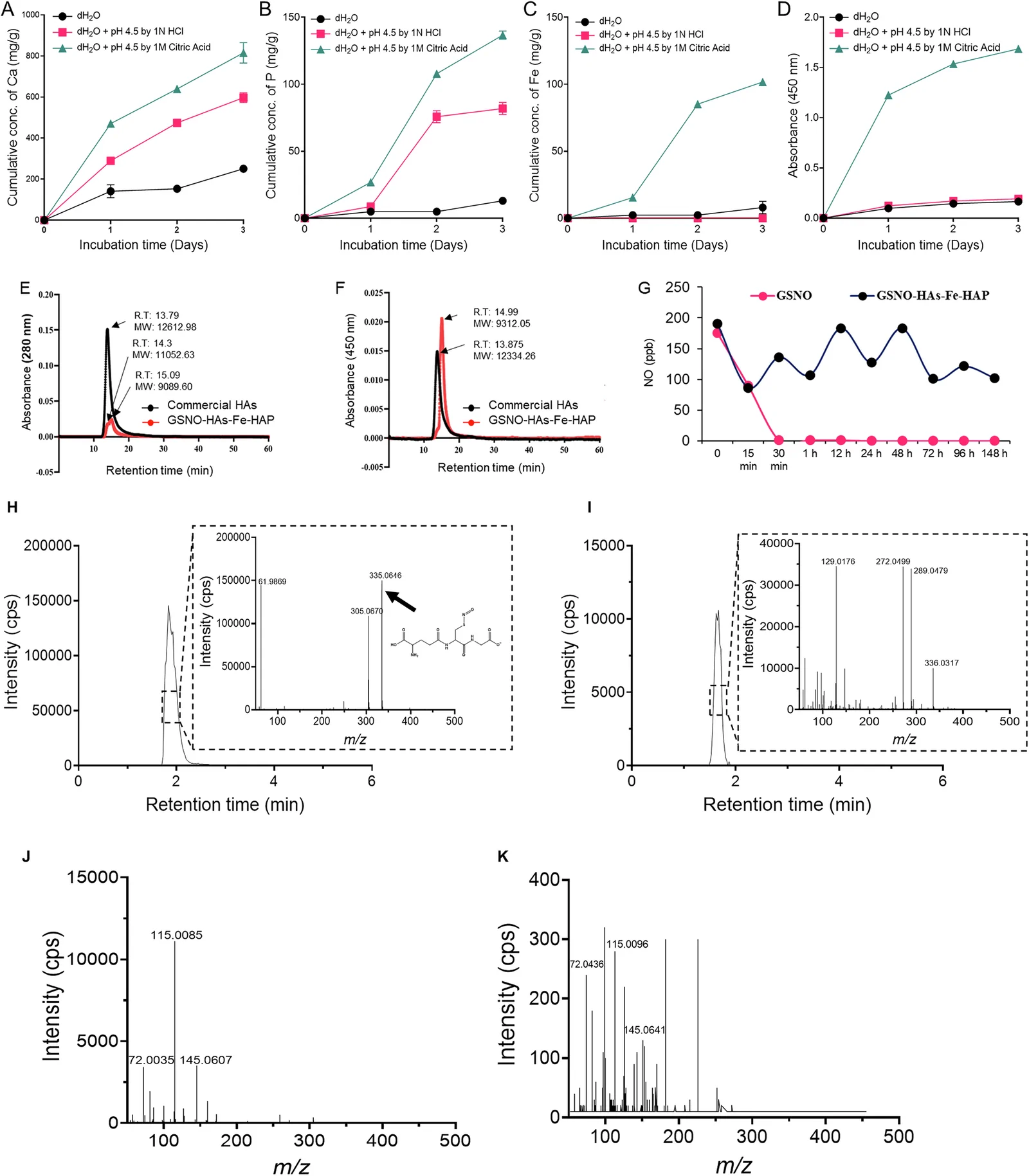
Organic acid-responsive release and molecular characterization of GSNO-HAs-Fe-HAP. Time-dependent release profiles of **A** calcium ions, **B** phosphate, and **C** ferric ions from GSNO-HAs-Fe-HAP under different acidic treatments: deionized water (control), HCl (pH 4.5), and citric acid (pH 4.5). Nutrient concentrations were cumulatively quantified at each time point (mean ± SD, *n* = 3). **D** Cumulative release of HAs as measured by UV absorbance at 450 nm. **E**, **F** Size exclusion chromatography (SEC) chromatograms of released HAs monitored at **E** 280 nm and **F** 450 nm, showing comparable molecular weights to commercial HAs. **G** NO release kinetics from free GSNO and GSNO-HAs-Fe-HAP measured over time using a Sievers NOA-280i NO analyzer. **H–K** Liquid chromatography–electrospray ionization– quadrupole-time-of-flight mass spectrometry (LC-ESI-Q-TOF MS) analysis of GSNO transformation: **H**, **I** *m/z* values detected in the LC peak of **H** standard GSNO and **I** GSNO-HAs-Fe-HAP; **J**, **K** Tandem mass spectrometry (MS/MS) spectrum of standard GSNO **J** and supernatants from GSNO-HAs-Fe-HAP **K** indicating structural transformation and NO release. HAs humic acids, GSNO-HAs-Fe-HAP HAP coated with HAs, ferric ions, and S-nitrosoglutathione (GSNO), MW molecular weight, R.T. retention time. The experiment was performed independently in triplicate. Data are presented as mean ± standard deviation (*n* = 3).

Modern-day agriculture is highly dependent upon the efficient release of essential nutrients from fertilizers. Synthetic fertilizers, or nutrient composites, disassemble and release nutrients into the soil profile under a variety of conditions and in response to various cues. Root exudate-triggered disassembly of lignin-Fe-HAP supramolecular structures has been previously demonstrated with the co-release of essential crop nutrients (e.g., calcium, phosphorus, and ferric ions) and stimulants (e.g., lignin-derived organics) in a time-dependent manner, outperforming conventional NPK fertilizers, especially under stress conditions^13^. Here, we quantitatively evaluated the release kinetics of both nutrients (Ca, P, and Fe) and stimulants (HAs and NO) from the GSNO-HAs-Fe-HAP NP composite under acidic conditions (pH 4.5), using either citric acid or HCl as the acidic trigger (Fig. 2A−D). Results indicated that citric acid resulted in the release of approximately 500 mg of calcium (Ca) from one gram of the nanoparticle composite after 24 hours, which later increased up to 800 mg after three days (Fig. 2A), Similarly, 135 mg phosphorus (P) and 100 mg iron (Fe) were released per gram of the composite (Fig. 2B, C). Interestingly, the release kinetics followed the order of citric acid > HCl > distilled water. The slowest release in distilled water could be attributed to the limited solubility of HAP under non-acidic conditions, whereas the faster release in citric acid than in HCl may be associated with the metal-chelating ability of citric acid. Given that citric acid is one of the major root exudates^13^, this trend suggests that GSNO-HAs-Fe-HAP particles may release nutrients efficiently in the rhizosphere.

HAs (Humic acids) are chromogenic polymers formed in the soil after microbial degradation of plant and animal matter, a process called humification. Humus is critical for soil fertility, plant growth, and soil remediation. We thus analyzed the GSNO-HAs-Fe-HAP nanoparticle composite for the release of HAs. UV-Vis and size-exclusion chromatography (SEC) analyses confirmed that the increase in absorbance at 450 nm following acidic  environment was predominantly due to released HAs (Fig. 2D). SEC results demonstrated that the eluted HAs from GSNO-HAs-Fe-HAP had molecular weights (~10,000 Da) comparable to those of a HA-based commercial product. Furthermore, dual-wavelength absorbance at 280 nm and 450 nm supports the successful release of bioavailable humic components, with negligible interference from GSNO-derived color (Fig. 2E, F).

Given the critical role of ferric ions in GSNO immobilization^26,27^, and the presence of electron-donating groups in humic acids^28^, we hypothesized that released HAs may also mediate GSNO reduction, thereby contributing to NO release. This was confirmed using highly sensitive ozone-chemiluminescence-based NO measurements. While free GSNO rapidly depleted and released its NO within 30 min, an equimolar amount of GSNO-HAs-Fe-HAP exhibited a ~ 10-fold increase in cumulative NO release, sustained over 148 h (Fig. 2G).

To examine potential GSNO transformation during release, liquid chromatography–electrospray ionization–time of flight (LC-ESI-Q-TOF MS) analysis was employed (Fig. 2H−K). The ion peaks at *m/z* 335.0646 and 305.0670 were assigned to [GSNO − H]^−^ and [GSNO − H − NO]^−^, respectively, in the standard GSNO solution (Fig. 2H), whereas these peaks were not detected in the supernatants of GSNO-HAs-Fe-HAP. Instead, ions at *m/z* 336.0317, 289.0479, and 272.0499 were detected (Fig. 2I). Among these, the ion at *m/z* 336.0317 may originate from a modified or reduced form of GSNO, potentially due to the electron-donating capacity of HAs. The partial overlap in MS/MS fragmentation patterns between GSNO-HAs-Fe-HAP and standard GSNO, including fragment ions at *m/z* 72.0436, 115.0096, and 145.0641, suggests that GSNO was partially transformed in GSNO-HAs-Fe-HAP (Fig. 2J, K). This transformation may be associated with the long-term release of NO.

### *In planta* Uptake and distribution of GSNO-HAs-Fe-HAP NPs

**Fig. 3:**
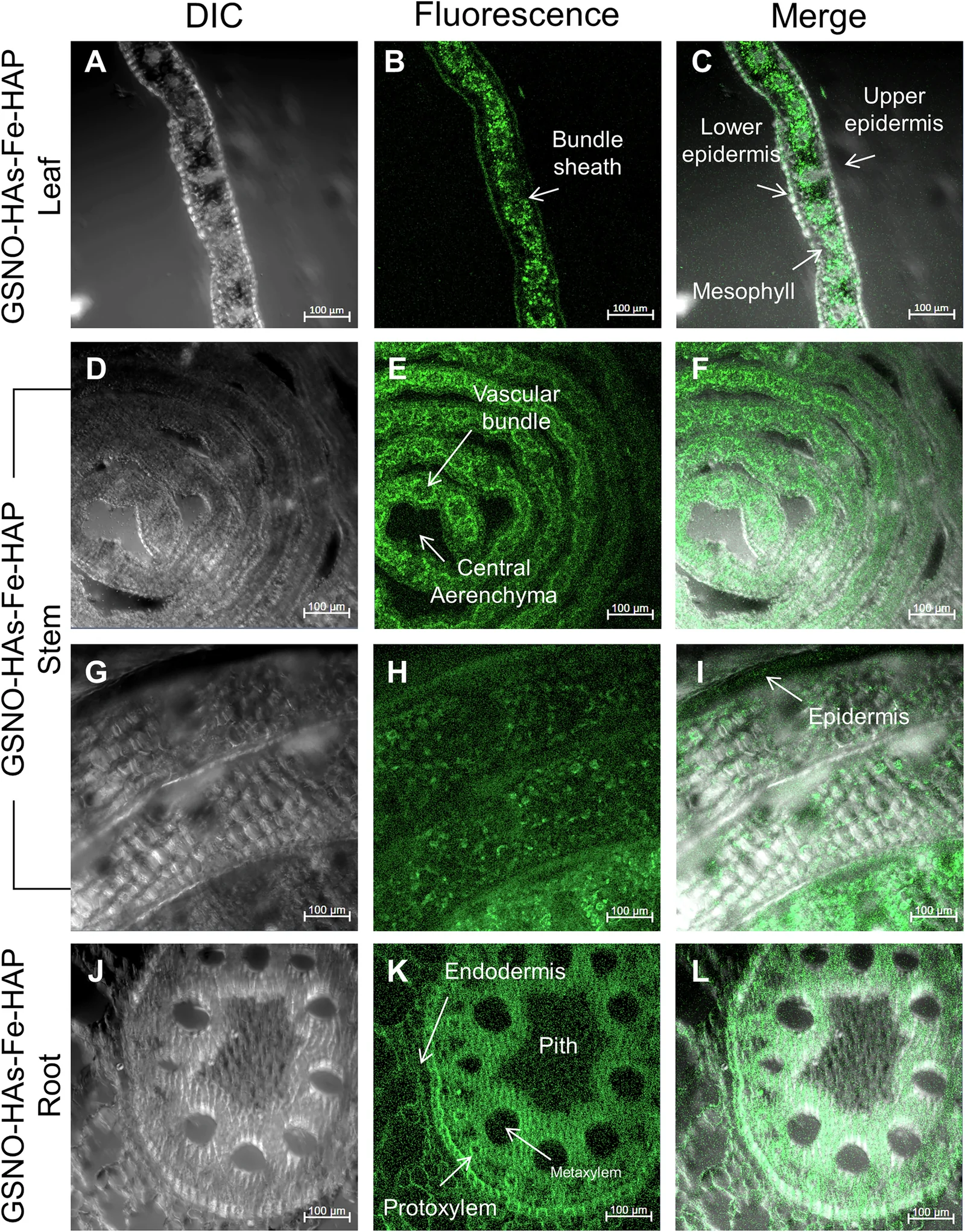
Uptake and distribution of GSNO-HAs-Fe-HAP NPs in maize seedlings. Accumulation of GSNO-HAs-Fe-HAP NPs in leaf (**A–C**), stem (**D–I**), and root tissues (**J–L**) of maize seedlings exposed to Cd toxicity for 2 weeks, visualized using differential interference contrast (DIC) and confocal microscopy techniques. Green fluorescence from the fluorescein isothiocyanate (FITC) indicates the localization of GSNO-HAs-Fe-HAP NPs within plant tissues. The experiment was performed using three independent biological replicates, each comprising a different individual plant, and similar localization patterns were observed in all three replicates. Representative images from one biological replicate are shown.

The *in planta* uptake and localization of GSNO-HAs-Fe-HAP NPs from soil were tested in maize seedlings using fluorescein isothiocyanate (FITC) labeling (Fig. 3). The upper and lower epidermis, bundle sheath, xylem, and phloem (Fig. 3A‒C), stem (rolled leaf buds, vascular bundles, ground tissue, and central aerenchyma) (Fig. 3D‒I), and roots (endodermis, pericycle, metaxylem, protoxylem, and phloem) (Fig. 3J‒L) were observed via differential interference contrast (DIC) microscopy. Confocal imagery indicated significant accumulation of GSNO-HAs-Fe-HAP NPs in all the plant tissues, indicating effective absorption and localization of the NP composite. The combination of DIC and FITC-labeled confocal imaging facilitated enhanced visualization of NP transport from root to leaf.

### Effect on growth and photosynthesis under Cd tolerance index

**Fig. 4:**
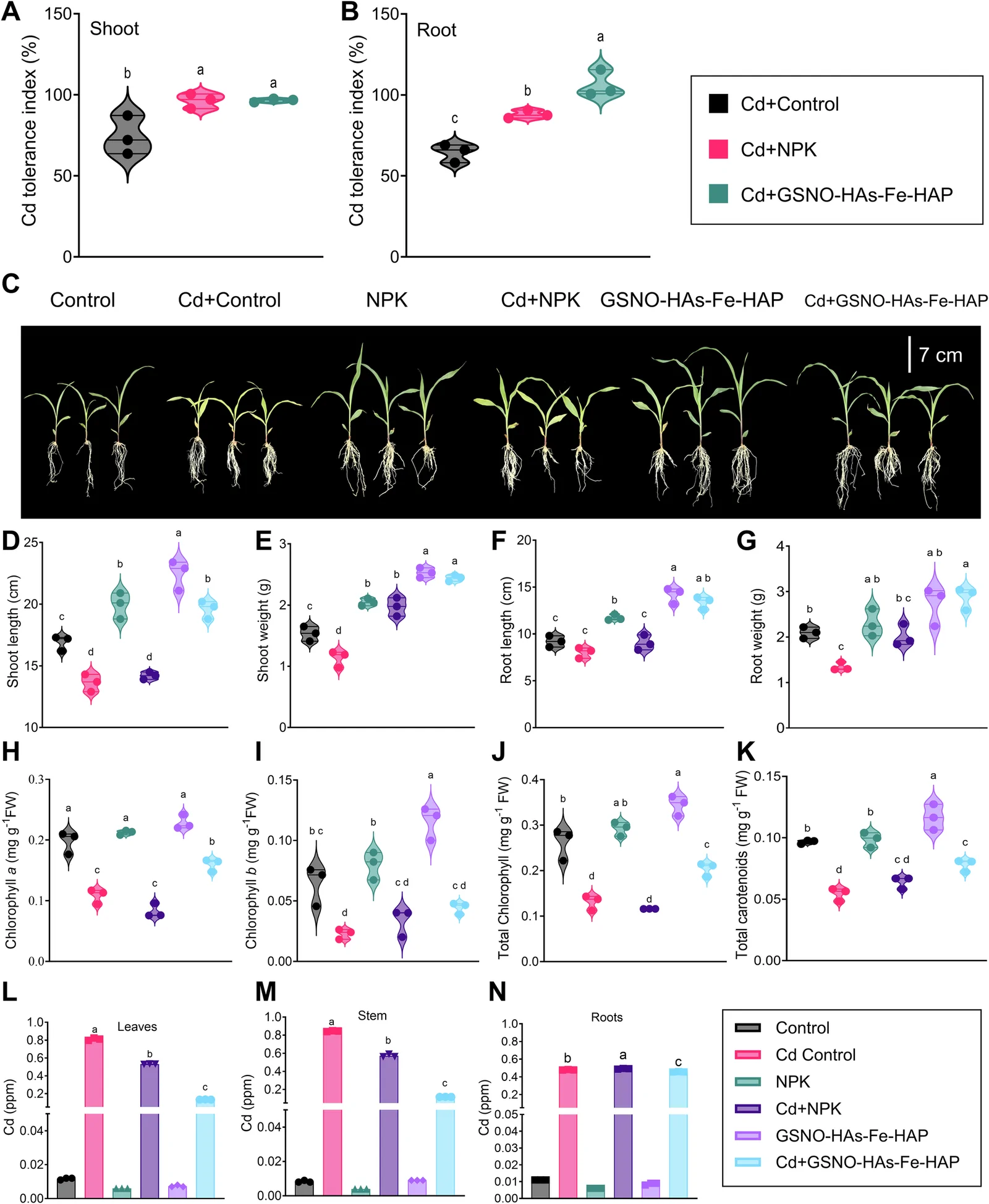
Impact of GSNO-HAs-Fe-HAP NPs on maize growth and Cd accumulation in plant tissues. The effects of GSNO-HAs-Fe-HAP NPs on maize growth and Cd tolerance were evaluated after 2 weeks of Cd stress by determining the shoot Cd tolerance index (**A**), root Cd tolerance index (**B**), shoot length (**D**), shoot fresh weight (**E**), root length (**F**), root fresh weight (**G**), chlorophyll a content (**H**), chlorophyll b content (**I**), total chlorophyll content (**J**), and total carotenoid content (**K**). **C** shows representative maize seedlings from the different treatment groups. Cd concentrations were quantified in leaves (**L**), stems (**M**), and roots (**N**). Data are presented as the mean ± standard error of three independent biological replicates per treatment (*n* = 3). For the morphological parameters in (**D–G**), we measured 12 seedlings per biological replicate and averaged them to generate one replicate value. The image in (**C**) is representative of three independent biological replicates with similar results. Quantitative data in (**A**,**B**) and (**D–N**) were analyzed using ordinary one-way ANOVA followed by two-sided Tukey’s all-pairs comparisons. *P* values were adjusted for multiple comparisons using Tukey’s method. Different lowercase letters indicate statistically significant differences at Tukey-adjusted *P* < 0.05. Omnibus ANOVA *P* values were: **A** *P* = 0.0159; **B** *P* = 0.0004; **D** *P* = 8.390286 × 10^-8^; **E** *P* = 1.142994 × 10^-8^; **F** *P* = 2.653127 × 10^-7^; **G** *P* = 0.0001561; **H** *P* = 1.687555 × 10^-8^; **I** *P* = 3.488595 × 10^-6^; **J** *P* = 3.819092 × 10^-8^; **K** *P* = 2.714461 × 10^-7^; **L** *P* = 1.696214 × 10^-21^; **M** *P* = 1.075415 × 10^-20^; and **N** *P* = 8.652659 × 10^-25^.

To quantitatively assess the actual severity of sublethal Cd stress and evaluate the protective efficacy of the treatments, we first determined the cadmium tolerance index of the plants at the V5 vegetative stage. Results indicated a significantly higher Cd tolerance index in shoots and roots treated with the NP composite compared with control plants (Fig. 4A, B). Following this, the practical applicability and impact of the nanoparticle composite on plant growth and development were evaluated using maize seedlings under cadmium (Cd) stress, and the effects were compared to those of commercial NPK fertilizer. As expected, Cd exposure led to significantly stunted growth and chlorosis (Fig. 4C), consistent with the literature^29^. In contrast, seedlings treated with GSNO-HAs-Fe-HAP NPs exhibited significantly robust growth compared to both NPK-treated and control plants, both in the presence and absence of Cd stress (Fig. 4C). Quantitative analysis confirmed these observations. In the absence of Cd stress, shoot length and fresh weight increased by 17.9% and 32.9% in NPK-treated plants, whereas GSNO-HAs-Fe-HAP NP-treated plants demonstrated even greater increases of 33.8% and 65.0%, respectively, compared to control plants (Fig. 4D, E). Under Cd stress, GSNO-HAs-Fe-HAP NP-treated plants showed minimal reductions in shoot length (3.5%) and fresh weight (3.4%), while NPK-treated plants exhibited significant reductions (28.7% and 12.7%, respectively). Similar trends were observed for the length and fresh weight of roots (Fig. 4F, G), highlighting the superior resilience conferred by GSNO-HAs-Fe-HAP NPs.

Cd stress is known to inhibit chlorophyll synthesis and impair photosynthetic efficiency by promoting reactive oxygen species (ROS) accumulation, thereby disrupting the photosynthetic electron transport chain and decreasing ATP and NADPH production^30^. However, GSNO-HAs-Fe-HAP NP-treated plants exhibited significantly higher levels of chlorophyll a, b, total chlorophyll, and carotenoids under Cd stress than those treated with NPK fertilizers (Fig. 4H−K), indicating strong protective effects. Treatment with GSNO-HAs-Fe-HAP NPs markedly reduced Cd accumulation in maize tissues. Specifically, 83.8% and 86% lower Cd concentrations were recorded in the leaves and stem, respectively, when plants were treated with the NP composite in the presence of 400 µM Cd (Fig. 4L, M). On the other hand, 4.8% less Cd was recorded in the roots, which, although modest, still outperformed the 2.6% increase observed in NPK-treated seedlings under Cd stress (Fig. 4N).

Similar experiments were set up in combination with the NO scavenger cPTIO, where the Cd and GSNO-HAs-Fe-HAP NPs were applied with and without cPTIO, and data on the same parameters were recorded as described above (Supplementary Fig. 3). Data on shoot/root length (Supplementary Fig. 3A–C), shoot/root weight (Supplementary Fig. 3D, E), chlorophyl a & b (Supplementary Fig. 3F, G), *Fv/Fm* and carotenoid content (Supplementary Fig. 3H, I) clearly indicated that the previously recorded highest values for these parameters were indeed due to the impact of NO, as cPTIO application resulted in a significant quantitative reduction in all these parameters of the plants treated with cPTIO+Cd+GSNO-HAs-Fe-HAP.

### Effect on redox homeostasis in maize under Cd stress

**Fig. 5:**
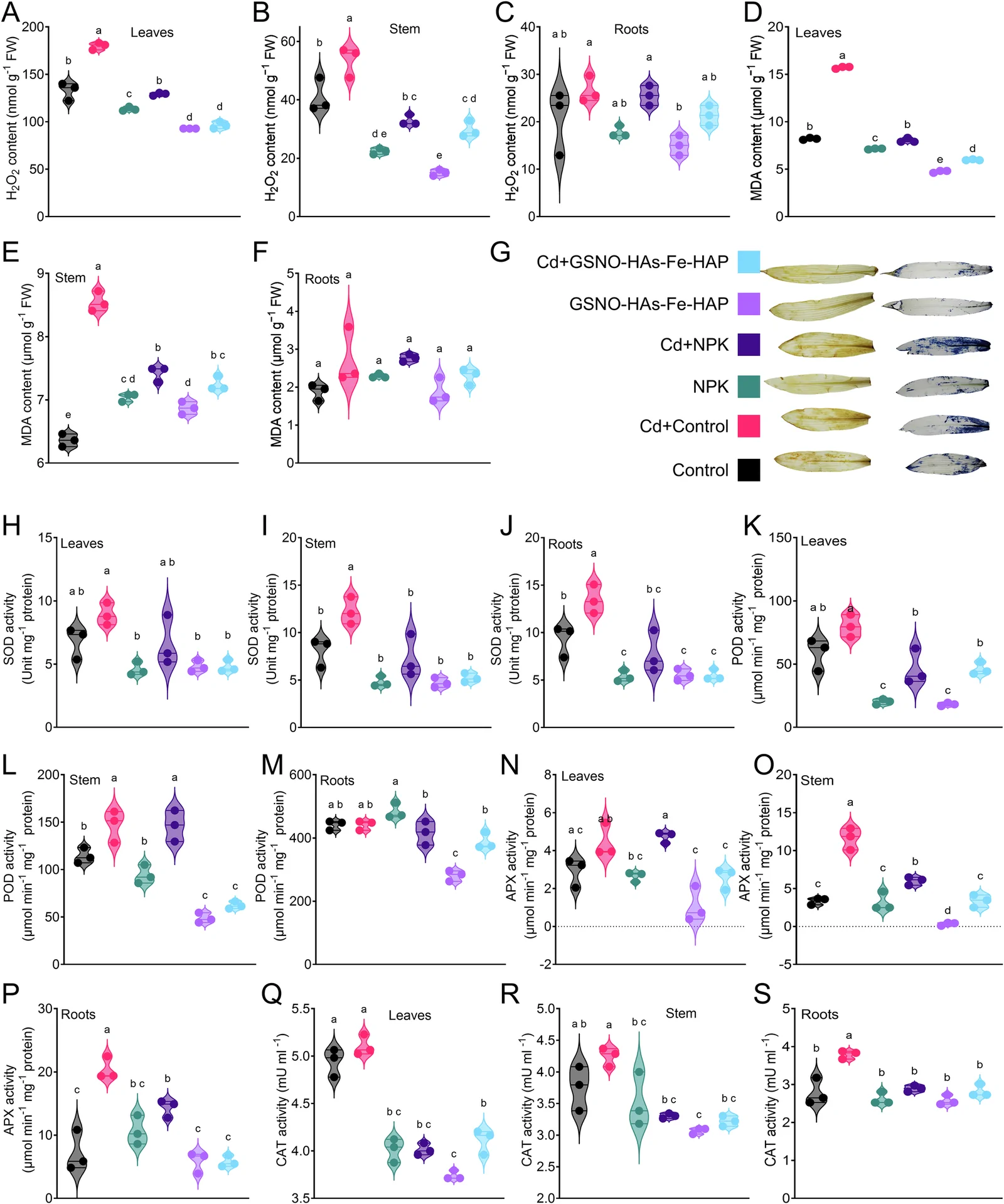
GSNO-HAs-Fe-HAP NPs regulate redox homeostasis in maize under Cd stress. Effects of GSNO-HAs-Fe-HAP NPs on the redox homeostasis of maize seedlings under Cd stress were determined by measuring H_2_O_2_ (**A–C**) and MDA contents (**D–F**) in the leaves, stem, and roots of maize plants. Panel **G** shows representative DAB and NBT histochemical staining images indicating H₂O₂ and superoxide accumulation, respectively, and was not subjected to inferential statistical analysis. The effects of the nanoparticle composite on antioxidant enzyme activities in maize seedlings exposed to Cd toxicity for 2 weeks were determined by measuring the activity of SOD, (**H–J**), POD, (**K–M**), APX (**N–P**), and CAT (**Q–S**) in the leaves, stem, and roots of maize seedlings. Data are presented as mean ± standard error of three independent biological replicates per treatment (*n* = 3). Each biological replicate corresponded to one independently grown and treated maize plant maintained in a separate pot. Tissue from each plant was collected, processed, and analyzed separately, and samples from different plants were not pooled. Quantitative data in (**A–F**) and (**H–S**) were analyzed using ordinary one-way ANOVA followed by two-sided Tukey’s all-pairs comparisons. *P* values were adjusted for multiple comparisons using Tukey’s method. Different lowercase letters indicate statistically significant differences at Tukey-adjusted *P* < 0.05. Exact omnibus ANOVA *P* values were: **A** *P* = 2.226274 × 10^−10^; **B** *P* = 2.179317 × 10^−7^; **C** *P* = 0.0093; **D** *P* = 7.686294 × 10^−18^; **E** *P* = 6.225140 × 10^−10^; **F** *P* = 0.0320696; **H** *P* = 0.0022333; **I** *P* = 8.736856 × 10^−5^; **J** *P* = 4.982048 ×  10^−5^; **K** *P* = 1.355071 × 10^−5^; **L** *P* = 3.984586 × 10^−7^; **M** *P* = 5.010835 × 10^−6^; **N** *P* = 0.0002292; **O** *P* = 6.356692 × 10^−8^; **P** *P* = 6.319238 × 10^−6^; **Q** *P* = 1.007829 × 10^−8^; **R** *P* = 0.0006382; and **S** *P* = 4.493621 × 10^−5^.

Plants exposed to environmental stress often generate ROS, which can accumulate and trigger oxidative stress. Under Cd stress, excess electrons from the photosynthetic electron transport chain react with molecular oxygen (O_2_), resulting in the formation of superoxide (O_2_^•‒^) anions, which contribute to oxidative damage and cellular dysfunction^31–33^. In this study, ROS accumulation in Cd-exposed maize seedlings was assessed through histochemical staining and quantitative analysis (Fig. 5). Cd toxicity markedly elevated H_2_O_2_ and O_2_^•‒^ levels in the leaves, stem, and roots of maize plants (Fig. 5A–C). However, Cd-stressed plants treated with the GSNO-HAs-Fe-HAP composites produced the lowest hydrogen peroxide. On the other hand, Cd toxicity is also known to elevate malondialdehyde (MDA) levels—a key biomarker of lipid peroxidation and the resulting membrane damage and protein denaturation^34^. Under cadmium stress, the MDA content in the leaves, stem, and roots was markedly lower in GSNO-HAs-Fe-HAP NP-treated plants compared to those treated with NPK (Fig. 5D‒F), indicating improved oxidative stress tolerance. These measurements were corroborated by intense brown spots in 3,3-diaminobenzidine (DAB) staining and pronounced dark blue deposits in nitroblue tetrazolium (NBT) staining of leaves (Fig. 5G). However, treatment with GSNO-HAs-Fe-HAP NPs under Cd stress substantially reduced ROS-associated staining and significantly lowered endogenous H_2_O_2_ levels compared to NPK-treated seedlings. This suggests that GSNO-HAs-Fe-HAP NPs effectively attenuate ROS generation. Parallel to these findings, another experiment involving treatment of the plants with GSNO-HAs-Fe-HAP NPs in the presence of the NO scavenger cPTIO resulted in a significant increase in the leaf, stem, and root H_2_O_2_ content (Supplementary Fig. 4A–C), and MDA contents (Supplementary Fig. 4D–F).

Plants rely on antioxidant enzymes, including superoxide dismutase (SOD), peroxidase (POD), ascorbate peroxidase (APX), and catalase (CAT), to remove ROS and regulate redox homeostasis under stress conditions, thereby minimizing cellular damage. The activities of these key antioxidant enzymes were analyzed in the leaves, stem, and roots of maize seedlings, along with real-time quantification of their corresponding transcripts during Cd stress (Supplementary Fig. 4A‒L). Interestingly, the activities of these key antioxidant enzymes were evaluated in the leaves, stem, and roots of maize seedlings treated with NPK or GSNO-HAs-Fe-HAP NPs under Cd stress.

In the Control group, SOD activity in the leaf showed no significant difference; however, compared to the Cd Control, activity was elevated by 51.9% in the stem and 44.7% in the root, indicating a tissue-specific antioxidative response under cadmium stress conditions. In contrast, treatments with either NPK or GSNO-HAs-Fe-HAP NPs did not result in significant alterations in SOD activity across all examined tissues under Cd exposure (Fig. 5H–J).

Regarding POD activity, the Control treatment led to a 28.7% increase in the stem relative to the Cd Control, whereas the leaf and root showed no significant differences. Under Cd stress, NPK application significantly enhanced POD activity by 129.5% in the leaves (Fig. 5K) and 55.2% in the stem (Fig. 5L) compared to the Cd+NPK group, but resulted in a 14% decrease in the root (Fig. 5M). In contrast, GSNO-HAs-Fe-HAP NPs treatment markedly increased POD activity by 154.1% in the leaves and 38.1% in the roots, with no significant change observed in the stem.

For APX activity, the leaves showed no significant difference between the Control and Cd Control treatments, but the lowest APX activity was recorded in plants treated with the nanoparticle composite (Fig. 5N). However, activity in the stem and roots increased by 244.7% and 184.0%, respectively (Fig. 5O, P) in response to Cd stress. Consistent with previous findings, nanoparticle composite application resulted in the lowest APX activity in all tissues, both in the absence and presence of cadmium stress. More specifically, the NPK treatment significantly elevated APX activity by 78.8% in the leaves and 88.5% in the stem compared to the Cd+NPK group. At the same time, no significant change was observed in the roots. In the GSNO-HAs-Fe-HAP-treated plants, APX activity remained unchanged in the leaves and roots compared to the Cd+GSNO-HAs-Fe-HAP NP-treated plants; however, a dramatic increase of 894.1% was observed in the stem (Fig. 5N–P).

Catalase activity was measured in the leaves (Fig. 5Q), stem (Fig. 5R), and roots (Fig. 5S). In the Control group, CAT activity in the stem increased by 13.1% compared to the Cd Control, while no significant differences were observed in the leaves and roots. NPK treatment did not induce any significant changes in CAT activity across all tissues compared to the Cd+NPK group. In contrast, under GSNO-HAs-Fe-HAP NPs treatment, CAT activity exhibited a modest increase of 9.9% in the leaves relative to the Cd+GSNO-HAs-Fe-HAP NPs group. In contrast, no significant alterations were detected in the stem and root.

GSNO-HAs-Fe-HAP NPs treatment in combination with the NO scavenger cPTIO resulted in a significant increase in the activity of antioxidant enzymes compared to GSNO-HAs-Fe-HAP NPs treatment alone. This involved a statistically significant increase in leaf/stem/root activity of SOD (Supplementary Fig. 4G–I), POD (Supplementary Fig. 4J–L), APX (Supplementary Fig. 4M–O), and CAT activity (Supplementary Fig. 4P–R).

Quantitative real-time PCR analysis indicated that in the Cd-stressed control seedlings, the relative expression of genes encoding the SOD (Supplementary Fig. 5A‒C), POD (Supplementary Fig. 5D‒F), APX (Supplementary Fig. 5G‒I), and CAT (Supplementary Fig. 5J‒L) was significantly upregulated in the leaves, stem, and roots, reflecting the oxidative burden observed earlier. Conversely, both NPK- and GSNO-HAs-Fe-HAP NP-treated plants displayed noticeably reduced expression levels, indicating relief from oxidative pressure. Together with the lower Cd accumulation and ROS content, these results suggest that GSNO-HAs-Fe-HAP NPs alleviate oxidative stress and lessen the demand for elevated antioxidant enzyme activity. These findings highlight their role in modulating redox homeostasis in maize seedlings under Cd stress.

### GSNO-HAs-Fe-HAP NPs enhance osmolytes under Cd stress

Plants accumulate osmolytes, also known as compatible solutes, to maintain cellular homeostasis under abiotic stress, to stabilize proteins, membranes, and cellular structures while balancing osmotic pressure. Therefore, proline, total protein, carbohydrates, and soluble sugars were quantified in maize seedlings exposed to Cd stress and treated with either NPK fertilizers or GSNO-HAs-Fe-HAP NPs. Under Cd stress, GSNO-HAs-Fe-HAP NPs markedly elevated proline levels in leaves and roots. Specifically, proline content in the leaves increased by 75.2% compared to the Cd Control and by 37.3% relative to the Cd+NPK group (Supplementary Fig. 6A). Though slight, significantly different changes in proline content were also observed in the stem (Supplementary Fig. 6B). In the roots, proline levels were elevated by 196.7% compared to the Cd Control and by 76.3% compared to the Cd+NPK (Supplementary Fig. 6C), suggesting that GSNO-HAs-Fe-HAP NPs confer a stronger osmoprotective response under Cd stress than conventional fertilizer.

**Fig. 6:**
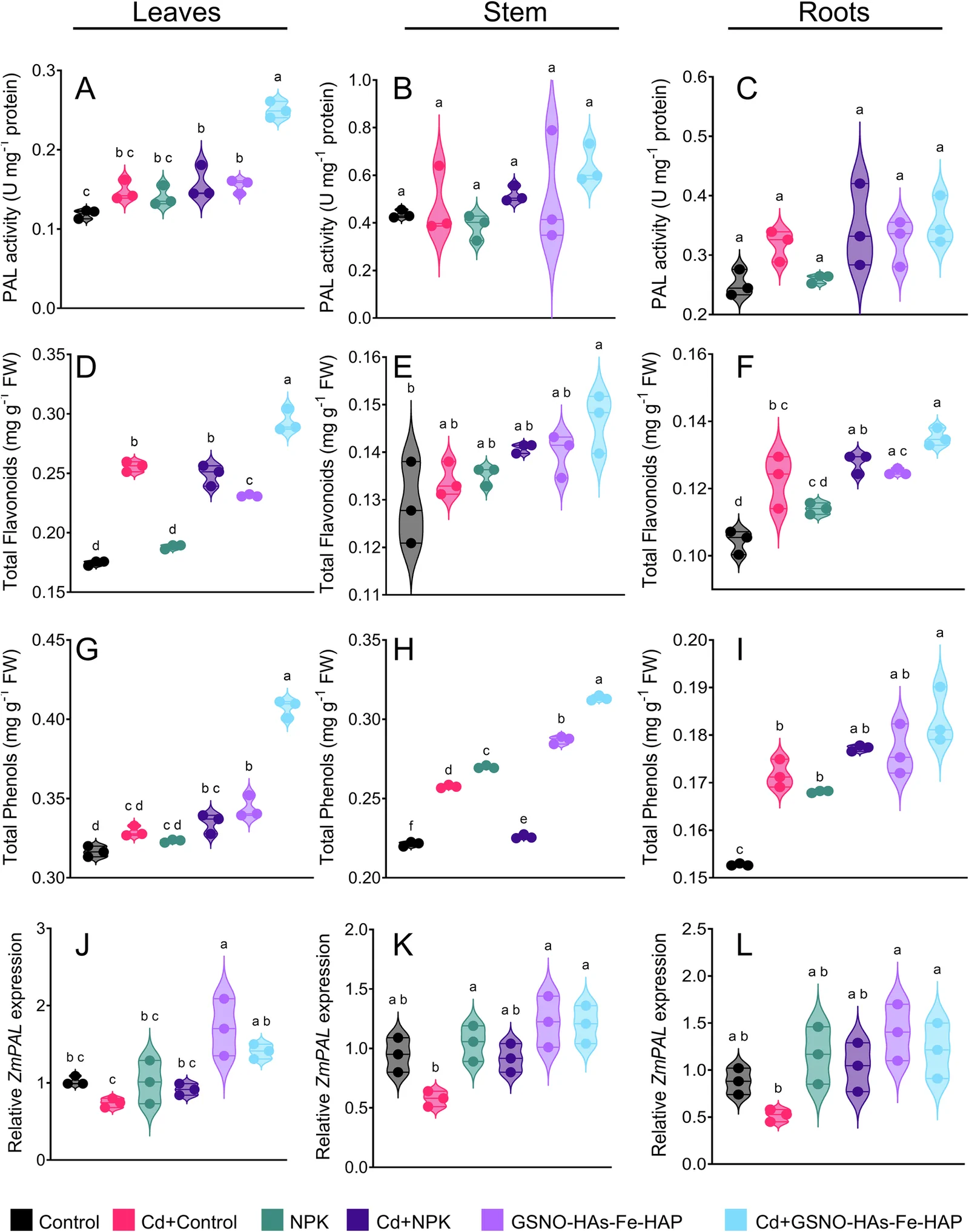
Impact of GSNO-HAs-Fe-HAP NPs on secondary metabolite biosynthesis in maize seedlings under Cd stress. The effects of the nanoparticle composite on the biosynthesis of secondary metabolites were determined by measuring phenylalanine ammonia-lyase (PAL) activity (**A–C**), total flavonoids (**D–F**), and total phenols (**G–I**) and expression of *ZmPAL* (**J–L**), in the leaves, stem, and roots of maize seedlings. Data are presented as mean ± standard error of three independent biological replicates per treatment (*n* = 3). Each biological replicate corresponded to one independently grown and treated maize plant maintained in a separate pot. Tissue from each plant was collected, processed, and analyzed separately, and samples from different plants were not pooled. Data in (**A–L**) were analyzed using ordinary one-way ANOVA followed by two-sided Tukey’s all-pairs comparisons. *P* values were adjusted for multiple comparisons using Tukey’s method. Different lowercase letters indicate statistically significant differences at Tukey-adjusted *P* < 0.05. Omnibus ANOVA P values were: **A** *P* = 5.421207 × 10^−7^; **B** *P* = 0.2489894; **C** *P* = 0.0293616; **D** *P* = 6.907005 × 10^−11^; **E** *P* = 0.0146288; **F** *P* = 1.015214 × 10^−5^; **G** *P* = 3.597666 × 10^−10^; **H** *P* = 1.628083 × 10^−16^; **I** *P* = 3.034034 × 10^−6^; **J** *P* = 0.0009; **K** *P* = 0.0030; and **L** *P* = 0.0107.

Quantitative real-time PCR analysis revealed that the expression of *PYRROLINE-5-CARBOXYLATE REDUCTASE 1* (*ZmP5CR1*), a gene central to proline biosynthesis, was suppressed under Cd stress but increased by 124.6% in the stem tissues and 228.5% in the roots of Cd GSNO-HAs-Fe-HAP NP-treated plants compared to those of Cd Control (Supplementary Fig. 6M‒O). Cd stress reduced total protein, carbohydrate, and soluble sugar levels across all plant tissues. However, treatment with GSNO-HAs-Fe-HAP NPs significantly improved the levels of these osmolytes in comparison to both Cd-only and NPK treatments (Supplementary Fig. 6). Total protein content was highest in the leaves of plants treated with either NPK or GSNO-HAs-Fe-HAP NPs, with no statistically significant differences observed under Cd stress conditions (Supplementary Fig. 6D). However, in the stem of Cd GSNO-HAs-Fe-HAP NP-treated plants, protein content increased by 46.3% compared to the Cd+NPK (Supplementary Fig. 6E). Similarly, the highest total protein was recorded in the roots of maize plants treated with the nanoparticle composite Supplementary (Fig. 6F).

The total carbohydrate content was highest in all tissues of maize seedlings treated with GSNO-HAs-Fe-HAP NPs. Compared to Cd+NPK, carbohydrate content increased by 13.2% in the leaf, 14.0% in the stem, and 74.4% in the root (Supplementary Fig. 6G‒I), indicating a pronounced enhancement of carbon assimilation following the nanocomposite treatment. Similarly, the total soluble sugars in the Cd+GSNO-HAs-Fe-HAP NPs group were elevated relative to the Control, with increases of 15.1% in the stem and 22.4% in the root (Supplementary Fig. 6J‒L) compared to the Cd+NPK treatment, suggesting that GSNO-HAs-Fe-HAP NPs enhance carbohydrate metabolism and mobilization under Cd stress conditions. The NP composite also significantly improved the expression of the *ZmPCR1* gene in maize leaves, stems, and roots (Supplementary Fig. 6M–O).

### Effect on maize secondary metabolites under Cd stress

To further investigate stress mitigation, we analyzed the impact of GSNO-HAs-Fe-HAP NPs on phenylalanine ammonia-lyase (PAL) activity and the biosynthesis of key secondary metabolites, specifically phenols and flavonoids. PAL serves as a gateway enzyme for polyphenol biosynthesis and bridges primary and secondary metabolism^35^. Phenolic and flavonoid compounds function as potent antioxidants, mitigating oxidative damage by scavenging ROS, quenching singlet oxygen, and decomposing peroxides^36,37^. Additionally, flavonoids contribute to plant development by imparting pigmentation to flowers and seeds, facilitating pollination, and protecting against photo-oxidative damage^36^.

Phenylalanine ammonia-lyase (PAL) activity was significantly enhanced in the leaves of plants treated with Cd+GSNO-HAs-Fe-HAP NPs, showing a 69.4% increase compared to the Cd control and a 59.3% increase compared to the Cd+NPK group (Fig. 6A). However, no statistically significant differences in PAL activity were observed in the stem and root tissues (Fig. 6B, C).

Quantification of secondary metabolites indicated that the total flavonoid content in the leaves was elevated by 17.9% in the Cd+GSNO-HAs-Fe-HAP NPs relative to Cd + NPK treatment (Fig. 6D), whereas the highest quantity of flavonoids was detected in the stem and roots of plants treated only with the nanocomposite (Fig. 6E, F). Similarly, the quantity of total phenols was significantly higher in the leaves and stem of Cd+nanocomposite-treated plants, with increases of 21.7% and 38.7%, respectively (Fig. 6G, H), compared to Cd+NPK treatments. In contrast, no significant change was observed in the roots per the same comparison (Fig. 6I).

The enhancement of secondary metabolite production was not only at the translational level, but also at the transcript level as QPCR analysis showed a significant increase in the expression of *ZmPAL* in the leaves (Fig. 6J), stem (Fig. 6K), and roots (Fig. 6L).

In-depth MS/MS-LC-ESI-Q-TOF-based metabolite profiling further confirmed that GSNO-HAs-Fe-HAP composite drastically enhanced the total content of various secondary metabolites, including 22 phenols & flavonoids, 14 amino acids, six fatty acids & lipids, and six alkaloids, compared to only two, one, two, and one each of these compounds, respectively in the control plants (Supplementary Fig. 7), beside several other steroids, terpenoids, purines, and pyrimidines, with the inpending Cd stress, indicating a robust activation of metabolic pathways that fortify the plant’s defense system against stress.

### GSNO-HAs-Fe-HAP NPs mitigate Cd stress via SNO-PCS interaction

In heavy metal (HM) contaminated soils, metals with a density greater than 5 g/cm³ can accumulate in plants, leading to toxicity. In response, plants produce phytochelatins (PCs) from glutathione (GSH) via the action of phytochelatin synthase (PCS).

Quantitative measurements indicated significantly higher accumulation of GSH in the leaf, stem, and root tissues following GSNO-HAs-Fe-HAP NP application. However, GSH quantities were drastically reduced in the presence of cPTIO. (Supplementary Fig. 8A–C). On the other hand, oxidized glutathione (GSSG) contents in all three types of plant tissues remained statistically similar after NP application with and without cPTIO (Supplementary Fig. 8D–F). A significantly higher GSH/GSSG ratio was recorded in response to Cd+ NP and NP application alone (Supplementary Fig. 8G–I). A high GSH:GSSG ratio indicates a reducing environment, which is essential for maintaining protein thiols in their reduced (–SH) state, protecting against oxidative damage and supporting enzyme function.

GSH-PC-HM interaction results in the formation of stable PC-HM complexes, which are transported to vacuoles by the ABCC transporters, thereby preventing HM ions from interfering with metabolism. On the other hand, S-nitrosothiols regulate NO signaling in plants under abiotic stresses, including HM stress^38,39^. Literature describes a complex interaction between NO and PCs in regulating plant responses to heavy metal stress^40,41^ (see discussion section for details).

**Fig. 7:**
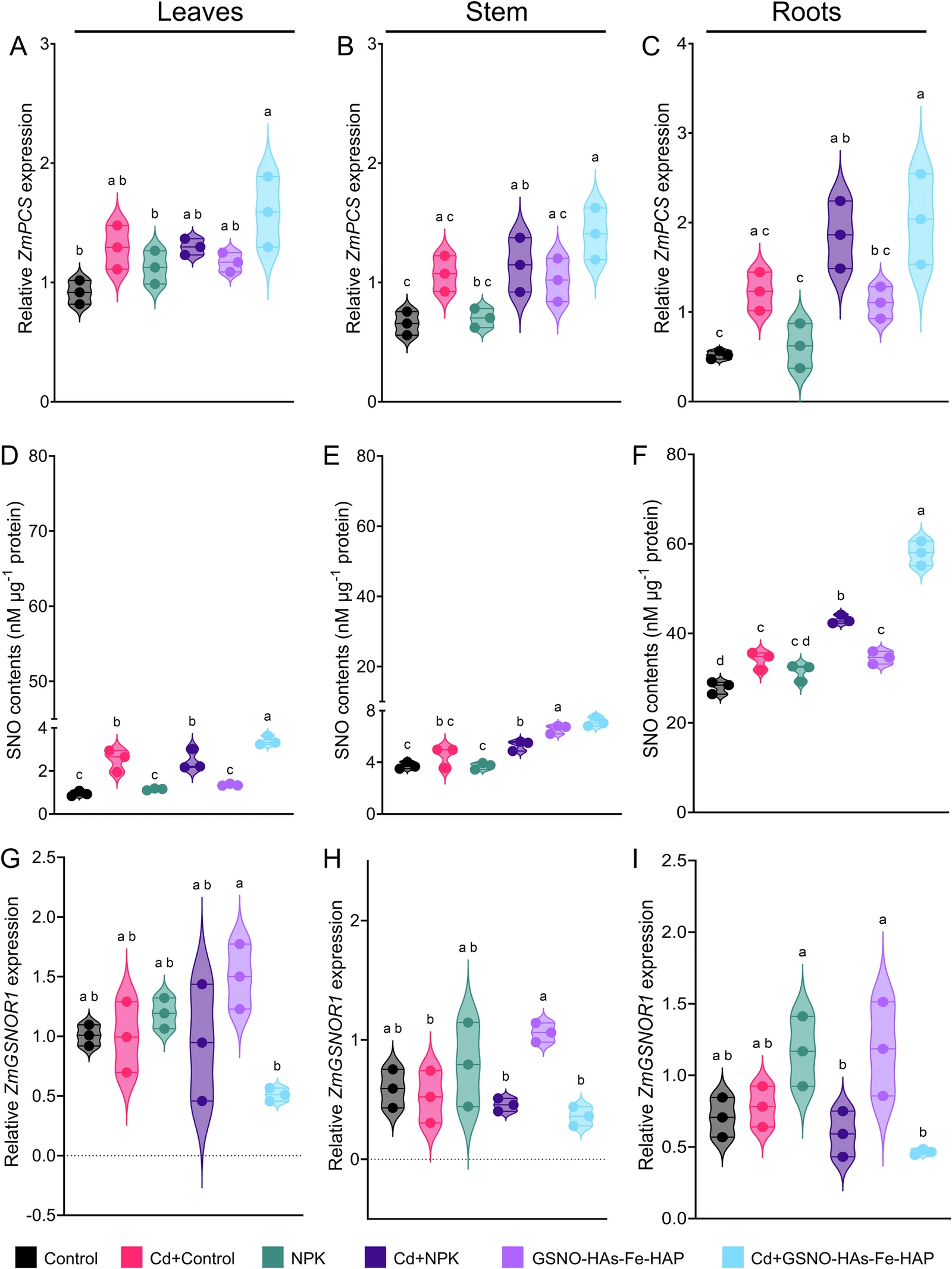
GSNO-HAs-Fe-HAP NPs mitigate Cd stress by mediating SNO-PCS interaction. Effects of GSNO-HAs-Fe-HAP NPs on phytochelatin (PC) synthesis and global S-nitrosothiol (SNO) accumulation were determined by measuring the relative expression of *ZmPCs* (**A–C**), total SNO content (**D–F**), and the relative expression of *ZmGSNOR1* (**G–I**) in the leaves, stems, and roots of maize seedlings exposed to Cd toxicity for 2 weeks. Data are presented as mean ± standard error of three independent biological replicates per treatment (*n* = 3). Each biological replicate corresponded to one independently grown and treated maize plant maintained in a separate pot. Tissue from each plant was collected, processed, and analyzed separately, and samples from different plants were not pooled. Data in (**A–I**) were analyzed using ordinary one-way ANOVA followed by two-sided Tukey’s all-pairs comparisons. *P* values were adjusted for multiple comparisons using Tukey’s method. Different lowercase letters indicate statistically significant differences at Tukey-adjusted *P* < 0.05. Omnibus ANOVA *P* values were: **A** *P* = 0.0068; **B** *P* = 0.0012; **C** *P* = 0.0002; **D** *P* = 2.007134 × 10^−6^; **E** *P* = 1.832857 × 10^−6^; **F** *P* = 1.600690 × 10^−9^; **G** *P* = 0.0164; **H** *P* = 0.0071; and **I** *P* = 0.0030.

Quantitative real-time PCR analysis showed that Cd toxicity elevated *ZmPCS* expression and SNO content in the leaves, stems, and roots of maize seedlings (Fig. 7A–C). Although NPK-treated leaves, stem, and roots displayed elevated *ZmPCS* expression and SNO levels (Fig. 7D–F), the increase was not drastically higher than that of the control plants. In contrast, the GSNO-HAs-Fe-HAP NPs-treated seedlings exhibited a significant enhancement in both *ZmPCS* expression and SNO content across all tissues compared to the Cd control. Interestingly, the global SNO content in the roots was nearly 20 times higher than in the leaves and 10 times higher than in the stem (Fig. 7D–F), as roots are the first point of contact with the impending Cd stress. Among the treatments, Cd+GSNO-HAs-Fe-HAP NPs yielded the highest SNO levels across all tissues, with increases of 37.9% in the leaves, 33.7% in the stem, and 34.6% in the root, relative to Cd+NPK.

These findings suggest that NO plays a crucial role in regulating the expression of *PCS* to promote Cd tolerance in maize seedlings treated with GSNO-HAs-Fe-HAP NPs. In light of these findings, one would assume a concomitant reduction in SNO turnover by the GSNOR enzyme via denitrosylation. Real-time PCR analysis indicated a marked decrease in the expression of *ZmGSNOR1* in GSNO-HAs-Fe-HAP NPs-treated seedlings under Cd stress (Fig. 7G–I), which may facilitate SNO accumulation, thus improving Cd tolerance via transcriptional regulation of PCS.

Interestingly, the application of GSNO-HAs-Fe-HAP NPs in combination with the NO scavenger cPTIO did not result in a significant change in the expression of *ZmPCS* in all three types of tissues (leaves, stem, and roots) (Supplementary Fig. 9A–C), and the expression patterns remained the same as described above. Similarly, the total S-nitrosothiol contents in the leaves and roots (Supplementary Fig. 9D, F) showed similar patterns to those in Fig. 7. However, SNO contents in the stem increased significantly following cPTIO application in combination with NPs (Supplementary Fig. 9E). On the other hand, cPTIO did not induce statistically significant changes in *ZmGSNOR* expression (Supplementary Fig. 9G–I), which followed an almost similar pattern as described in Fig. 7G–I.

The interaction between NO and maize PCs was further supported by the in silico prediction of potential S-nitrosylation targets in ZmPC. Results showed three cysteine residues in ZmPCS—Cys115, Cys354, and Cys418—with scores above the medium threshold (2.443) (Supplementary Fig. 10). FreeSASA-based solvent-accessible surface area calculations of the three cysteine residues exhibit high side-chain SASA values, indicating thiol exposure and a significantly higher chance for NO-mediated modification.

### GSNO-HAs-Fe-HAP: phytohormonal & transcriptional regulation

**Fig. 8:**
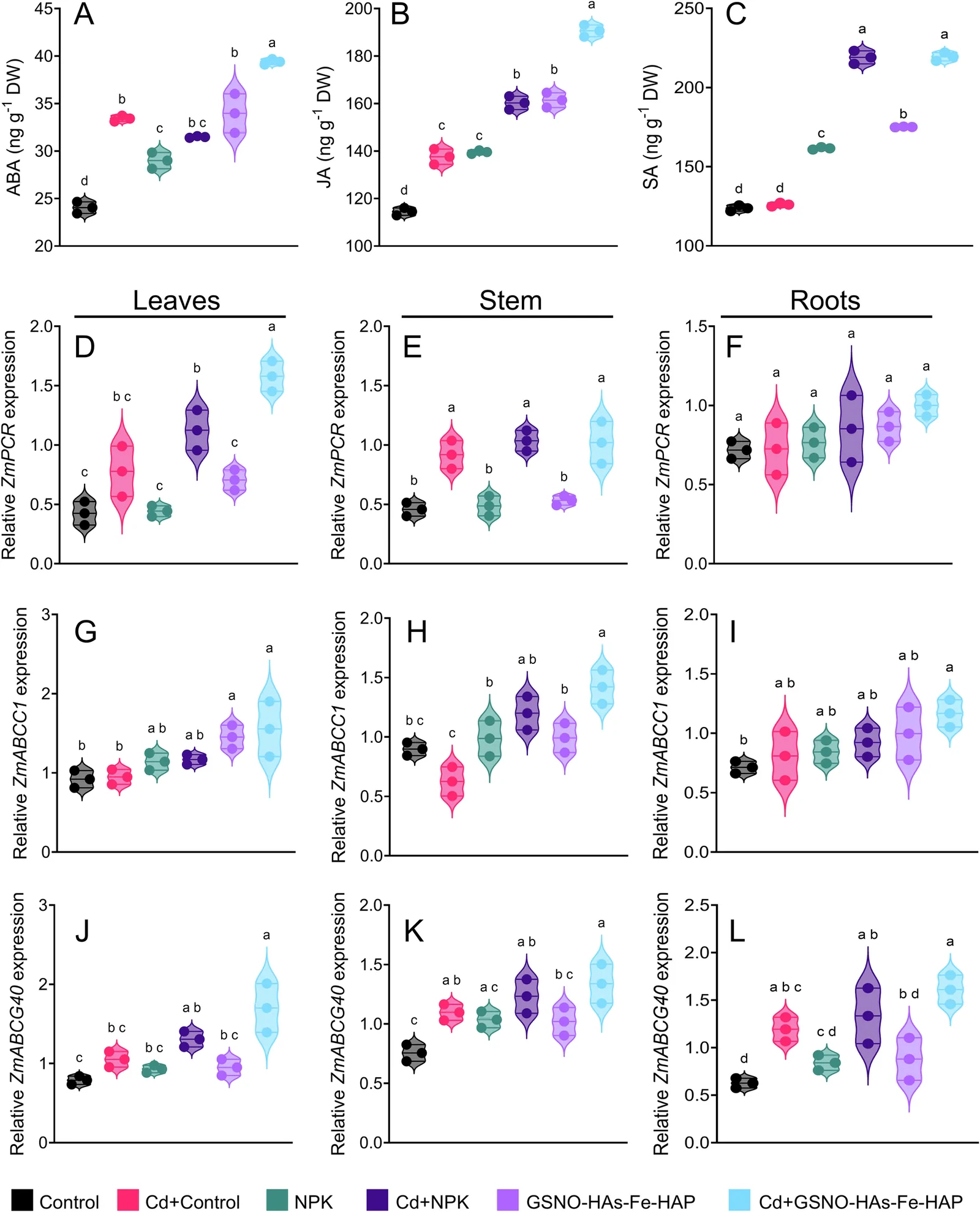
GSNO-HAs-Fe-HAP NPs protect plants via phytohormonal and transcriptional regulation. Effects of GSNO-HAs-Fe-HAP NPs on phytohormone levels and related transcripts of maize seedlings exposed to Cd toxicity for 2 weeks were determined by quantifying Abscisic acid (**A**), Jasmonic acid (**B**), and Salicylic acid (**C**) contents. Furthermore, effects on the transcription of key heavy metal transport genes were determined by quantifying changes in the expression of *ZmPCR* (**D–F**), *ZmABCC1* (**G–I**), and *ZmABCG40* (**J–L**) in the leaf, stem, and root tissues of plants under Cd stress. Data are presented as the mean ± standard error of three independent biological replicates per treatment (*n* = 3). Each biological replicate corresponded to one independently grown and treated maize plant maintained in a separate pot. Tissue from each plant was collected, processed, and analyzed separately, and samples from different plants were not pooled. Data (**A–L**) were analyzed using ordinary one-way ANOVA followed by two-sided Tukey’s all-pairs comparisons. *P* values were adjusted for multiple comparisons using Tukey’s method. Different lowercase letters indicate statistically significant differences at Tukey-adjusted *P* < 0.05. Omnibus ANOVA *P* values were: **A** *P* = 5.270317 × 10^−9^; **B** *P* = 2.081141 × 10^−12^; **C** *P* = 1.430105 × 10^−15^; **D** *P* < 0.0001; **E** *P* < 0.0001; **F** *P* = 0.1285; **G** *P* = 0.0033; **H** *P* = 0.0001; **I** *P* = 0.0348; **J** *P* < 0.0001; **K** *P* = 0.0007; and **L** *P* = 0.0002. *P* values reported as *P* < 0.0001 reflect the precision available in the exported statistical results.

GC-MS analyses recorded significantly elevated levels of abscisic acid (ABA), jasmonic acid (JA), and salicylic acid (SA) in maize seedlings under Cd stress, regardless of NPK and GSNO-HAs-Fe-HAP NPs supplementation (Fig. 8). Notably, under Cd stress, treatment with GSNO-HAs-Fe-HAP NPs led to a 25.1% increase in ABA (Fig. 8A) and an 18.9% increase in JA contents (Fig. 8B) compared to Cd-stressed plants treated with the commercial NPK fertilizer. Furthermore, a maximum of 39.4 ng ABA per gram of dry tissue was recorded in plants treated with the NP composite only, compared to 190 ng JA (Fig. 8B) and 219 ng SA (Fig. 8C) under the same conditions.

Among the treatments, GSNO-HAs-Fe-HAP NPs significantly increased both JA and SA under control conditions as well as under Cd stress (Fig. 8B, C). Parallel to the phytohormonal responses, the expression of key genes involved in Cd detoxification was also evaluated using real-time PCR. These included *PLANT CADMIUM RESISTANCE* (*ZmPCR*), *ATP-BINDING CASSETTE TRANSPORTER C1* (*ZmABCC1*), *ATP-BINDING CASSETTE TRANSPORTER G40* (*ZmABCG40*), *HEAVY METAL ATPASE 4* (*HMA4*), *HMA9*, and *NATURAL RESISTANCE-ASSOCIATED MACROPHAGE PROTEIN 5* (*ZmNramp5*).

*ZmPCR* plays a pivotal role in regulating Cd accumulation and transport in plants, thereby reducing toxicity and enhancing stress tolerance^42,43^. In the present study, under Cd stress, the expression level of *ZmPCR* was significantly elevated in the leaves and stem of Cd-stressed plants (Fig. 8D, E), with the GSNO-HAs-Fe-HAP NP-treated plants showing increases of 123.9% and 91.5%, respectively, compared to GSNO-HAS-Fe-HAP-treated plants. However, no statistical differences were observed in *ZmPCR* expression in the roots (Fig. 8F). This tissue-specific response may help mitigate oxidative damage and preserve photosynthetic efficiency under Cd stress.

Significant differences were also observed in the expression levels of ATP-binding cassette transporter genes, *ZmABCC1* and *ZmABCG40*, that facilitate the vacuolar sequestration of PC-metal complexes, enhancing heavy metal detoxification and tolerance^44,45^. Under Cd stress, GSNO-HAs-Fe-HAP NPs enhanced *ZmABCC1* (Fig. 8G–I) and *ZmABCG40* (Fig. 8J–L) expression in leaves, stem, and roots, highlighting the potential of the nanoparticle composite in improving Cd detoxification.

Interestingly, the application of GSNO-HAs-Fe-HAP NPs in combination with the NO scavenger cPTIO showed relatively less reduction in the expression of *ZmPCR* in the leaves, stem, and root tissues, as compared to GSNO-HAs-Fe-HAP NPs only (Supplementary Fig. 11A–C). However, significantly greater differences in the expression of *ZmABCC1* were observed in tissues treated with NPs with and without cPTIO (Supplementary Fig. 11D–F), whereas the expression pattern of *ZmBCG40* remained the same as described above.

Additionally, changes in the expression of *ZmHMA4* and *ZmHMA9*, involved in metal ion translocation^46^, were also investigated under Cd stress (Supplementary Fig. 12). QPCR analysis of leaf, stem, and root samples indicated a modest increase in the expression of *ZmHMA4* (Supplementary Fig. 12A–C), though the increase was not statistically significant. On the other hand, the expression of *ZmHMA9* in the Cd+GSNO-HAs-Fe-HAP-treated plants increased by 88.8% in the leaves (Supplementary Fig. 12D), 70.9% in the stem (Supplementary Fig. 12E), and 45.6% in the roots (Supplementary Fig. 12F) compared to plants treated with GSNO-HAs-Fe-HAP NPs alone. In contrast, with Cd+NPK treatment, *ZmHMA9* expression increased by 86.1% in the leaves compared to NPK alone, but no significant differences were observed in the stem and root tissues.

*ZmNramp5*, a key Cd influx transporter, is upregulated under Cd stress, and loss-of-function *nramp5* rice plants show poor growth and yield^47^. Surprisingly, in the present study, we observed no significant differences in *ZmNramp5* expression across all treatment conditions (Supplementary Fig. 12G‒I), with expression even lower in the NPK and GSNO-HAs-Fe-HAP NPs treatments. Overall, these results indicate that the GSNO-HAs-Fe-HAP nanoparticle composite synthesized in this study effectively modulates the expression of metal-associated genes, enhancing maize seedling resilience to Cd toxicity.

### GSNO-HAs-Fe-HAP NPs enhance soil health under Cd stress

**Fig. 9:**
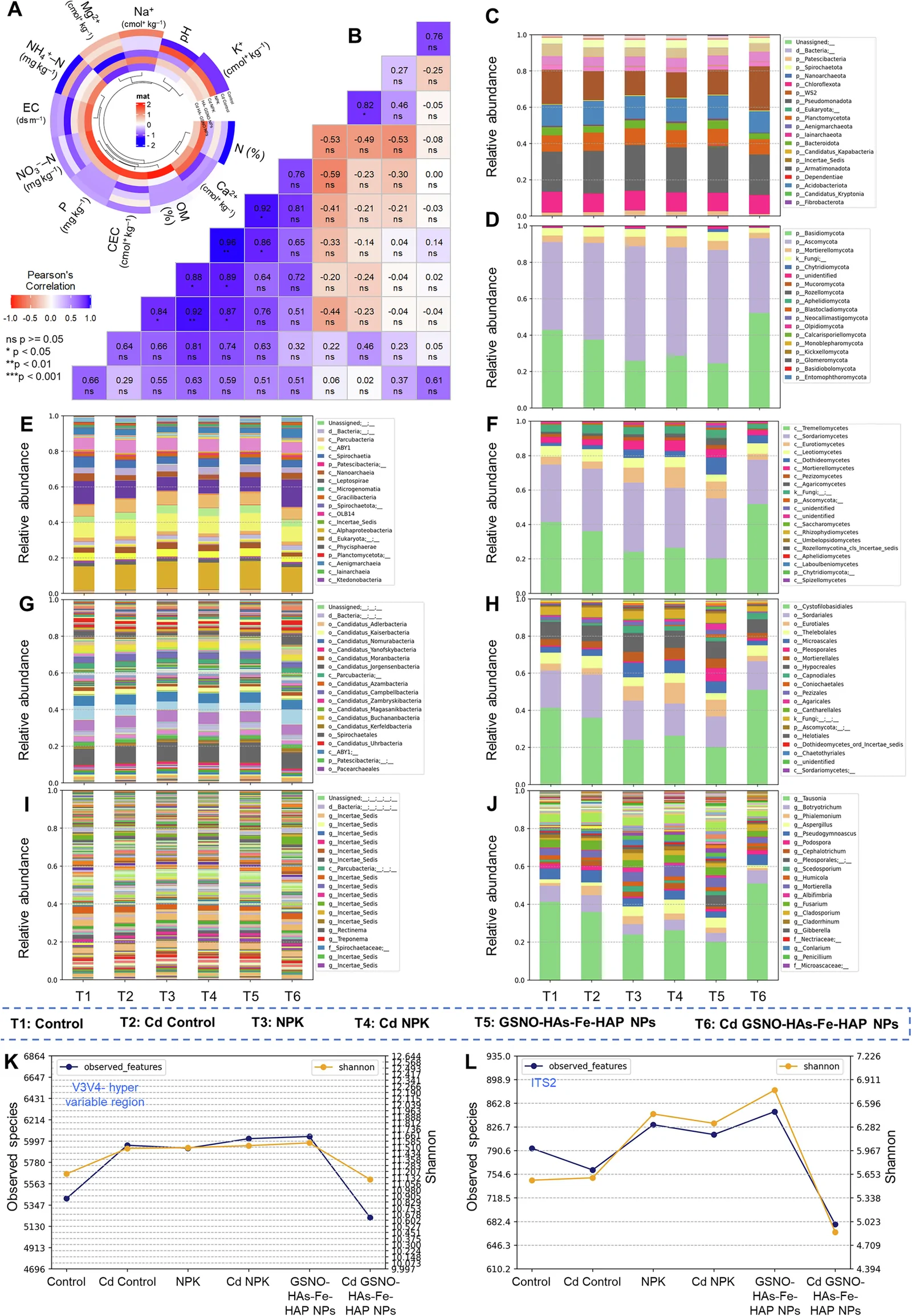
GSNO-HAs-Fe-HAP NPs improve soil health and protect the soil microbiome. Cd contamination impaired soil health. GSNO-HAs-Fe-HAP NPs improved soil health by improving crop nutrients and stimulants (**A**), including organic matter (OM), cation exchange capacity (CEC), available phosphorus, nitrate (NO_3_^‒^), and ammonium (NH_4_^+^) nitrogen. Pearson’s correlation revealed a significantly positive relationship among these enhanced soil properties (**B**). Taxonomic profiling revealed enhanced bacterial and fungal stability and remodeling following GSNO-HAs-Fe-HAP NPs treatment under normal and Cd stress. Relative abundance of bacterial **C**, **E**, **G, I**) and fungal (**D**, **F**, **H, J**) communities was profiled at the phylum (**C**, **D**), class (**E**, **F**), order (**G**, **H**), and genus (**I**, **J**) levels across six experimental groups (T1–T6). The bacterial community structure (**C**, **E**, **G, I**) exhibited enhanced stability across the treatments and was dominated by the core phyla *Pseudomonadota*, *Chloroflexota*, and *Acidobacteriota*. Although these communities appeared to persist regardless of Cd exposure or fertilizer type, the relative abundance of certain phyla was significantly higher than others following NP application. In contrast, the fungal communities (**D**, **F**, **H, J**) displayed a treatment-specific configuration. Under Cd stress, GSNO-HAs-Fe-HAP NPs treatment induced a dramatic bloom of the *Asomycota* and *Basidiomycota* phyla (**D**), driven sequentially by the class *Tremellomycetes* and *Sordariomycetes* (**F**), order *Cystofilobasidiales* and *Sordariales* (**H**), and exclusively by the yeast genus *Tausonia* (**J**). Notably, *Tausonia* occupied >50% of the fungal community in the Cd GSNO-HAs-Fe-HAP NPs group (T6), a phenomenon absent in the Cd-NPK group (T4). Alpha diversity indices indicated microbial dominance induced by GSNO-HAs-Fe-HAP NPs. Line plots representing alpha diversity metrics, including the observed species (richness, blue line) and Shannon index (evenness, orange line), for bacterial (**K**) and fungal (**L**) communities. The bacterial diversity (**K**) remained consistently high (Observed species > 5300; Shannon > 10.5), indicating that the resilience of the indigenous bacteriome is preserved under GSNO-HAs-Fe-HAP NPs application. Fungal diversity (**L**) exhibited a precipitous decline, specifically in the Cd GSNO-HAs-Fe-HAP NPs group. The sharp reduction in the Shannon index (Shannon <5.0) reflects the ecological dominance of the enriched genus *Tausonia*, which decreased overall community evenness.

Cd contamination impairs soil health and reduces crop productivity. Given that the effects of NPs on soil quality are often overlooked, this study examined the potential of GSNO-HAs-Fe-HAP NPs in improving soil properties in Cd-contaminated environments by co-releasing crop nutrients and stimulants, as observed in the in vitro assay (Fig. 9). Compared to Hoagland and the commercial NPK fertilizer treatments, GSNO-HAs-Fe-HAP NP treatment markedly increased organic matter (OM), cation exchange capacity (CEC), available phosphorus, nitrate nitrogen (NO_3_^‒^), and ammonium nitrogen (NH_4_^+^-N), regardless of Cd contamination (Fig. 9A). Pearson’s correlation analysis further revealed a significantly positive relationship among these enhanced soil properties (Fig. 9B). Although soil sodium, potassium, and magnesium levels showed no significant differences across the treatments, GSNO-HAs-Fe-HAP NPs notably increased soil nitrogen and calcium levels (Fig. 9A). These findings indicate that GSNO-HAs-Fe-HAP NPs effectively mitigate the adverse effects of Cd contamination, a benefit not seen with commercial fertilizers. On the other hand, GSNO-HAs-Fe-HAP NPs application significantly reduced the soil Cd content when measured after the termination of the experiments, besides improving soil P, K, and Fe contents (Supplementary Fig. 13).

Previous studies have highlighted the individual benefits of HAPs and HAs in improving soil health and nutrient retention, particularly in contaminated soils^48–51^. Building on these findings, the integration of HAPs and HAs with GSNO in the GSNO-HAs-Fe-HAP NPs designed as a potential smart fertilizer proved highly effective in increasing nutrient mobility, retention, and water-holding capacity, while also optimizing soil nitrogen dynamics. Collectively, these improvements contribute to the preservation and enhancement of overall soil health.

### Effect on soil microbiome under Cd stress

To elucidate the impact of GSNO-HAs-Fe-HAP NPs on maize rhizospheric microbiome under cadmium (Cd) stress, we profiled bacterial (16S rRNA V3-V4) and fungal (ITS2) community dynamics across six experimental groups (Fig. 9 and Supplementary Figs. 14–16). The soil bacterial community exhibited remarkable structural resilience and phylogenetic conservation under Cd stress as the relative abundance of core phyla (e.g., Pseudomonadota, Chloroflexota) remained stable (Fig. 9C, E, G, I). Beta diversity analyses revealed a stochastic distribution of samples, confirming that the nanoparticles act as an ecologically biocompatible amendment that preserves functional homeostasis. In sharp contrast, the fungal microbiome underwent a profound structural reconfiguration (Fig. 9D, F, H, J) specifically in response to the combined stimuli of Cd stress and GSNO-HAs-Fe-HAP NPs, characterized by a dramatic expansion of the phylum Basidiomycota ( > 50% abundance) (Fig. 9D) driven exclusively by a bloom of the genus Tausonia (identified as *T. pullulans* at the species level (Supplementary Fig. 14N). This specific proliferation resulted in a precipitous drop in alpha diversity in response to NP application under Cd stress (Fig. 9L), signifying a strategic transition to a community dominated by a specialized, stress-adapted lineage. Decoupled beta diversity metrics further elucidated the drivers of this shift: the substantial variance explained by Bray-Curtis (58.42%) compared to Jaccard (24.74%) (Supplementary Figs. 14–16), confirming that the shift was driven by the disproportionate proliferation of resident taxa rather than species turnover. In comparison, the high Weighted UniFrac variance (75.78%) (Supplementary Fig. 16) indicates that the NPs treatment selectively amplified a phylogenetically distinct evolutionary lineage to cope with Cd stress.

### GSNO-HAs-Fe-HAP NPs: Effect on Cd and yield

**Fig. 10:**
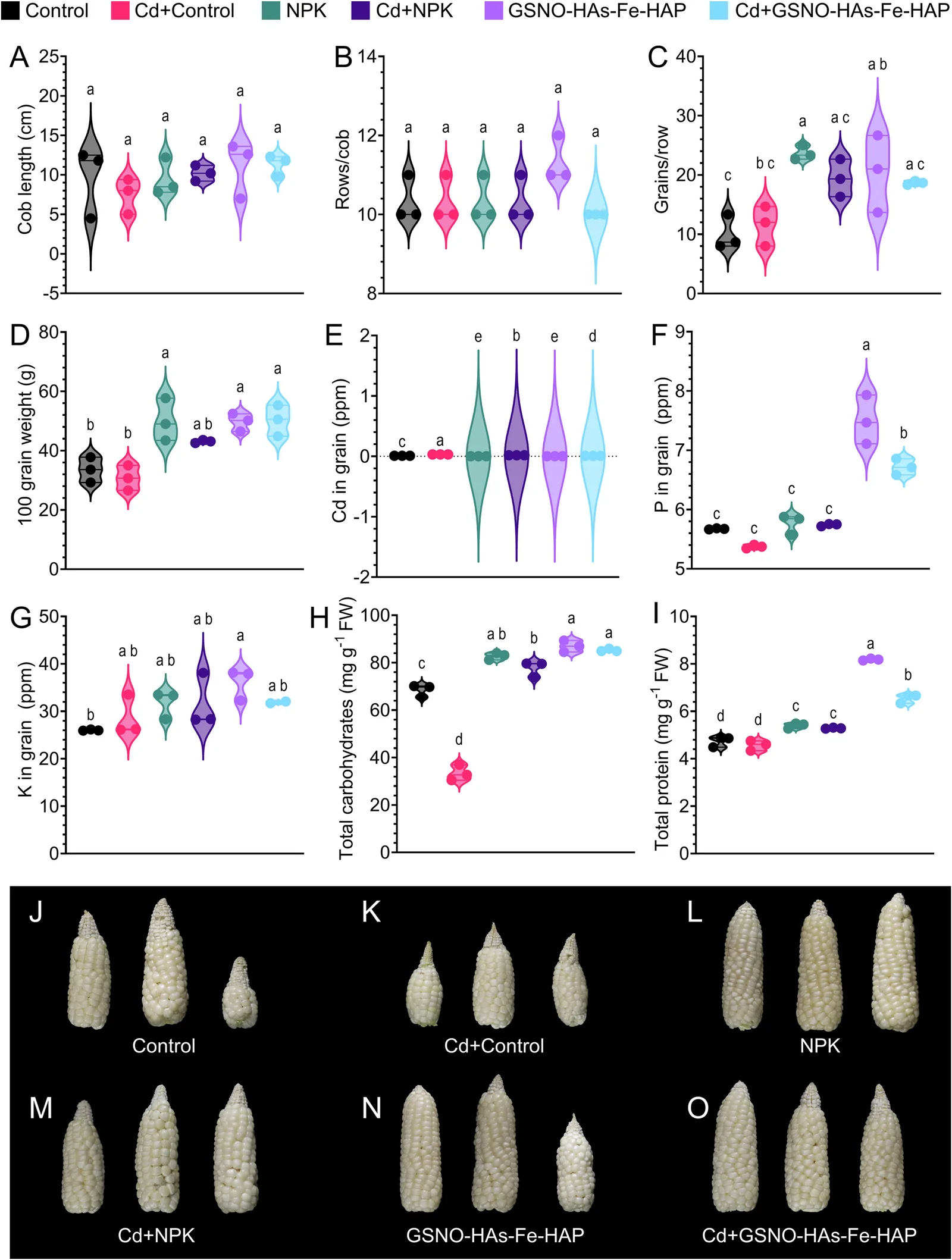
GSNO-HAs-Fe-HAP NPs reduce cadmium accumulation and enhance yield and preserve nutritional quality in maize grains. Effects of GSNO-HAs-Fe-HAP NPs on grain yield and Cd accumulation were determined after harvest. Data on parameters yield related parameters, including cob length (**A**), rows/cob (**B**), grains/row (**C**), 100 grain weight (**D**), Cd in grain (**E**), phosphorus (**F**), and potassium (**G**) contents, total carbohydrate (**H**), and protein (**I**) contents were recorded. Panels **J-O** show the corncobs from plants subjected to various treatments. Data are presented as the mean ± standard deviation of three independent biological replicates per treatment (*n* = 3). Each biological replicate consisted of one independently grown and treated maize plant, and samples from different plants were not pooled. For each parameter in (**A–I**), three technical measurements were obtained from each plant and averaged to generate a single value per biological replicate for statistical analysis; technical replicates were not treated as independent observations. Data in (**A–I**) were analyzed using ordinary one-way ANOVA followed by two-sided Tukey’s all-pairs comparisons. *P* values were adjusted for multiple comparisons using Tukey’s method. Different lowercase letters indicate statistically significant differences at Tukey-adjusted *P* < 0.05. Omnibus ANOVA *P* values were: **A** *P* = 0.5990; B *P* = 0.1172; **C** *P* = 0.0028; **D** *P* = 0.0004; **E** *P* < 0.0001; **F** *P* < 0.0001; **G** *P* < 0.0001; **H** *P* < 0.0001; and **I** *P* < 0.0001. *P* values reported as *P* < 0.0001 reflect the precision available in the exported statistical results.

Cadmium is a toxic heavy metal, and its accumulation in edible grains can lead to severe health issues. To evaluate the impact of GSNO-HAs-Fe-HAP NPs on the Cd accumulation and nutritional value in maize grains, we quantified the grain Cd content, as well as key yield and nutritional parameters, at the harvest stage (Fig. 10). As shown in Fig. 10, Cd treatment alone caused a substantial reduction in various yield parameters, including cob length, number of grains, and grain weight (Fig. 10A–D). On the other hand, the NP-treated plants showed significantly enhanced yield-related parameters under both normal and Cd stress conditions. Cd stress (Cd+Control) caused a substantial accumulation of Cd in grains ( ~ 0.032 ppm), significantly higher than in the non-stressed control, indicating unrestricted translocation of the heavy metal to reproductive tissues. While conventional NPK fertilization offered partial mitigation, the GSNO-HAs-Fe-HAP NP treatment demonstrated superior efficacy, significantly reducing grain Cd content (0.006 ppm), levels comparable to the non-stressed control (Fig. 10E). In parallel, the essential mineral content in grains was markedly improved by the nanocomposite; P and K levels in the Cd+GSNO-HAs-Fe-HAP group were significantly higher than those in the Cd+NPK group (Fig. 10F, G), with P content reaching levels statistically equivalent to the GSNO-HAs-Fe-HAP group. Furthermore, Cd stress severely compromised macromolecule synthesis, evidenced by the lowest total carbohydrates (Fig. 10H) and protein (Fig. 10I) contents in the Cd+Control group. However, supplementation with GSNO-HAs-Fe-HAP NPs under Cd stress restored total protein content and significantly outperformed the Cd+NPK group. Similarly, total carbohydrate content in the Cd+GSNO-HAs-Fe-HAP group was restored to levels statistically on par with the NPK-only treatment and drastically higher than the Cd+Control. Collectively, these results demonstrate that GSNO-HAs-Fe-HAP NPs not only effectively restrict Cd entry into the edible parts of the crop but also actively preserve grain nutritional quality by maintaining protein and carbohydrate synthesis under heavy metal stress.

In addition, the long-term impact of NP composite under Cd stress was determined by analyzing its effects on key growth metrics compared with control plants or those treated with commercial fertilizers, both in the absence and presence of Cd stress. These include Leaf number, plant height, stem length/diameter (Supplementary Fig. 17A–D), and net photosynthetic rate, stomatal conductance, transpiration rate, and intercellular CO_2,_ each measured for up to 98 days (Supplementary Fig. 17E–H).

## Discussion

Agriculture in heavy-metal-contaminated soils is a great challenge. Crop plants must either avoid or reduce the absorption of heavy metal ions, strictly regulate the influx and efflux of heavy metals (HM) in cells, or activate a cascade of defense networks to maintain growth and resist heavy metal stress. However, most of the time, crops succumb when exposed to a plethora of other biotic and abiotic stresses. Active defense against stress is an energy-exhaustive process resulting in a defense-yield tradeoff or yield penalty^52^. Having crop varieties genetically resistant to all these stresses simultaneously would be a dream come true. Hence, it is important to devise strategies that not only mitigate heavy metal stress but also enhance plant growth and development at the same time. Nanoparticles offer transformative solutions for sustainable agriculture in heavy metal-contaminated soils. Their physicochemical properties, especially the smaller size, high surface area, reactivity, and tunable functionality, enable them to mitigate HM toxicity, enhance crop resilience, and improve soil health.

The present study reports the development and evaluation of a GSNO-HAs-Fe-HAP nanoparticle composite as a multifunctional nanofertilizer capable of improving plant tolerance to cadmium (Cd) toxicity. Extensive investigations showed that these nanoparticles simultaneously deliver nitric oxide (NO), humic substances, and essential nutrients, while promoting antioxidant defense, osmolyte accumulation, expression of metal detoxification genes, and enhancing agronomic traits in maize seedlings under Cd stress. Our synthesis strategy employed a one-pot method that ensured the stable incorporation of S-nitrosoglutathione (GSNO) and humic acids (HAs) into a hydroxyapatite (HAP) matrix, facilitated by ferric-ion crosslinking.

After synthesis, the nanoparticles appeared to have a distinct physical appearance, with HAP and GSNO-HAP particles appearing white. In contrast, GSNO-HAs-HAP and GSNO-HAs-Fe-HAP were brown and dark brown, respectively. The dark color of the particles suggests successful integration of humic substances^11,13^. Thermogravimetric analysis indicated only 5% reduction in the mass of hydroxyapatite NPs at 1000 ^o^C. On the other hand, about 25% mass reduction in GSNO-HAs-Fe-HAP NPs at 1000 ^o^C, likely due to the organic coating layers of GSNO and HAs, was recorded.

Spontaneous, non-stop release of nutrients is a key drawback of most commercial fertilizers, resulting in nutrient leaching and water table contamination. On the other hand, fertilizer response to root exudates and the rhizosphere microclimate are highly desirable properties. Compared to deionized water or HCl, the GSNO-HAs-Fe-HAP NP composite synthesized in this study was found to be more responsive to citric acid (which is representative of organic acids commonly found in root exudates) by releasing significantly higher quantities of soluble nutrients and humic substances. In a different study involving hydroxyapatite, Yoon et al.^13^ described that organic acids disrupt non-covalent interactions within MPN-based supramolecular assemblies, facilitating time-dependent co-release of nutrients and stimulants from modified HAP cores.

Since NO is a key molecule to be delivered by the GSNO-HAs-Fe-HAP NPs, and its slow release over a longer duration is important^53^, we measured its release over time. Results showed sustained NO release for over 148 hours, compared to the rapid depletion seen in the canonical NO donor GSNO. This prolonged release may be associated with interactions of GSNO with ferric ions and HAs on the HAP surface, which could retard its release and decomposition. As HAP gradually dissolves under acidic conditions, the gradual release and subsequent decomposition of GSNO may contribute to sustained NO release^54^. Another possible mechanism may involve the redox activity of HAs. Previous studies have shown that humic acid possess redox-active functional groups and can participate in electron-transfer reactions. Thus, co-released HAs may potentially faciliatate GSNO decomposition and subsequent NO release^14,55,56^. Furthermore, together the UV-Vis and SEC data suggested that a portion of HAs may detach in aqueous conditions to stimulate plant growth, while the remaining fraction participates in GSNO reduction. The improved thermal and chemical stability, coupled with acid-triggered disassembly and nutrient release, and prolonged NO delivery, confirms the suitability of this nanofertilizer for soil applications.

A number of methods for detecting and measuring nitric oxide in biological samples have been described so far, each with distinct advantages and trade-offs. These include fluorescence, electrochemical sensors, Griess assay, electron paramagnetic resonance (EPR), and chemiluminescence-based detection.

Among these, the chemiluminescence-based NO measurement using Siever’s Nitric Oxide Analyzer (Siever’s NOA) is one of the most sophisticated methods for NO measurements with a detection sensitivity of ~1 picomole (pM). It’s the method of choice for absolute quantification and a standard of accuracy, ideal for measuring gaseous NO and conducting real-time kinetic studies of NO release.

Depending on the NO donor under study, NO is released spontaneously, via photoreduction, or under a mildly acidic environment. In this study, NO detection started immediately (Fig. 2G), i.e., at zero time point. There could be several plausible mechanistic explanations for such instant release of NO from GSNO-HAs-Fe-HAP NPs. For example, GSNO is also known to release NO spontaneously. Furthermore, we performed the measurements in a simple, mildly acidic aqueous solution, using 1 M citric acid to adjust the pH of the aqueous suspension containing the NP composite to 4.5. We chose this condition to test the intrinsic properties of our nanocomposite and to mimic the acidic microenvironment that may develop in the rhizosphere due to the secretion of organic acids from plant roots. As stated above, Ferric ions and electron-donating groups in humic acid have a critical role in GSNO immobilization. Hence, HAs may also mediate GSNO reduction, thereby contributing to instant NO release.

The prolonged release may first be attributed to the gradual liberation of structurally stabilized GSNO from the surface or matrix of the composite. GSNO may remain relatively stable while physically associated with humic acids and coordinated with Fe ions within the particles. However, once released during particle dissolution, GSNO may undergo decomposition or transformation, thereby generating NO. Second, the reducing capacity of the co-released humic acids may promote the transformation of GSNO. The humic acids used in this study contained phenolic functional groups that can be oxidized to quinone-like structures, and phenol–quinone redox cycling in humic substances is widely recognized as an important mediator of environmental redox reactions. Accordingly, reductive reactions mediated by the co-released humic acids may facilitate GSNO decomposition and contribute to sustained NO generation. In fact, redox reactions can facilitate NO release from GSNO^57^.

FITC-labeled confocal microscopy showed significant accumulation of the GSNO-HAs-Fe-HAP in plant tissues. Salam et al.^58^ reported significant accumulation of ZnO NPs in the aleurone layer and embryo of seeds following priming, thereby enhancing cobalt stress tolerance. Cd typically accumulates in plant roots, stem, and leaves through both apoplastic and symplastic pathways^59^, often degrading and disrupting these transport systems. However, GSNO-HAs-Fe-HAP NPs appear to maintain tissue integrity and function, likely by mitigating excessive Cd uptake, thereby bolstering the plant’s resilience to HM toxicity.

Functionally, the application of GSNO-HAs-Fe-HAP NPs significantly mitigated Cd-induced growth inhibition in maize. Compared to Cd-stressed controls, GSNO-HAs-Fe-HAP NPs -treated plants showed increased shoot and root biomass, chlorophyll retention, and improved photosynthetic pigment concentrations. Since we advocate that the NP composite synthesized in this study is much superior to conventional commercial fertilizer, we included commercial NPK fertilizer in all our experiments and compared the data. In all the investigations, the performance of the NP composite was superior to that of the commercial NPK fertilizer, especially under Cd stress, indicating that the GSNO-HAs-Fe-HAP NPs induce a synergistic effect of nutrition and stress tolerance. These positive effects are attributed to the slow, controlled release of nutrients, humic substances, and NO, which has been previously shown to support metal detoxification and enhance photosynthetic efficiency^1,60^. Furthermore, the composite also releases nutrients and humic acids into the soil profile, thereby positively affecting plant growth. Thus, GSNO-HAs-Fe-HAP NPs offer a more effective and sustainable strategy for mitigating Cd toxicity and promoting plant health compared to conventional fertilizers.

Furthermore, the NP composite treatment resulted in 83% and 86% lower accumulation of the toxic Cd ions in maize leaves and stem, respectively. These findings align with previous reports indicating that HAP and HAs can effectively chelate Cd ions, thereby limiting their uptake^61–63^. Additionally, the role of NO in reducing Cd translocation is well established, as seen in treatments using NO donors like sodium nitroprusside^64,65^. Thus, the sustained release of NO from GSNO-HAs-Fe-HAP NPs appears to offer enhanced and prolonged protection against Cd-induced damage compared to conventional NO applications or NPK fertilizers. However, NO biology in plants is highly complex. An interesting study by Besson-Bard et al.^66^ demonstrated that NO production exacerbates Cd toxicity in Arabidopsis by promoting Cd accumulation in the roots via up-regulation of Cd/Fe transporter *IRT1* and promoting Cd uptake and reducing Ca^2+^ uptake. The findings of this study are not contradictory but rather highlight a critical distinction from Bensson-Bard et al. While uncontrolled endogenous NO may facilitate metal uptake, the controlled, exogenous delivery of NO and subsequent SNOs via our GSNO-HAs-Fe-HAP nanocomposite bypasses this pathological pathway, instead enhancing antioxidant defense and directly chelating Cd^2+^, thereby reversing the toxic cascade observed in the above study.

Biochemical analyses revealed that GSNO-HAs-Fe-HAP NP treatment activated the antioxidant defense system more effectively than NPK. Specifically, the activities of POD and APX were markedly elevated in leaf and stem tissues, suggesting enhanced detoxification of ROS. Although CAT activity showed only a modest increase in the leaf, this may reflect a tissue-specific regulation of H_2_O_2_ detoxification. Interestingly, SOD activity remained largely unchanged under GSNO-HAs-Fe-HAP NPs treatment, indicating that downstream peroxidases played a more dominant role in the ROS-scavenging cascade. Similar reductions in MDA levels have been reported with silicon and iron NPs in *Solanum lycopersicum*^67^ and *Solanum melongena*^68^, under Cd stress. Collectively, while HAP, HAs, and GSNO are individually recognized for mitigating oxidative damage in plants^60,61^, their combination in GSNO-HAs-Fe-HAP NPs appears to exert a synergistic protective effect by efficiently suppressing ROS formation and protecting against cellular damage

Proline accumulation, a key osmoprotectant response to heavy metal stress, was significantly higher in GSNO-HAs-Fe-HAP NPs -treated plants under cadmium stress, with increases of up to 196.7% in roots compared to Cd Controls. This correlated with elevated expression of *ZmP5CR1*, a gene central to proline biosynthesis. Increased proline accumulation helps stabilize proteins and membranes, maintain osmotic balance, and scavenge ROS, thereby supporting stress tolerance under adverse conditions^69–72^. Thus, the elevated proline levels observed in GSNO-HAs-Fe-HAP NP-treated seedlings suggest enhanced cellular protection and effective mitigation of Cd-induced oxidative damage^73^. Furthermore, the GSNO-HAs-Fe-HAP NPs boosted total carbohydrate and soluble sugar content in all tissues, supporting their role in maintaining energy metabolism and osmotic balance under stress^74^. Hence, the NP composite demonstrated a superior capacity to promote osmotic adjustment and confer enhanced Cd tolerance, reinforcing the potential of nanotechnology-based fertilizers.

At the molecular level, GSNO-HAs-Fe-HAP NPs treatment enhanced the expression of phytochelatin synthase (*ZmPCs*) and reduced glutathione-dependent nitric oxide reductase (*ZmGSNOR1*), facilitating the accumulation of S-nitrosothiols (SNOs), which are implicated in heavy metal detoxification through S-nitrosylation. The co-accumulation of SNOs and upregulation of *ZmPCs* suggest a role for S-nitrosylated phytochelatins in mediating Cd tolerance. Additionally, elevated expression of metal transporter genes (e.g., *ZmHMA4*, *ZmHMA9*, *ZmPCR*) in GSNO-HAs-Fe-HAP NPs -treated plants further indicates an active mobilization and compartmentalization of Cd, helping to limit its cytotoxic effects.

Metals with a density greater than 5 g/cm³ accumulate in plants, causing toxicity. In response, plant PCs bind to heavy metal ions, reducing toxicity. PCs are derived from the abundant tripeptide GSH. On the other hand, NO binds to GSH, forming GSNO. GSH-PC-HM interaction results in the formation of stable PC-HM complexes. Literature describes a fascinating but largely uninvestigated interaction between PCs and NO. PCs chelate and sequester metal ions via binding^75^. PCs were first characterized by Grill et al.^76^ in 1985, although their presence had been suggested earlier in mercury-treated tobacco leaves^77,78^. Salt et al.^79^ found that 26 pmol/g dry weight PCs in the roots of *Brassica juncea* were sufficient to bind most of the Cd detected in the roots. In *Arabidopsis*, *PHYTOCHELATIN SYNTHASE 1* (*PCS1*) and *PCS2* enhance resistance to Cd toxicity^80,81^. This response is further supported by Cd-induced increase in NO levels, which enhances tolerance by reducing Cd uptake, stimulating PC production, and alleviating oxidative damage^82,83^.

S-nitrosylation regulates NO signaling in plants under abiotic stresses, including HM stress^38,39,84^. This covalent modification adds a nitrosyl group to the reactive thiol of a cysteine residue, forming SNO, a byproduct of GSNO degradation by GSNO reductase (GSNOR)^85,86^. Given that PCs are crucial for Cd detoxification, the involvement of NO in PCs, discussed by De Michele, et al.^40^ sparked considerable interest. They describe that NO is vital for programmed cell death, and inhibiting NO synthesis can preserve cell viability under Cd stress. However, it remains unclear whether increased PC levels could delay cell death or enhance viability. They noted that both Cd^2+^ and NO can bind to the cysteine residues of PCs, potentially reducing the chelation effectiveness of nitrosylated PCs, which could allow free ions to exert toxicity. However, later, Elviri, et al.^41^ demonstrated that the stability of the NO group in PCs increases with the degree of polymerization, demonstrating greater stability in the cysteine-rich PC_4_ compared to PC_2_ via tandem mass spectrometry (MS/MS) experiments.

Under Cd stress, SNO content increased significantly, likely due to the gradual release and decomposition of GSNO from GSNO-HAs-Fe-HAP NPs, which provided a sustained source of NO and facilitated S-nitrosylation. This process is known to enhance heavy metal detoxification through the formation of S-nitrosylated phytochelatins (SNO-PCs). These findings suggest that S-nitrosylated phytochelatins may contribute to the improved Cd tolerance observed in maize seedlings treated with GSNO-HAs-Fe-HAP NPs. Furthermore, since GSNOR regulates NO bioavailability by catalyzing GSNO degradation, we observed a significant downregulation of *ZmGSNOR1* expression in the Cd GSNO-HAs-Fe-HAP NPs, with reductions of 65.9% in the leaf, 66.1% in the stem, and 60.8% in the root, compared to the Cd NPK. This suppression of *ZmGSNOR1* likely contributed to the accumulation of SNOs^87^, sustaining NO signaling and enhancing Cd detoxification under stress conditions.

In terms of secondary metabolism, GSNO-HAs-Fe-HAP NPs application restored phenylalanine ammonia lyase (PAL) activity and *ZmPAL* expression in leaves and stem, which were otherwise suppressed under Cd stress. These findings align with previous reports on the role of nanomaterials in modulating PAL activity and promoting plant resilience^88–90^. This was further accompanied by increases in total flavonoid and phenolic contents, reinforcing the importance of secondary metabolites in heavy metal stress tolerance. Moreover, phytohormonal analysis revealed that GSNO-HAs-Fe-HAP NPs selectively increased abscisic acid (ABA) and jasmonic acid (JA) levels—two key hormones in abiotic stress signaling—while salicylic acid (SA) remained unchanged, pointing to a specific hormonal modulation profile that favors detoxification and defense. Previous studies have highlighted the protective roles of these phytohormones under heavy metal stress. *Arabidopsis* ABA-deficient mutants *aba-1*, *aba-3*, and *aba-4*, or the ABA signaling mutants *abi2-1* and *abi3-1*, exhibit high sensitivity to Cd, underscoring ABA’s role in metal detoxification^91^. Similarly, exogenous ABA application has been shown to reduce Cd accumulation and improve growth parameters in grapevine and lettuce^92^. JA also contributes to metal tolerance, as demonstrated by increased Cd sensitivity in JA-deficient *spr2* mutants of *S. lycopersicum*^93^. Although primarily known for its role in biotic stress responses^94^, SA has been reported to improve manganese tolerance via nutritional and antioxidant regulation^95^. Taken together, our results show that GSNO-HAs-Fe-HAP NPs enhance Cd tolerance in maize seedlings through phytohormonal regulation.

Collectively, the findings of this study suggest that the GSNO-HAs-Fe-HAP NPs function through a multifaceted mechanism involving: prolonged NO release via GSNO decomposition; Improved nutrient delivery under acidic rhizosphere conditions; ROS detoxification via activation of antioxidant enzymes; osmotic balance through proline, carbohydrate, and sugar accumulation; Cd sequestration via enhanced *ZmPCs*, *ZmHMA9*, and *ZmPCR* expression; restoration of secondary metabolism and hormonal homeostasis to protect the plants under Cd stress and simultaneously improve the soil structure and plant growth (Supplementary Fig. 18).

From an agronomic perspective, GSNO-HAs-Fe-HAP NPs represent a sustainable and environmentally friendly alternative to conventional fertilizers, particularly for use in heavy metal-contaminated soils. Their ability to simultaneously alleviate abiotic stress, improve nutrient use efficiency, and stimulate key physiological and molecular pathways positions them as a valuable tool for climate-resilient and sustainable agriculture. Moreover, their slow-release properties reduce nutrient leaching and environmental runoff, aligning with global efforts to reduce fertilizer overuse and mitigate pollution. Future studies should focus on field-scale evaluations, long-term ecological impacts, and the optimization of formulation ratios for different crop species and soil types.

This study establishes GSNO–HAs–Fe–HAP NPs not merely as a nutrient source, but as a “precision microbiome engineer” that actively reshapes the rhizosphere to combat Cd stress, demonstrating a fundamental paradigm shift from passive nutrient supplementation to active biological modulation. This shift is evident in the critical limitation of the conventional NPK fertilizer^96^, which failed to recruit specific stress-tolerant microbes under Cd stress, maintaining a generic fungal community indistinguishable from the Control. In stark contrast, the GSNO–HAs–Fe–HAP NPs triggered a massive, targeted bloom of Tausonia, suggesting that the superior efficacy of our nanocomposite stems from its signaling capability rather than bulk nutrition. Hence, it is evident that the NPs treatment selectively amplified a phylogenetically distinct evolutionary lineage to cope with Cd stress, a finding corroborated by published literature^97–100^. We propose that the sustained release of Nitric Oxide (NO) and Humic Acids (HAs) may act as specific “chemotactic cues” or “metabolic triggers” functioning as molecular switches, specifically activating NO-responsive pathways in Tausonia to mobilize soil biological defenses.

Beyond redefining the signaling role of fertilizers, this capability offers a groundbreaking solution to the persistent challenge of poor survival rates in exogenous microbial inoculants^101^. By decoupling beta diversity metrics, we confirmed that Tausonia was not an introduced species but a latent member of the “rare biosphere” selectively amplified by the treatment. This “Indigenous Biostimulation” or “Sleep-to-Wake” strategy—awakening dormant indigenous extremophiles—negates the colonization barriers that often cause commercial bio-fertilizers to fail^102^, serving as a precision potentiator that enables the crop to recruit its own “immune system” directly from the soil reservoir. Crucially, this targeted specialization does not compromise long-term soil health, a common trade-off with high-input fertilization that often leads to bacterial dysbiosis^103,104^. Instead, GSNO–HAs–Fe–HAP NPs exhibit a “Dual-Track” mode of action: promoting fungal specialization for active defense while simultaneously preserving bacterial homeostasis for ecological stability. This balance confirms the nanocomposite as an ecologically biocompatible solution for recultivating metal-contaminated soils, ensuring that immediate stress tolerance is achieved without sacrificing future soil fertility. Ultimately, GSNO–HAs–Fe–HAP NPs represent a special class of “Smart Fertilizers” that integrate nutrient delivery with NO-mediated microbiome engineering, providing a blueprint for the next generation of precision agriculture where fertilizers are designed to strategically manipulate the rhizosphere network for enhanced resilience and sustainability.

The accumulation of heavy metals in edible tissues and the simultaneous decline in nutritional quality represent a dual threat to food safety and security. In this study, Cd stress led to a marked surge in grain Cd concentration accompanied by precipitous drops in protein and carbohydrate contents, reflecting the deleterious effects of Cd on phloem loading, nitrogen assimilation, and source-sink transport. It is important to note that during the reproductive stage, the experimental plants were subjected to concurrent biotic stress (i.e., aphid infestation), which physically compromised grain filling and sink development across all treatment groups, as evidenced by the incomplete cob formation. However, biochemical profiling revealed that the GSNO-HAs-Fe-HAP NPs maintained their physiological efficacy even under these severe multi-stress conditions. The nanocomposite effectively minimized Cd entry into the grains, outperforming conventional NPK fertilizers, which suggests a robust restriction of acropetal translocation likely mediated by rhizosphere immobilization and NO-regulated transporter suppression^105^. More crucially, despite the physical limitations in grain development caused by biotic factors, the nutritional density of the grains was significantly restored; the recovery of protein and carbohydrate concentrations to near-control levels indicates that the nanocomposite successfully protected primary metabolism (nitrogen assimilation and photosynthesis) and sustained the phloem transport of assimilates to the reproductive sinks. This implies that the sustained release of NO and nutrients provided a systemic metabolic buffer, allowing the plant to prioritize nutrient deposition into the seeds even when physical sink expansion was hindered. Consequently, this study demonstrates that GSNO-HAs-Fe-HAP NPs function as a resilient “smart fertilizer” that ensures food safety and nutritional integrity (bio-fortification) not only under heavy metal stress but also in the face of complex environmental challenges.

It is concluded that in this study, we developed a multifunctional smart fertilizer—GSNO-HAs-Fe-HAP—via a one-pot synthesis that immobilizes GSNO, HAs, and Fe ions onto an HAP matrix. This system enables sustained NO release for over 6 days while simultaneously delivering essential nutrients and stimulants in response to root exudates. The superior NO-release profile compared to GSNO solutions highlights the critical roles of HAs and Fe^3+^ ions in GSNO stabilization and reduction on the HAP surface. Furthermore, the accelerated release of crop nutrients (i.e., calcium, phosphate, and ferric ions) and stimulants (i.e., HAs) in the presence of citric acid—a representative component of root exudates—demonstrates that crop exudates can directly disrupt non-covalent interactions among GSNO, HAs, and metal ions, thereby enhancing plant nutrient use efficiency.

While the present study demonstrates the efficacy of GSNO-HAs-Fe-HAP NPs under a relatively high-concentration cadmium stress (400 μM for two weeks), other, low-dose treatments my be tested to represent the chronic, low-concentration exposure. Future studies should therefore evaluate the performance of these nanoparticles under variable Cd concentrations or Cd-contaminated soils.

Under Cd stress, GSNO-HAs-Fe-HAP NPs significantly reduced Cd uptake in maize plants, preserving plant growth and photosynthetic efficiency by modulating redox homeostasis and minimizing oxidative damage. The treatment enhanced osmolyte accumulation and secondary metabolite biosynthesis, thereby conferring greater Cd tolerance than conventional NPK fertilizers. Controlled NO release also elevated the levels of NO-mediated PCs, enhancing metal chelation and further alleviating Cd-induced damage in maize seedlings (Supplementary Fig. 18). Concurrently, GSNO-HAs-Fe-HAP NPs upregulated endogenous hormone levels and metal-transporter gene expression, strengthening the plant’s defense responses. Beyond plant-level benefits, the treatment also improved soil properties by increasing nutrient availability, OM content, and nitrogen cycling—fostering a more sustainable soil ecosystem under Cd stress (Supplementary Fig. 18). These findings demonstrate that GSNO-HAs-Fe-HAP NPs serve as a next-generation smart fertilizer, offering superior Cd tolerance and soil health benefits compared to conventional NPK fertilizers. Yet, to evaluate the broader potential application of GSNO-HAs-Fe-HAP NPs, future studies should investigate their field-scale feasibility and long-term environmental impacts.

## Methods

### One-pot synthesis of GSNO-HAs-Fe-HAP nanoparticle composite

GSH (614 mg) and NaNO_2_ powder (138 mg) were dissolved in 1 M HCl (2 mL) and deionized water (2 mL), respectively, to prepare a 0.5 M GSNO solution. The solution was vortexed vigorously until fully dissolved, then diluted 5-fold with distilled water. Pure HAP powder (1000 mg) was then dispersed into the diluted GSNO solution.

Parallel to this, HAs (1 g) were dispersed in deionized water (30 mL), thoroughly vortexed, and centrifuged at 15,900 g for 10 min to obtain the water-soluble fraction. Subsequently, the supernatant (8 mL) and FeCl_3_ (400 mg) were added to the GSNO-HAP mixture, followed by the dropwise addition of 1 N NaOH until the pH reached 10. The resulting GSNO-HAs-Fe-HAP particles were collected by centrifugation (2500 g, 5 min) and washed three times with distilled water. The final pellet was stored at −70 °C for 2 days, followed by freeze-drying for 3 days to obtain lyophilized GSNO-HAs-Fe-HAP NPs.The obtained particles were finely ground using a mortar and pestle and then used for subsequent experiments. The other types of particles were prepared using the same procedure described above, except that different coating materials were used. Glutathione reduced (GSH) and HAP (Ca_10_(PO_4_)_6_(OH)_2_, 95%) were purchased from FUJIFILM Wako Pure Chemical Corporation (Japan) and OCI Company Ltd (Korea), respectively. Sodium nitrite (NaNO_2_) and citric acid were obtained from Sigma-Aldrich, while iron (III) chloride (FeCl_3_), nitric acid (70%), hydrochloric acid (HCl, 1 N), sodium hydroxide (NaOH, 1 N Sodium dihydrogen phosphate (NaH_2_PO_4_, 99%), disodium hydrogen phosphate (Na_2_HPO_4_, 99%), and sodium azide (NaN_3_, 99%) were purchased from Daejung Chem (Korea). Concentrated HCl solution (36%) and natural HAs ( > 70 wt% humic matter) were obtained from Samchun (Korea) and Mycsa AG (Texas), respectively. Poly(styrene sulfonate) standard was obtained from PSS-Polymer (Maryland).

### Characterization of GSNO-HAs-Fe-HAP NPs

To evaluate the impact of each component on GSNO immobilization, four different nanoparticle composites—GSNO-HAP, GSNO-HAs-HAP, GSNO-Fe-HAP, and GSNO-HAs-Fe-HAP—were synthesized. The resulting particles were freeze-dried for 3 days, and their dry weights were measured. Thermal stability and molecular interactions were analyzed using Thermogravimetric analysis (TGA) and Fourier-transformed infrared (FTIR) spectroscopy, respectively. Elemental compositions of nanoparticle composite were also determined (for detailed methodology, see Supplementary Text 1). The total organic and inorganic fractions, as well as the amounts of GSNO, Fe, and HAs, were estimated based on TGA, ICP-OES, and dry-weight measurements of the nanoparticle composites (for detailed methodology, see Supplementary Text 1). Furthermore, zeta potential, hydrodynamic size, and Brunauer–Emmett–Teller (BET) surface area analyses were also conducted for HAP and GSNO-HAs-Fe-HAP (for detailed methodology, see Supplementary Text 2). Inductively coupled plasma–optical emission spectrometry (ICP-OES) was undertaken to quantify Ca, P, and Fe released from GSNO-HAs-Fe-HAP and Fe and S contents in the coating layers of nanoparticle composites (for detailed methodology, see Supplementary Text 3). Size Exclusion Chromatography (SEC) was performed to assess HA desorption from the GSNO-HAs-Fe-HAP complex using a Waters 2695 system with a Waters 996 photodiode array detector and a PolySep GFC-P3000 (300 × 7.80 mm) column coupled with a PolySep GFC-P (35 × 7.80 mm) safety guard column (Phenomenex) (for detailed methodology, see Supplementary Text 4).

High-performance liquid chromatography–electrospray ionization quadrupole time-of-flight mass spectrometry (HPLC-ESI-Q-TOF MS) was employed to investigate the molecular transformations of GSNO within the GSNO-HAs-Fe-HAP complex (for detailed methodology, see Supplementary Text 5). Structural characterization was performed via transmission and scanning electron microscopy (TEM – Hitachi, HT7700 and SEM – Hitachi, SU8230). Elemental composition was determined using Energy Dispersive X-Ray (EDX) spectroscopy (OXFORD, Ultim Max100) (for detailed methodology, see Supplementary Text 6).

Finally, a highly sensitive, ozone-chemiluminescence-based, comparative analysis of NO release kinetics from the canonical NO donor GSNO and GSNO-HAs-Fe-HAP NPs was conducted using a Sievers NOA-280i Nitric Oxide Analyzer at 25 °C continuously for 7 days^87^. To compare the NO release kinetics of free GSNO and the GSNO–HAs–Fe–HAP nanocomposite, the amount of pure GSNO used as the control was determined quantitatively based on Supplementary Table S3. Because 1 g of the GSNO–HAs–Fe–HAP nanocomposite contained 59.1 mg of GSNO, 59.1 mg of pure GSNO powder was used as the equivalent control. Accordingly, 1 g of the nanocomposite or 59.1 mg of pure GSNO powder was added to 1 mL of distilled water. A 1 M aqueous citric acid solution was then added dropwise to adjust the pH of both the GSNO solution and the nanocomposite suspension. The bottles were immediately sealed tightly, and the NO gas released from free GSNO and the nanocomposite was measured under dark conditions. Data acquisition was initiated immediately after sample preparation. The detailed methodology for the measurement of S-nitrosothiols is provided in Supplementary Text S1^1^.

### Plant material, growth conditions, experimental design

Maize seeds [variety Suwonchal 97 (a single-cross F1 hybrid developed by the Rural Development Administration - RDA, South Korea, currently awaiting official cultivar registration)] were surface-sterilized with 70% ethanol, washed three times with autoclaved distilled water, and germinated in Petri dishes at 25 °C in a germination chamber (LG Taeyoung, Model L061G1). After 4 days, germinated seedlings were transplanted into pots (12 cm in diameter and height) containing 1.2 kg of sandy soil. A 3-cm-deep hole was made in the center of each pot (except for controls) for applying NPK or GSNO-HAs-Fe-HAP NPs, after which the seedlings were placed in the same holes to continue growing. Each pot received a commercial NPK fertilizer at a rate of 198.6 mg N, 92 mg P, and 184 mg K, in line with standard agricultural practices. GSNO-HAs-Fe-HAP NP treatments included 0.68 g of NPs per pot, based on the P content in HAP. Compositional analysis revealed that the NPs contained 72.8% HAP, of which 18.5% was P, corresponding to approximately 0.135 g of P per gram of NPs. Equal amounts of N and K were supplemented to match the NPK treatment.

The plants were grown under controlled conditions (16-h light/8-h dark cycle, 28 °C ± 2 °C) in a greenhouse at Kyungpook National University, South Korea, and irrigated every 3 days with 200 mL of ½-strength Hoagland solution. When the seedlings reached the V1 stage, they were divided into six treatment groups: i) Control, ii) Cd Control, iii) NPK, iv) Cd + NPK, v) GSNO-HAs-Fe-HAP NPs, and vi) Cd + GSNO-HAs-Fe-HAP NPs. Thereafter, Cd-treated groups were irrigated daily with 200 mL of 400 µM CdCl_2_ per pot for 2 weeks to evaluate the protective capacity of GSNO-HAs-Fe-HAP NPs under high Cd stress. Twenty-one days after transplanting, the seedlings were harvested, photographed, and separated into leaves, stem, and roots, then stored at -80 °C for further analyses.

Experiments were also set up using the NO scavenger, 2(4 Carboxyphenyl) 4,4,5,5 Tetramethylimidazoline 1 Oxyl 3 Oxide (cPTIO). For this purpose, the maize seedlings were grown under Cd stress (400 µM CdCl_2_) for two weeks with the following treatments: Cd alone, Cd + cPTIO, HAs–Fe–HAP (without GSNO), Cd + HAs–Fe–HAP (without GSNO), Cd + GSNO-HAs–Fe–HAP NPs, and Cd + GSNO-HAs–Fe–HAP NPs + cPTIO (100 µM). After 2 weeks of the treatment, maize seedlings were harvested for further analyses. cPTIO was applied every three days during the treatment period to efficiently scavenge nitric oxide. Leaves, stems, and roots were collected separately to assess oxidative stress and antioxidant responses. Samples were immediately frozen in liquid nitrogen and stored at −80 °C until use. All experiments were performed in triplicate using independent biological replicates.

Experiments were also conducted to collect data on the long-term impact of the NPs. For this purpose, maize seedlings were transferred to soil inside a greenhouse. The treatments were applied twice during the entire growth period: an initial application at the time of transplantation as described above and a second at the tasseling stage to ensure sustained treatment supply and stress mitigation during the reproductive phase. Data on shoot height, stem diameter, leaf number, and biomass were collected until the plants reached the harvest stage. Finally, the rhizospheric soil was collected for chemical analysis and microbiome analysis via sequencing. Bulk soil was subjected to ICP-OES to determine the nutrient profile. After the harvest, grain Cd content was determined, and data on various yield and grain-related parameters were collected.

### Fluorescein-labeled GSNO-HAs-Fe-HAP NPs

To visualize GSNO-HAs-Fe-HAP NP uptake in maize tissues, the NPs were labeled with fluorescein isothiocyanate (FITC) following the method of Hussain et al.^106^. Briefly, GSNO-HAs-Fe-HAP NPs (20 mg) were dissolved in dimethylformamide and mixed with 3-aminopropyltriethoxysilane (APTES), then incubated at room temperature for 24 h. The mixture was then washed three times with ethanol to remove unbound APTES. The resulting particles were suspended in 10 mL of ethanol containing 1 g of FITC and stirred continuously at 52 g for 24 h to allow the reaction between FITC and APTES to occur. FITC-labeled NPs were then centrifuged and washed three times with ethanol and deionized water to remove unbound FITC. GSNO-HAs-Fe-HAP NPs accumulation in plant tissues was examined using confocal laser scanning microscopy (Zeiss LSM 800, Carl Zeiss, Germany) at 495/519 (excitation/emission). Cross-sections of leaves, stem, and roots ( ≤ 5 cm from root junction) were manually prepared using a sharp blade, with potato slices serving as a support medium.

### Quantification of photosynthetic pigments

Chlorophyll and carotenoid contents were determined as described by Methela, et al.^10^. Briefly, fresh leaf samples (0.1 g) were homogenized in 20 mL of DMSO at 65 °C for 4 h and then allowed to cool before centrifugation at 18,500 g for 15 min. Absorbance of the supernatant was measured at 663, 645, and 470 nm using a Multiskan GO Microplate Spectrophotometer (Thermo Scientific). Chlorophyll a, b, and carotene contents were calculated based on Arnon’s equations.

Gas exchange parameters, including net photosynthetic rate (A), transpiration rate (E), intercellular CO_2_ concentration (Ci), and stomatal conductance (gs), were measured using a portable photosynthesis system (LCpro T, ADC BioScientific Ltd., UK). Measurements were performed on the youngest fully expanded leaves under the respective treatment conditions. Measurements were taken every two weeks during the approximately 60-day growth period. The measurements were conducted between 09:00 and 11:00 a.m. under ambient CO_2_ concentration, leaf temperature maintained at 25 °C ± 2 °C, relative humidity of 50–60%, and photosynthetic photon flux density (PPFD) of 2000 µmol m^−2^ s^−1^. Data for A, E, Ci, and gs were automatically computed by the instrument based on the differences in CO_2_ and H_2_O concentrations between reference and sample air streams, normalized to the enclosed leaf area. Fv/Fm was measured using a portable chlorophyll fluorescence system (OS5p + ; Opti-Science, Hudson, NH, USA). Then, the maximum fluorescence (Fm) was determined using a saturating pulse (2000 μmol m^–2^ s^–1^) of .8 -s duration. The activity of photosystem II (PSII) was determined by FV/FM, when the leaves had been dark-adapted for 30 min, using the dark leaf clip^107^.

### Quantifying Cd in soil, plant tissues, and grains

Rhizospheric soil samples, plant tissues, and grains were collected from plants grown under the respective treatments. ICP analysis was performed to quantify the accumulated Cd using an inductively coupled plasma optical emission spectrometer (ICP-OES; Avio 500/550, PerkinElmer, USA)^108^. Briefly, freeze-dried samples were digested in 3 mL of HNO_3_ at 95 °C for four hours using a heat block. The digested solution was filtered through a 0.45 µM syringe filter (Sterlitech, Auburn, WA, USA) and analyzed.

To quantitatively evaluate the physiological responses of maize seedlings to cadmium (Cd) stress and the mitigating effects of the treatments, the Cd tolerance index (TI) was calculated at the V5 stage (five fully developed leaves) based on fresh biomass^109,110^. Following the designated treatment periods, the plants were carefully harvested and washed thoroughly with distilled water to remove any adhering soil or growth medium. Surface moisture was gently blotted dry using absorbent paper towels, and the fresh weight (FW) of the roots and shoots was measured immediately using an analytical balance. The Tolerance Index (TI) was determined to assess the relative growth performance under Cd exposure compared to the unstressed conditions. The TI for fresh weight was calculated using the following equation.

1 $${{\rm{TI}}}(\%)=\frac{{FW}_{treatment}}{{FW}_{control}}\times 100$$

### ROS detection via staining

H_2_O_2_ accumulation was visualized by staining leaves with 10 mM DAB for 12 h on a shaker, followed by pigment removal in 96% ethanol^111^. Superoxide anion (O_2_•^−^) was detected using NBT staining in a solution of 50 mM phosphate buffer (pH 7.0), 0.1% NBT, and 10 mM sodium azide under dark conditions for seven hours^112^.

### Quantification of H_2_O_2_ content and lipid peroxidation

Quantitative estimation of H_2_O_2_ was performed using a potassium iodide-based assay, with absorbance (A) measured at 390 nm^113^. Lipid peroxidation was assessed by measuring MDA levels using a trichloroacetic acid-based method^114^. Both assays were performed using leaf, stem, and root samples (for detailed methodology, see Supplementary Text 7).

### Determination of antioxidant enzyme activities

The activities of superoxide dismutase (SOD), peroxidase (POD), ascorbate peroxidase (APX) enzymes, and GSH/GSSG contents were determined spectrophotometrically. Tissue samples (0.1 g) from leaves, stem, and roots were individually homogenized in phosphate buffer and centrifuged at 18,500 g for 15 min. Absorbance changes at specific wavelengths were used to quantify enzyme activity^115–117^. Catalase (CAT) activity was measured using an OxiTec Catalase assay kit (BO-CAT-400, BIOMAX). The GSH/GSSG contents were measured using the OxiTec Glutathione assay kit (BO-CAT-400-BO-GLU-200, BIOMAX)

### Quantification of osmolytes

Proline content was quantified using a ninhydrin-based colorimetric assay, with absorbance measured at 520 nm^118^. Total protein was extracted in phosphate-buffered saline (PBS) and quantified using the Bradford protein assay at 595 nm^119^. Total carbohydrates were determined using the phenol–sulfuric acid method^120^, with absorbance measured at 490 nm. Total soluble sugars were measured using anthrone reagent assay following incubation of samples at 90 °C, with absorbance read at 620 nm^121^. Results were expressed as µmol g^-1^ FW for proline and mg glucose g^-1^ FW for carbohydrates and sugars (for detailed methodology, see Supplementary Text 8).

### Metabolite profiling and PAL activity

Phenylalanine ammonia-lyase (PAL) activity was assessed by incubating enzyme extracts with L-phenylalanine at 37 °C and measuring absorbance at 290 nm. PAL activity was expressed in units per gram of protein using a cinnamic acid standard curve^122^. Total flavonoid content was determined using aluminum chloride colorimetry, with absorbance measured at 510 nm^123^, reported as milligrams of quercetin equivalents per gram of sample. Total phenolic content was measured using the Folin–Ciocalteu method^124^, with absorbance measured at 760 nm and reported as milligrams of gallic acid equivalents per gram of sample (for detailed methodology, see Supplementary Text 9). For a comprehensive secondary metabolite profiling, MS/MS-LC-ESI-Q-TOF mass spectrometry was conducted following the method of Guijarro-Díez, et al.^125^ (for detailed methodology, see Supplementary Text 10).

### Quantification of SNO content

S-nitrosothiol (SNO) levels in the plant samples were quantified using a Sievers NOA-280i Nitric Oxide Analyzer, following the method of Hussain, et al.^87^. Results were expressed as nM μg^-1^ protein, calibrated against a CysNO standard curve (for detailed methodology, see Supplementary Text 11).

### Quantitative real-time PCR

Quantitative real-time PCR (qRT-PCR) was performed to assess the expression of genes encoding antioxidant enzymes (*SOD*, *POD*, *CAT*, and *APX*), S-nitrosoglutathione reductase (*GSNOR*), heavy metal transporters (*PCR*, *HMA4*, *HMA9*, and *NRAMP5*), PC-metal complexes transporter *ABCC1*, ATP-binding cassette transporters (*ABCG40*), Cd resistance genes (*PCR* and *PCS*), and those involved in secondary metabolism (*P5CR1* and *PAL*). Total RNA was extracted from tissues ground in liquid nitrogen using the Biofact Total RNA Kit, and cDNA synthesis was conducted with the Solgt RT-kit. qRT-PCR analysis was performed on a Bio-Rad CFX Duet system using SYBR Green with initial denaturation at 95 °C for 1 min, followed by 40 cycles at 95 °C for 15 s, 60 °C for 15 s, and 72 °C for 20 s. Relative gene expression levels were calculated using the 2^−ΔΔCt^ method, with gene-specific primers (Supplementary Table 1).

### Phytohormone quantification (ABA, JA, and SA)

To assess the effects of different treatments under Cd stress, endogenous levels of ABA, JA, and SA were measured in maize seedlings. The levels of ABA, JA, and SA were determined following the methods of Khan, et al.^126^, McCloud and Baldwin^127^, and Seskar, et al.^128^, respectively, with minor modifications (for detailed methodology, see Supplementary Text 12).

### Analysis of soil physicochemical properties

To determine the impact of nanoparticle composites on the soil profile, soil samples were collected, air-dried, ground, and sieved for further analyses. Soil pH and electrical conductivity (EC) were measured in a 1:10 (w/v) soil-to-water suspension using a dual pH/EC meter (HMM-100Pro, Hanyoung system, Seoul, Korea). Organic matter content was assessed using the Walkley–Black method^129,130^. Available phosphorus was extracted using the Bray and Kurtz method^131^, and analyzed using the molybdenum blue method. Total nitrogen and ammonium/nitrate nitrogen were measured using the Kjeldahl method^132^. Cation exchange capacity (CEC) and exchangeable cations (K, Ca, Mg, and Na) were determined using ammonium acetate extraction followed by titration, as described by Ross and Ketterings^133^.

### Yield estimation and grain quality

To assess the long-term impact of GSNO-HAs-Fe-HAP NPs on reproductive growth and grain quality, a subset of maize plants was grown until physiological maturity, with harvesting conducted at ~120 days after transplanting. To determine the concentration of Cd and essential minerals (P and K) in the grains, 0.5 g of the grain flour was digested in HNO_3_ and analyzed using Inductively Coupled Plasma Optical Emission Spectroscopy (ICP-OES, Avio500, PerkinElmer, USA). Grain nutritional quality was evaluated by quantifying total protein and carbohydrate contents.

### Rhizosphere soil sampling and metagenomic sequencing

To investigate the impact of GSNO-HAs-Fe-HAP NPs on the rhizospheric microbial community, soil samples were collected at the harvesting stage from the six treatment groups (Control, Cd+Control, NPK, Cd+NPK, GSNO-HAs-Fe-HAP NPs), and Cd+GSNO-HAs-Fe-HAP NPs. For each treatment, rhizosphere soil was collected from five biological replicate maize seedlings and thoroughly mixed to form a single composite sample. This pooling strategy was employed to minimize individual variation and obtain a representative microbiome profile for each treatment group, resulting in a total of six composite samples for metagenomic analysis.

Briefly, the maize plants were carefully uprooted, and the loosely adhering bulk soil was removed by gentle shaking. The rhizosphere soil, defined as the soil tightly adhering to the root surface (approximately 0–4 mm from the root), was then collected using a sterile brush. All collected samples were immediately flash-frozen in liquid nitrogen and stored at –80 °C until genomic DNA extraction. DNA was extracted from soil samples using the Dneasy PowerSoil Pro kit (QIAGEN, Germany) according to the manufacturer’s instructions. The hypervariable V3–V4 region of the bacterial 16S rRNA gene and the fungal ITS2 region were amplified from the purified DNA using KAPA HiFi HotStart ReadyMix (Roche, Switzerland) for 25 PCR cycles. Amplification was performed with barcoded fusion primers containing Nextera adapters: 341 F (5′-CCTACGGGNGGCWGCAG-3′) and 805 R (5′-GACTACHVGGGTATCTAATCC-3′) for the 16S rRNA V3–V4 region, and fITS9F (5′-AACGCAGCAAAATGCGA-3′) and ITS4R (5′-TCCTCCGCTTATTGATATGC-3′) for the ITS2 region. The resulting PCR products ( ~ 600 bp for 16S V3–V4 and ~550 bp for ITS2) were purified using HiAccuBeads (AccuGene, South Korea). Purified amplicons were quantified, pooled at equimolar concentrations, and sequenced on an Illumina MiSeq i100 Plus system using the MiSeq i100 Series Reagent Kit with 600 cycles (Illumina, USA).

### Metagenomic sequence analysis

Raw sequencing reads targeting the bacterial 16S rRNA gene V3–V4 region and the fungal ITS2 region were denoised by correcting amplicon errors and used to infer exact amplicon sequence variants (ASVs) using DADA2 v1.16^134^. Taxonomic classification was performed using Naïve Bayes classifiers trained on the SILVA release 138 rRNA reference database (https://www.arb-silva.de/documentation/release-138/) for V3–V4 sequences and the UNITE release 8.0 fungal ITS reference database for ITS2 sequences^135^. All downstream analyses of quality-filtered and chimera-filtered reads were conducted using the QIIME2-2022.2 software package^136^. The resulting taxonomic assignments were used for downstream analyses. To assess microbial richness and evenness, multiple alpha diversity indices were calculated, including Observed Features, Shannon, Simpson, Faith’s Phylogenetic Diversity (Faith PD), and Chao1, using the QIIME2 platform. Beta diversity, reflecting differences in microbial community composition among samples, was assessed using Jaccard dissimilarity, Bray–Curtis distance, unweighted UniFrac, and weighted UniFrac metrics. Principal Coordinate Analysis (PCoA) was performed based on each distance metric to visualize patterns of microbial community dissimilarity.

### Prediction of S-nitrosylation sites in ZmPCS

Putative S-nitrosylation sites in *Zea mays* phytochelatin synthase (ZmPCS; Zm00001eb338260) were predicted using the GPS-SNO 1.0^137^ with a high threshold. Cysteine residues exceeding a cut-off value of 2.443 were selected for further analysis. The three-dimensional structure of ZmPCS was obtained from the AlphaFold Protein Structure Database and analyzed using PyMOL v2.5^138^. Solvent-accessible surface area (SASA) values for all cysteine residues were calculated with FreeSASA^139^ (v2.0). Total, side-chain, and main-chain SASA values were extracted, and the feasibility of S-nitrosylated Cys residues was assessed based on side-chain SASA, which reflects thiol exposure to solvent and reactive nitrogen species^139^. Cysteine residues were visualized on the ZmPCS structural model in caroon/ribbon mode, and residue numbering was cross-validated by aligning the AlphaFold model with the ZmPCS FASTA sequence. The potentially S-nitrosylated Cys sites were shown in different colors.

### Statistical analysis

Analysis of variance (ANOVA) was conducted using Statistix 10 to evaluate the interactive effects of Cd toxicity and nutrient supplementation (NPK or GSNO-HAs-Fe-HAP NPs). Post hoc comparisons were performed using Tukey’s tests, with statistical significance determined at *p* < 0.05. Significant differences among treatments were indicated by different letters (e.g., a, b, c) above the bars in the figures. Bar graphs were generated using GraphPad Prism 9 (GraphPad Software, Inc., La Jolla, CA, USA). A circular heatmap was generated using SRplot (http://www.bioinformatics.com.cn/srplot), while Pearson’s correlation matrix was visualized using the metal package in RStudio (R 4.4.3).

### Reporting summary

Further information on research design is available in the Nature Portfolio Reporting Summary linked to this article.

## Supplementary information

Supplementary Information

Reporting Summary

Transparent Peer Review file

## Source data

Source Data

## Data Availability

Source data is available for Figs. 1, 2, 4–10, and Supplementary Figs. 3-17 in the associated source data file. The raw bacterial 16S rRNA V3-V4 and fungal ITS2 sequencing data generated in this study have been deposited in the NCBI Sequence Read Archive (SRA) under BioProject accession PRJNA1517259 and are publicly available at the NCBI BioProject database. Source data are provided with this paper.
