# Supplementary material for: GSNO-functionalized hydroxyapatite nanofertilizer for prolonged delivery, detoxification, and yield improvement in maize
Source: Nat Commun. 2026 Sep 16;17:10896. doi: 10.1038/s41467-026-77610-0 (PMC13677726; doi:10.1038/s41467-026-77610-0)
Supplement: Supplementary file 1 [file 41467_2026_77610_MOESM1_ESM.pdf]

SUPPLEMENTARY INFORMATION

GSNO-functionalized hydroxyapatite nanofertilizer for prolonged delivery, detoxification, and yield improvement in maize

Da-Sol Lee[1,#], Ho Young Yoon[2,3#], Ashim Kumar Das[1], Jun-Seo Kang[3,4], Bong-Gyu Mun[5], Adil Hussain[1,6], In-Jung Lee[1], Jong-Rok Jeon[3,4*], Byung-Wook Yun[1*]

[1]Department of Applied Biosciences, Kyungpook National University, Daegu 41566, Republic of Korea
[2]Laboratory of Natural Materials Technology, Åbo Akademi University, Henrikinkatu 2, Turku FI-20500, Finland
[3]Department of Applied Life Chemistry, Gyeongsang National University, Jinju 52828, Republic of Korea
[4]Institute of Agriculture & Life Science (IALS), Gyeongsang National University, Jinju 52828, Republic of Korea
[5]Department of Environmental and Biological Chemistry, Chungbuk National University, Cheongju, Republic of Korea
[6]Department of Agriculture, Abdul Wali Khan University Mardan, Mardan 23200, Khyber Pakhtunkhwa

[#]These two authors contributed equally.

giftanna@naver.com (D.-S. Lee)
hoyoung.yoon@abo.fi (H.-Y. Yoon)
ashim@knu.ac.kr (A.K. Das)
kjspopos@gnu.ac.kr (J.-S. Kang)
munbg@chungbuk.ac.kr (B.-G. Mun)
adilhussain@awkum.edu.pk (A. Hussain)
ijlee@knu.ac.kr (I.-J. Lee)
jrjeon@gnu.ac.kr (J-R Jeon)
bwyun@kn.ac.kr (B-W Yun)

ORCIDs:
https://orcid.org/0009-0005-7045-4619 (D.-S. Lee)
https://orcid.org/0000-0001-6012-4812 (A.K. Das)
https://orcid.org/0000-0002-6670-1315 (B.-G. Mun)
https://orcid.org/0000-0002-7803-429X (A. Hussain)
https://orcid.org/0000-0001-7154-4820 (I.-J. Lee)
https://orcid.org/0000-0002-5451-2365 (B.-W. Yun)
https://orcid.org/0000-0002-5815-7815 (J.-R. Jeon)

*Corresponding Author:
bwyun@knu.ac.kr (B.-W. Yun); jrjeon@gnu.ac.kr (J.-R. Jeon)

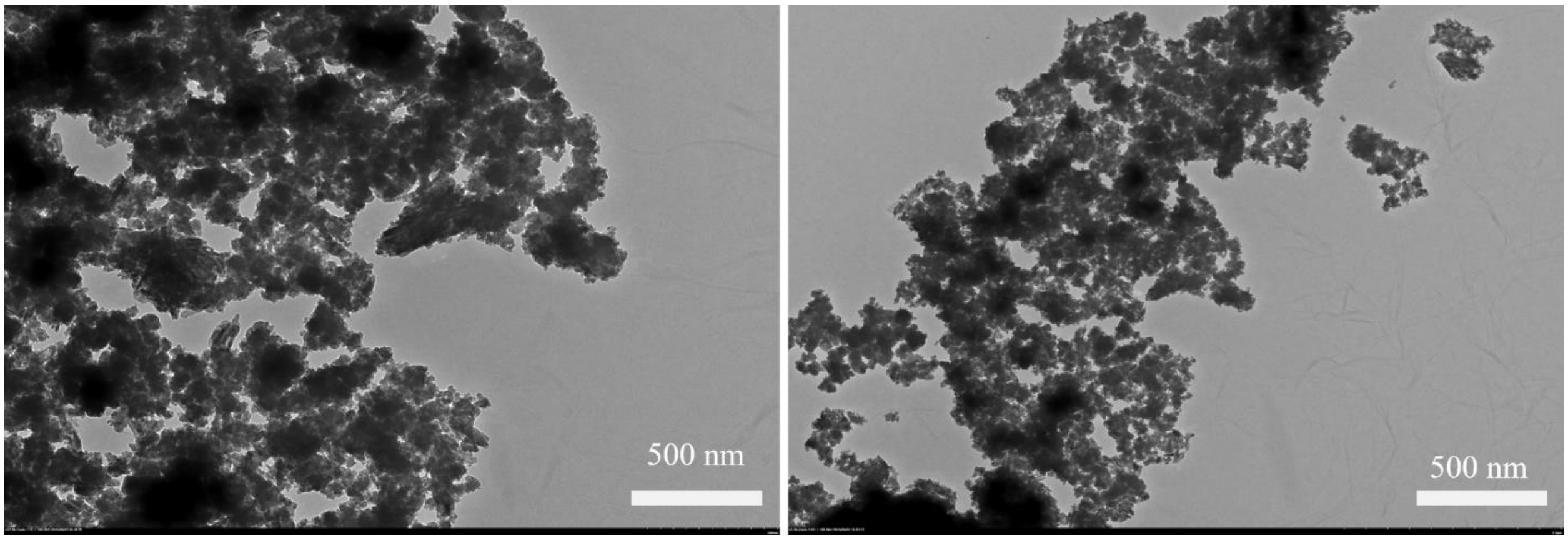

Supplementary Figure 1. Transmission Electron Microscopy images of GSNO-HAs-Fe-HAP NPs

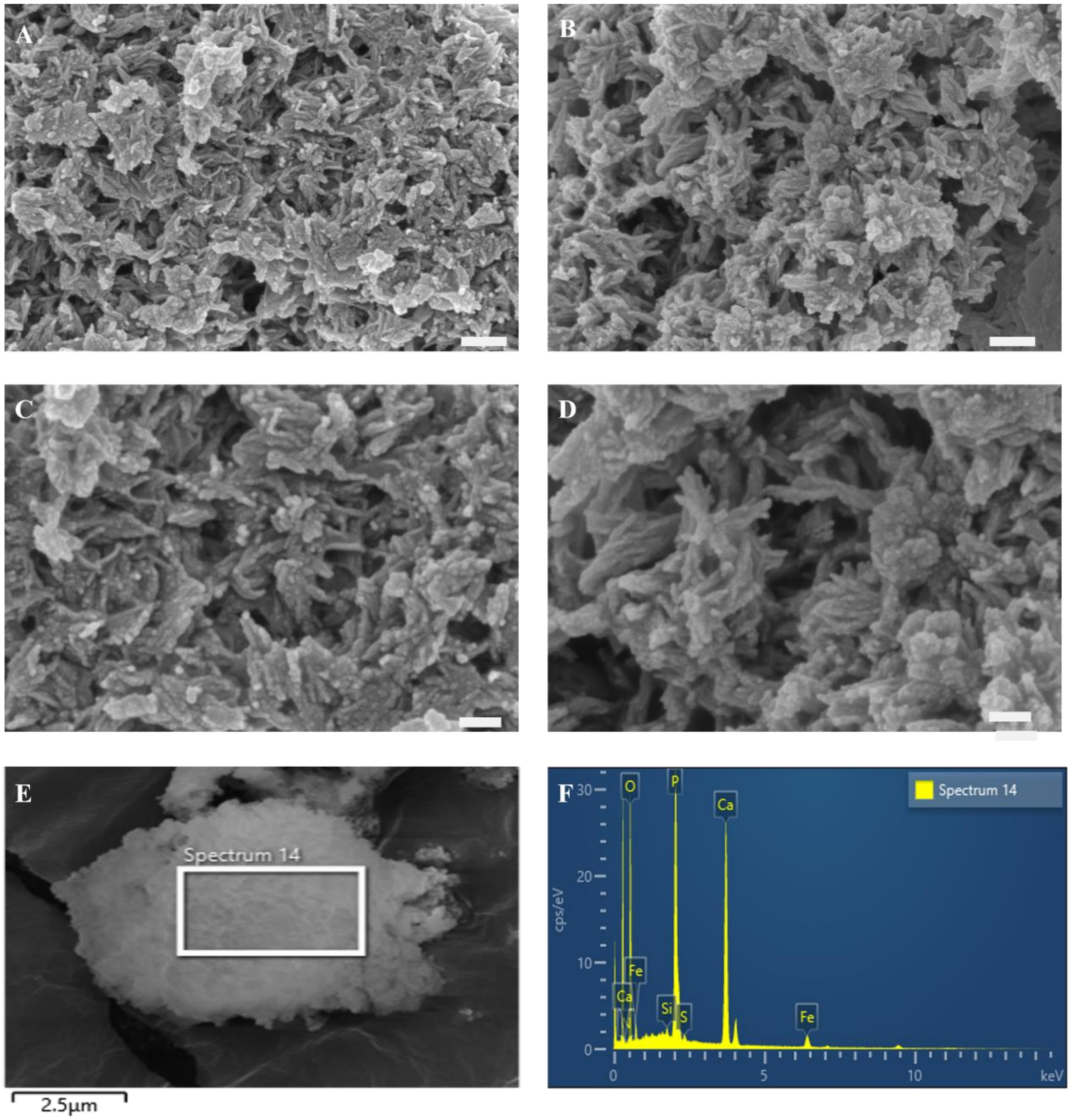

G

Spectrum 14				
**Element**	Signal Type	Wt%	Wt% Sigma	Atomic %
**N**	EDS	0.00	0.88	0.00
**O**	EDS	50.74	0.29	71.03
**Si**	EDS	0.44	0.05	0.35
**P**	EDS	13.58	0.14	9.82
**S**	EDS	0.30	0.05	0.21
**Ca**	EDS	29.01	0.21	16.21
**Fe**	EDS	5.92	0.19	2.38
**Total**		100.00		100.00

Supplementary Figure 2. Characterization of GSNO-HAs-Fe-HAP nanoparticle composite.

The GSNO-HAs-Fe-HAP nanoparticle composites was characterized via scanning electron microscopy (A–D), and energy dispersive X-ray spectroscopy analysis (E-G). Scale bars: A and B, 200 nm; C and D, 100 nm.

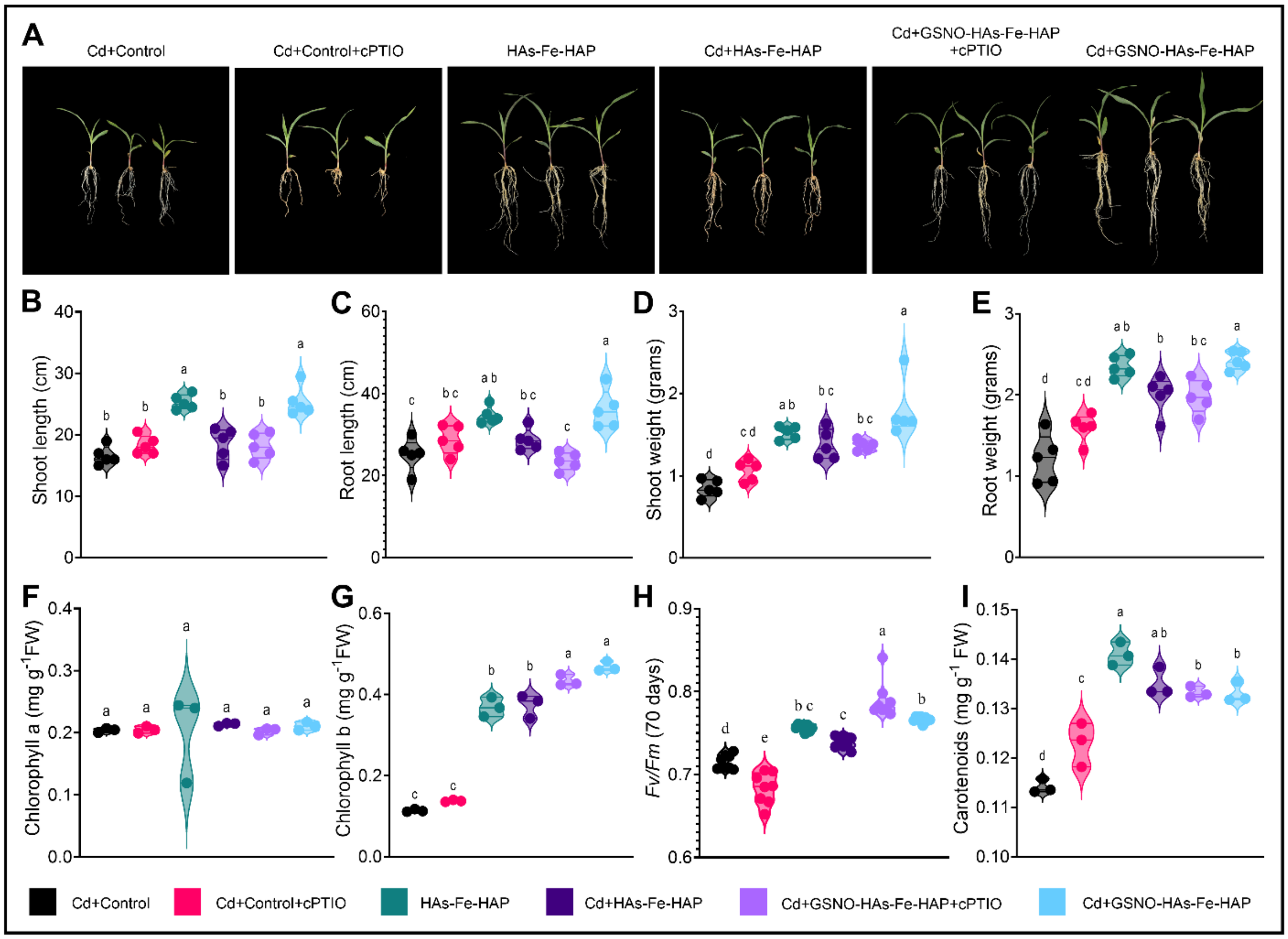

Supplementary Figure 3. Impact of GSNO-HAs-Fe-HAP NPs on maize growth and photosynthetic efficiency under Cd stress.

Effects of GSNO-HAs-Fe-HAP NPs on maize growth under Cd stress with and without the NO scavenger cPTIO were determined (A) by measuring shoot and root length (B,C), shoot and root weight (D,E), chlorophyll *a* (F), chlorophyll *b* (G), *Fv/Fm* (H), and total carotenoid content (I). Bars represent mean ± standard deviation. Different lowercase letters above the bars indicate statistically significant differences between treatments, determined using one-way ANOVA followed by Tukey's post hoc test at $p < 0.05$.

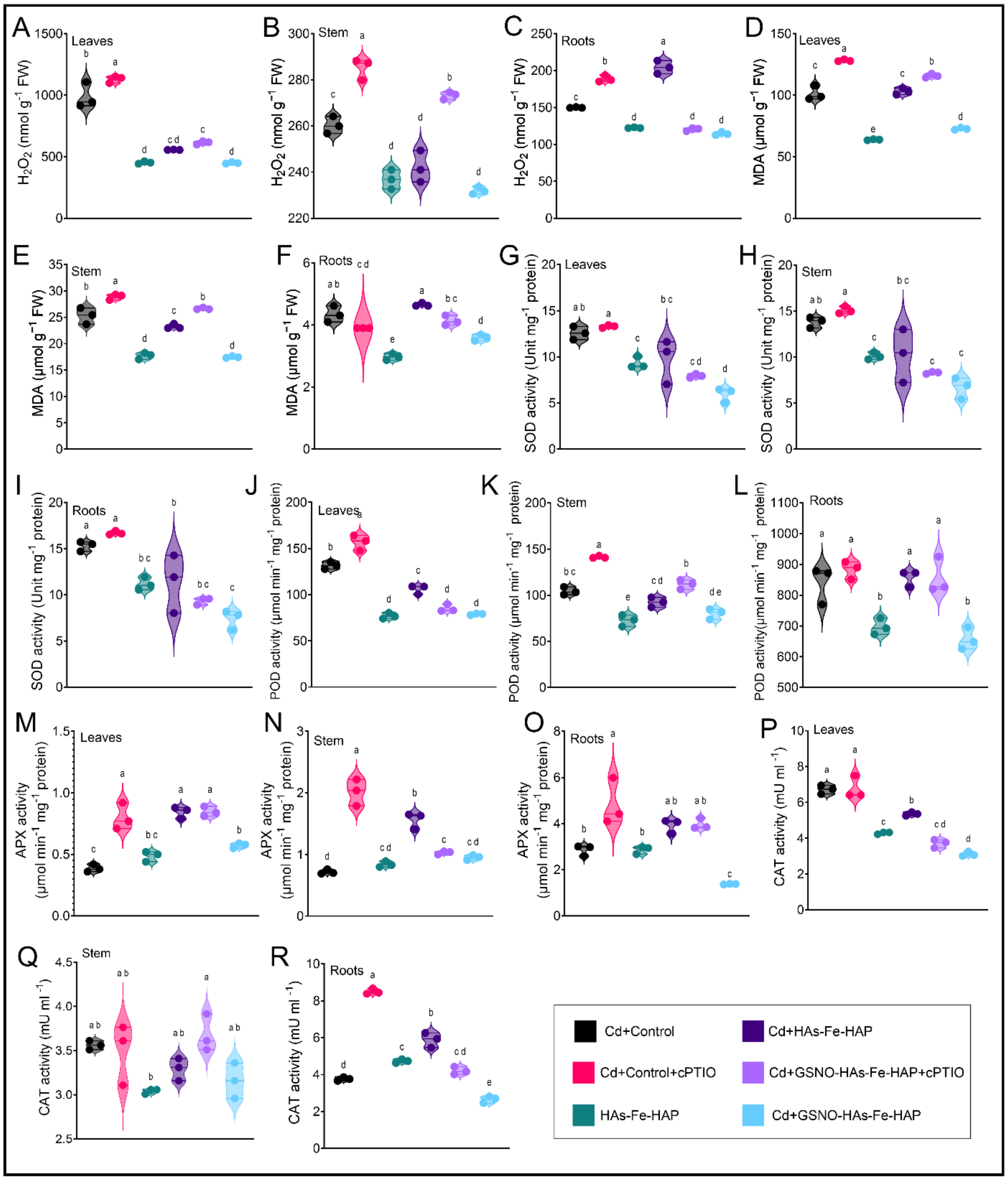

Supplementary Figure 4. Impact of GSNO-HAs-Fe-HAP NPs on redox homeostasis under Cd stress.

Effects of GSNO-HAs-Fe-HAP NPs on the redox homeostasis of maize seedlings under Cd stress were determined by measuring H_2O_2 (A-C), and MDA contents (D-F) in the leaves, stem, and roots of maize plants. Furthermore, the effects of the nanoparticle composite on antioxidant enzyme activities in maize seedlings exposed to Cd toxicity were determined by measuring the activity of SOD, (G–I), POD, (J–L), APX (M–O), and CAT (P-R) in the leaves, stem, and roots of maize seedlings. Data are presented as mean ± standard deviation. Different lowercase letters above the bars indicate statistically significant differences between treatments, determined using one-way ANOVA followed by Tukey's post hoc test at $p < 0.05$.

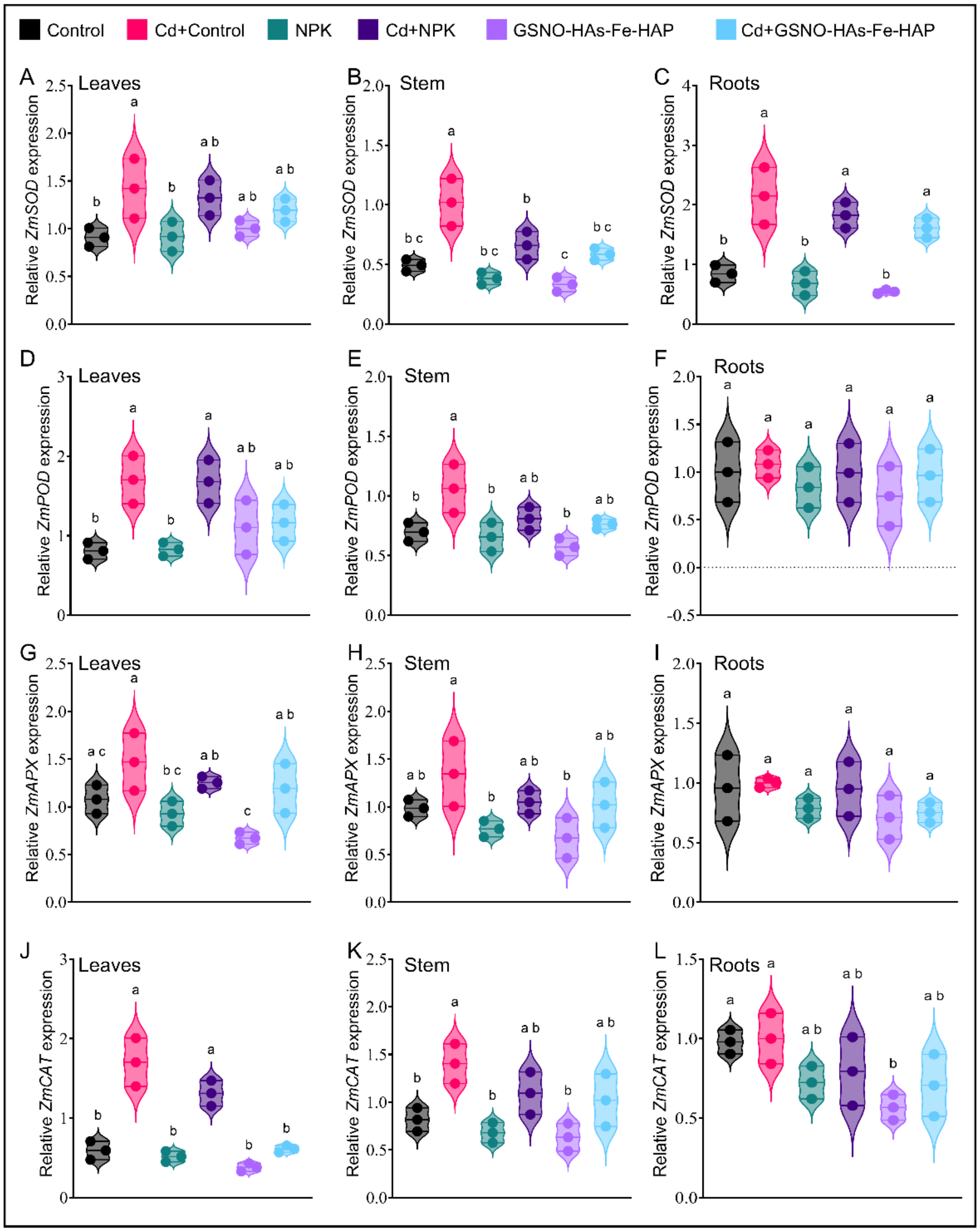

Supplementary Figure 5. Impact of GSNO-HAs-Fe-HAP NPs on the expression of antioxidant genes under Cd stress.

Effects of GSNO-HAs-Fe-HAP NPS on the relative expression of antioxidant genes were determined via qPCR analysis of *ZmSOD* (A–C), *ZmPOD* (D–F), *ZmAPX* (G–I), and *ZmCAT* (J–L) in the leaves, stem, and roots of maize seedlings after 2 weeks of exposure to Cd toxicity. Data are presented as mean ± standard deviation. Different letters indicate significant differences at $p < 0.05$, as determined using one-way ANOVA followed by Tukey's post hoc test.

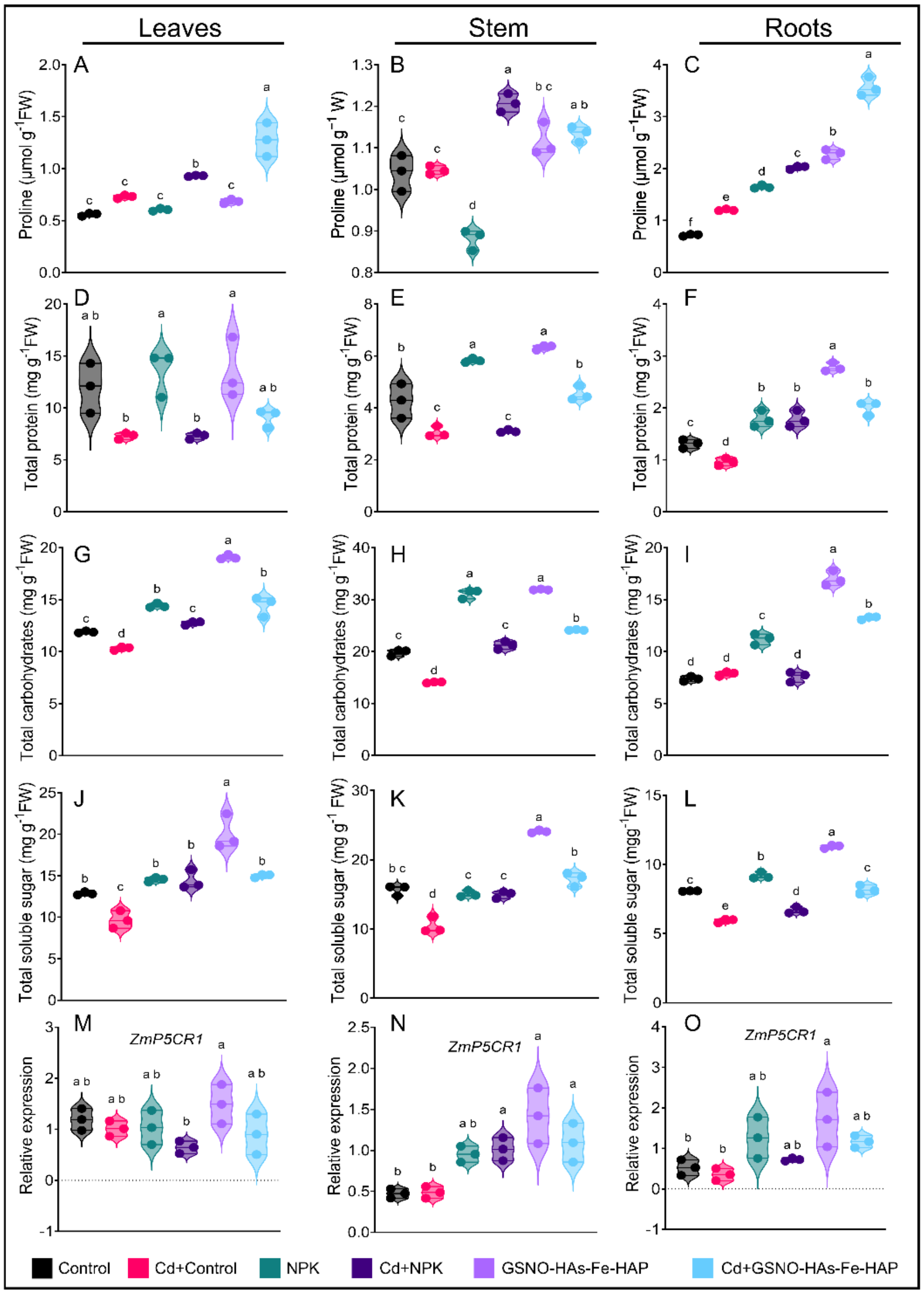

Supplementary Figure 6. Impact of GSNO-HAs-Fe-HAP NPs in osmoprotection against Cd stress.

Effects of GSNO-HAs-Fe-HAP NPs on the accumulation of key osmoprotectants, including proline (A–C), total protein (D–F), total carbohydrate (G–I), total soluble sugar (J–L), were measured in maize seedlings exposed to Cd toxicity for 2 weeks. Furthermore, changes in the relative expression of the proline biosynthesis gene *ZmPCR1* were measured via real-time PCR (M-O). Data are presented as mean ± standard deviation. Different letters indicate significant differences at $p < 0.05$, as determined using one-way ANOVA followed by Tukey's post hoc test.

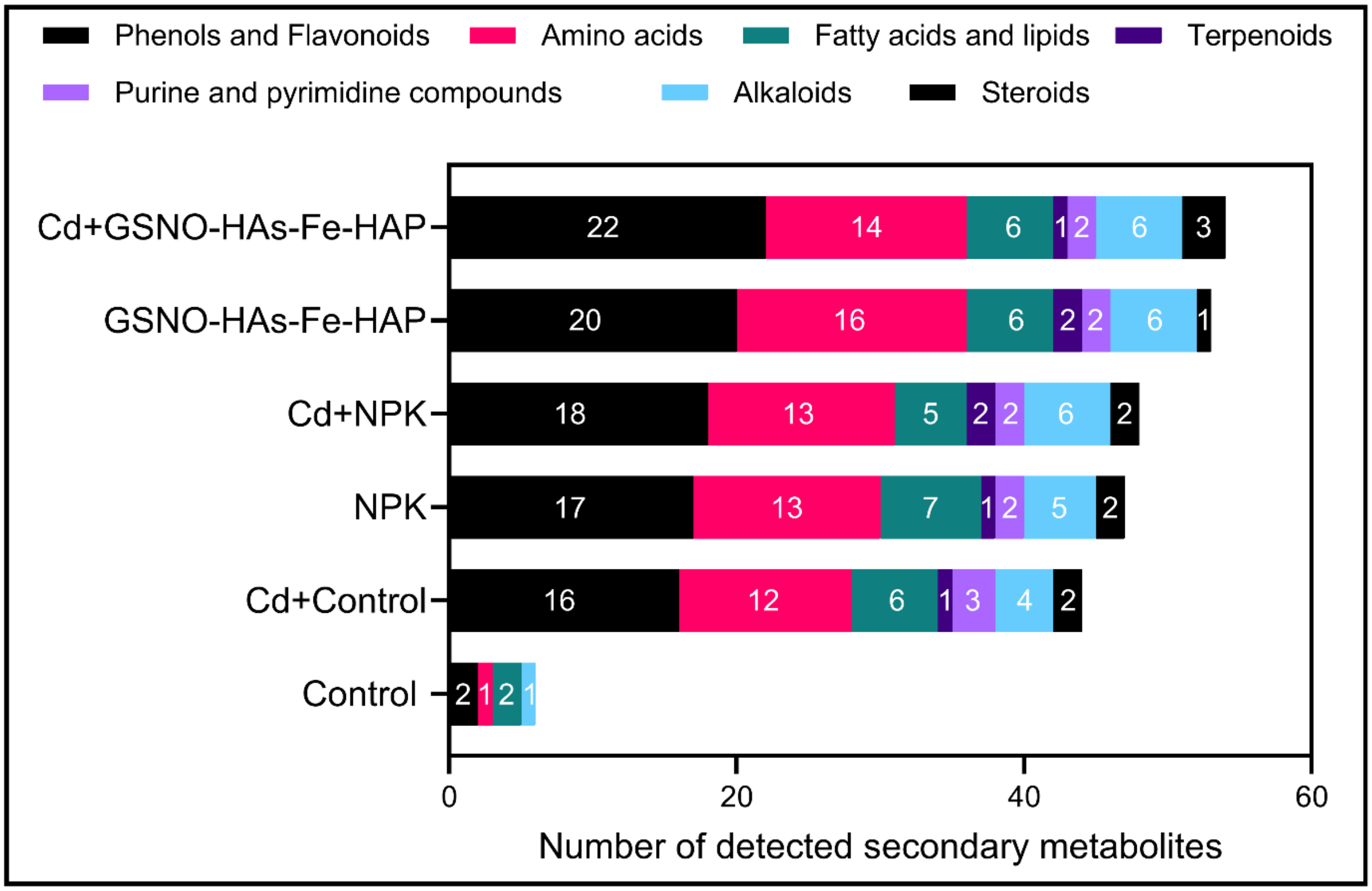

Supplementary Figure 7. Impact of GSNO-HAs-Fe-HAP NPs on the secondary metabolite profile of maize seedlings under Cd stress.

Effects of GSNO-HAs-Fe-HAP NPs on the total number of secondary metabolites in maize seedlings exposed to Cd toxicity for 2 weeks. Data from five individual maize seedlings (n = 5) are presented.

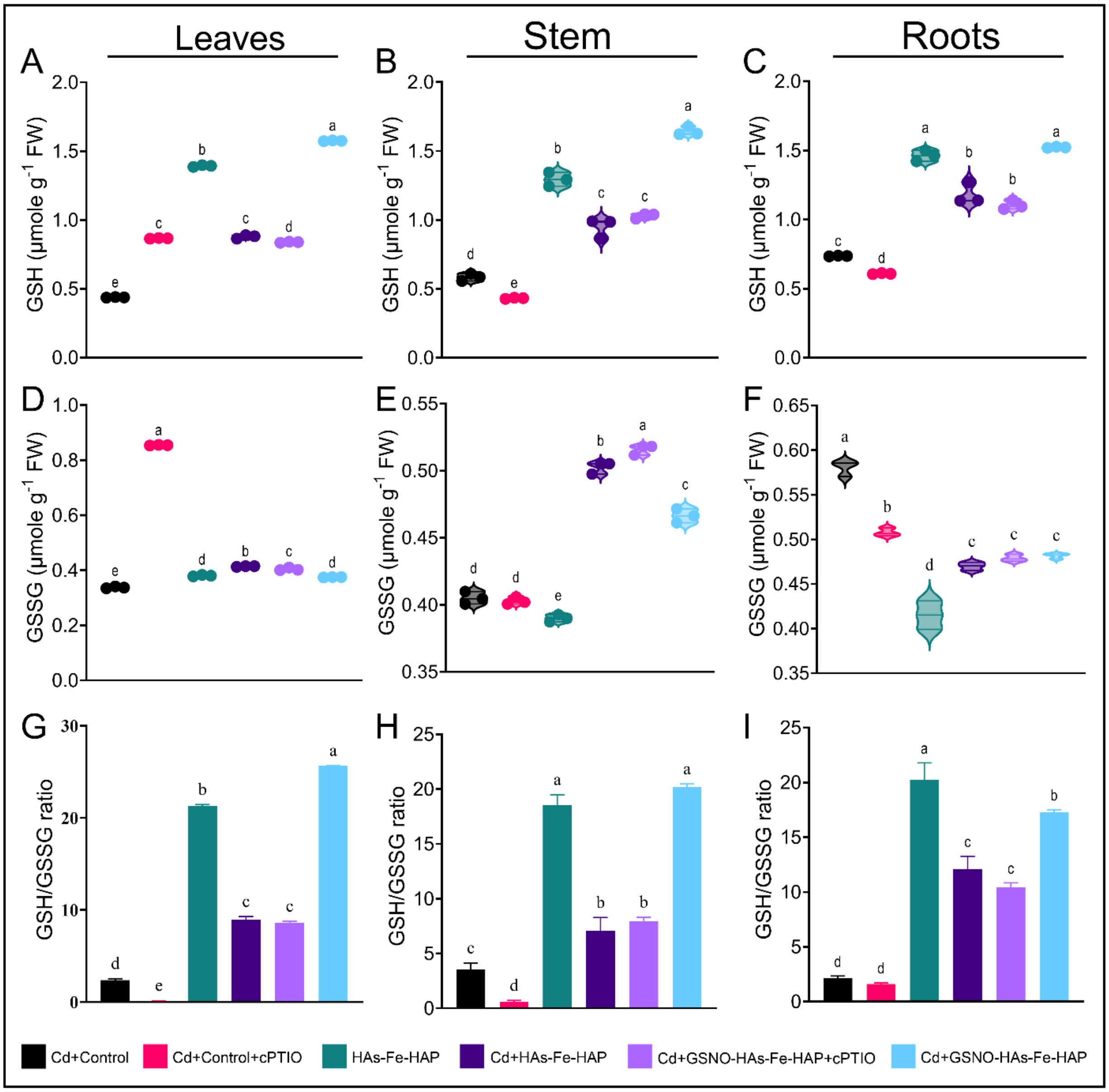

Supplementary Figure 8. Impact of GSNO-HAs-Fe-HAP NPs on cellular oxidized and reduced glutathione.

Impact of GSNO-HAs-Fe-HAP NPs on the cellular redox environment was determined with and without the NO scavenger cPTIO by quantifying GSH (A-C), GSSG (D-F), and GSH/GSSG ratio (G-I) in the leaves, stem, and root tissues. Data are presented as mean ± standard deviation. Different letters indicate significant differences at $p < 0.05$, as determined using one-way ANOVA followed by Tukey's post hoc test.

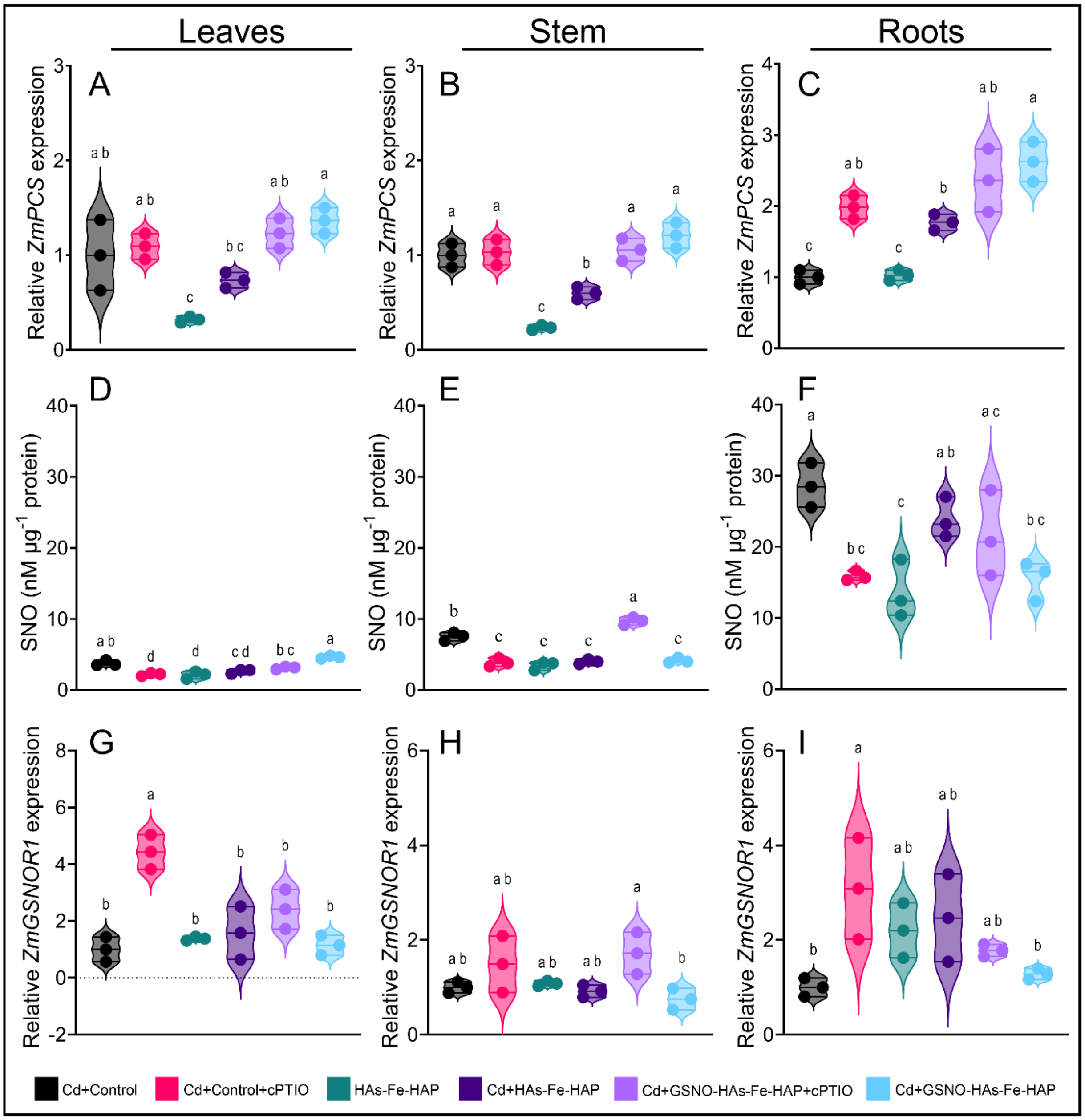

Supplementary Figure 9. Impact of GSNO-HAs-Fe-HAP NPs on gene expression and global SNOs under Cd stress.

Impact of GSNO-HAs-Fe-HAP NPs on the transcription of key genes and global S-nitrosothiol levels in the absence or presence of the NO scavenger cPTIO was determined by quantifying changes in; expression of *ZmPCS* (A-C), SNO contents (D-F), and expression of *GSNOR1* (G-I) in the leaf, stem and root tissues of maize plants. Data are presented as mean ± standard deviation. Different letters indicate significant differences at $p < 0.05$, as determined using one-way ANOVA followed by Tukey's post hoc test.

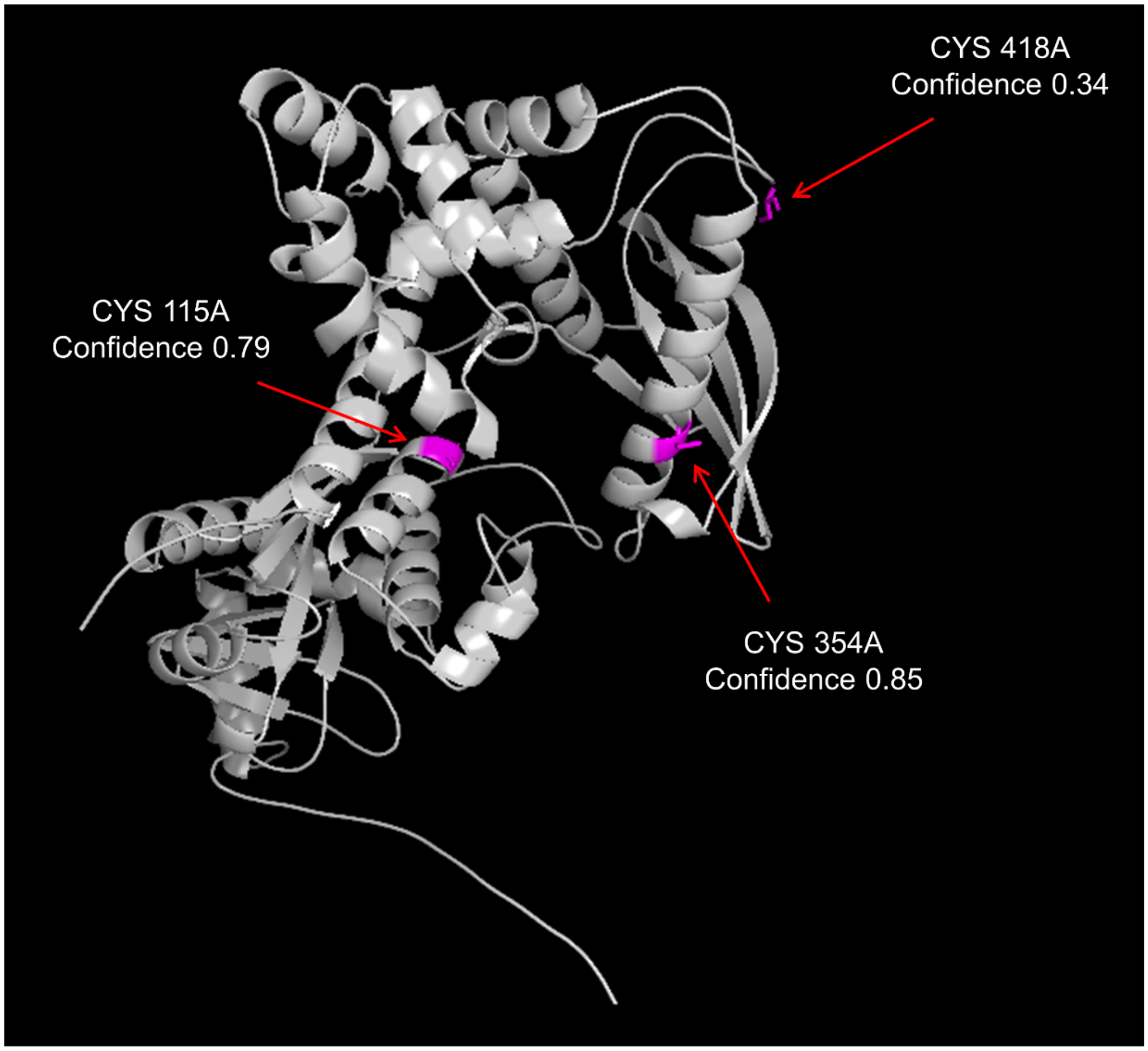

Residue position	Peptide sequence	Total SASA	Side chain SASA	Score	Cutoff	Cluster
115	KVACLAHCSGADVQS	59.18	21.54	2.717	2.443	Cluster B
354	RIAAVVCCQAAMLS	20.37	20.34	2.924	2.443	Cluster B
418	SSPGASSCNSNLKSN	108.87	64.99	2.543	2.443	Cluster B

Analysis threshold: High. Total number of Cys residues = 21 GPS-SNO 1.0 & FreeSASA

Supplementary Figure 10. Prediction of GSNO-HAs-Fe-HAP NPs mediating S-Nitrosylation of ZmPCS.

GSNO-HAs-Fe-HAP mediated S-nitrosylation of ZmPCS was predicted via GPS-SNO1.0 and FreeSASA. Three cysteine residues in ZmPCS—Cys115, Cys354, and Cys418—with a high threshold. FreeSASA-based solvent-accessible surface area calculations of the three cysteine residues exhibit high side-chain SASA values, indicating thiol exposure and a significantly higher chance for NO-mediated modification.

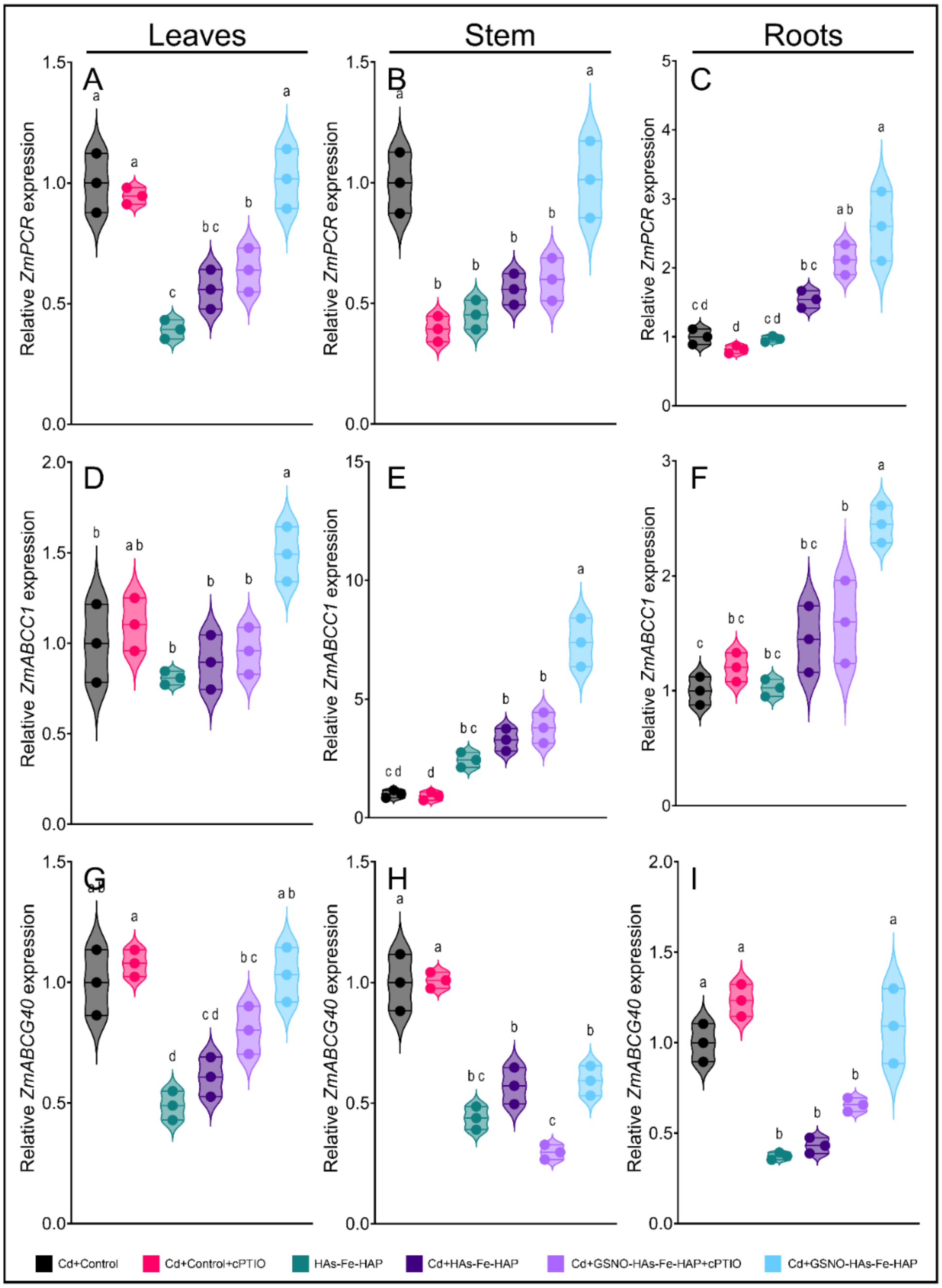

Supplementary Figure 11. Impact of GSNO-HAs-Fe-HAP NPs on gene expression under Cd stress.

Impact of GSNO-HAs-Fe-HAP NPs on the transcription of key genes in the absence or presence of the NO scavenger cPTIO under Cd stress was determined by quantifying changes in the expression of *ZmPCR* (A-C), *ZmABCC1* (D-F) and *ZmBCG40* (G-I) in the leaf, stem and root tissues of maize plants. Data are presented as mean ± standard deviation. Different letters indicate significant differences at $p < 0.05$, as determined using one-way ANOVA followed by Tukey's post hoc test.

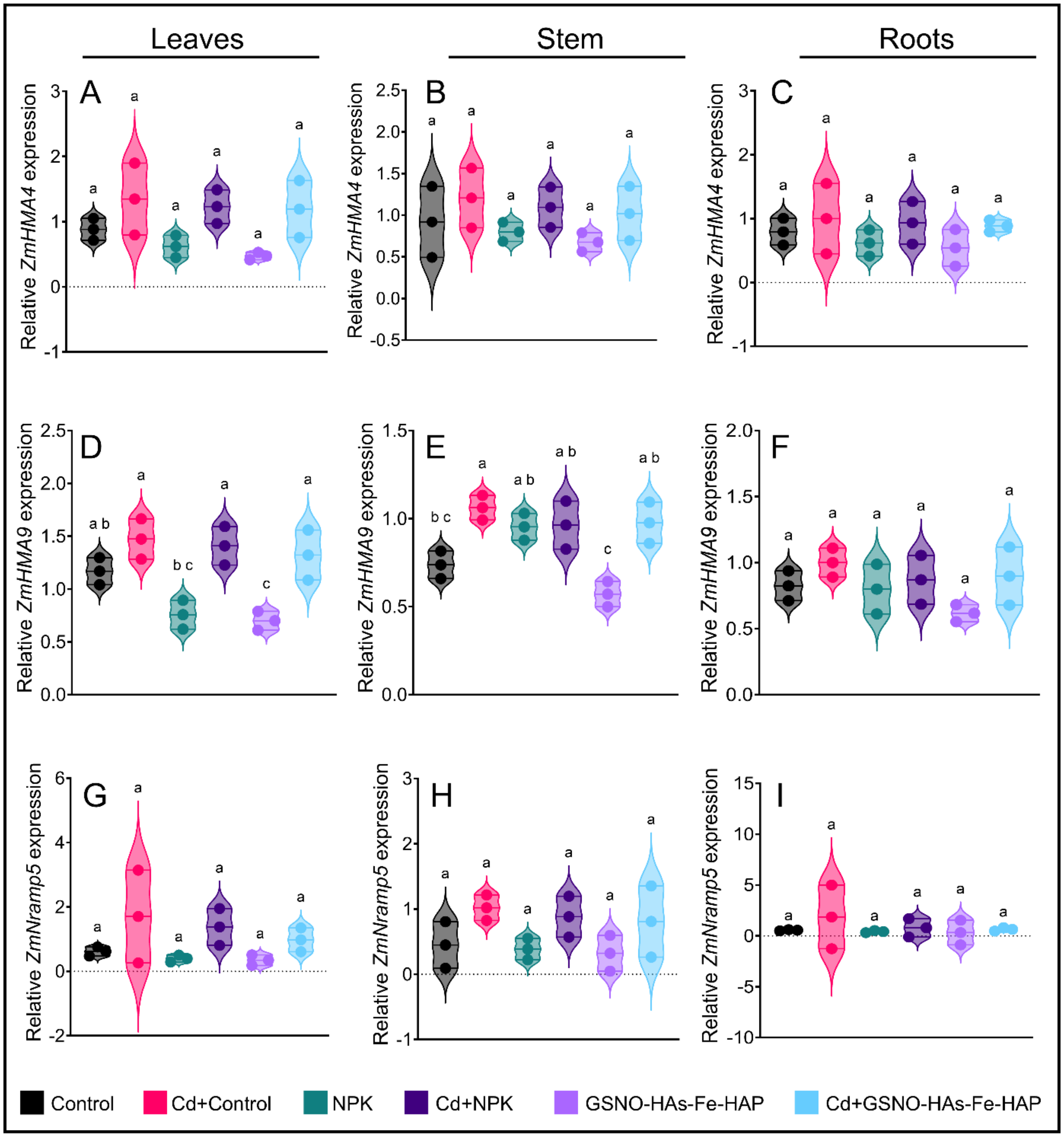

Supplementary Figure 12. Impact of GSNO-HAs-Fe-HAP NPs on the transcription of metal ion transporters under Cd stress.

Effects of GSNO-HAs-Fe-HAP NPs on metal ion transport were determined by measuring the changes in the expression of metal transporter genes *ZmHMA4* (A–C), *ZmHMA9* (D–F), and *ZmNramp5* (G–I) in the leaf, stem, and root tissues of maize seedlings exposed to Cd toxicity for 2 weeks. Data are presented as mean ± standard deviation. Different letters indicate significant differences at $p < 0.05$, as determined using one-way ANOVA followed by Tukey's post hoc test.

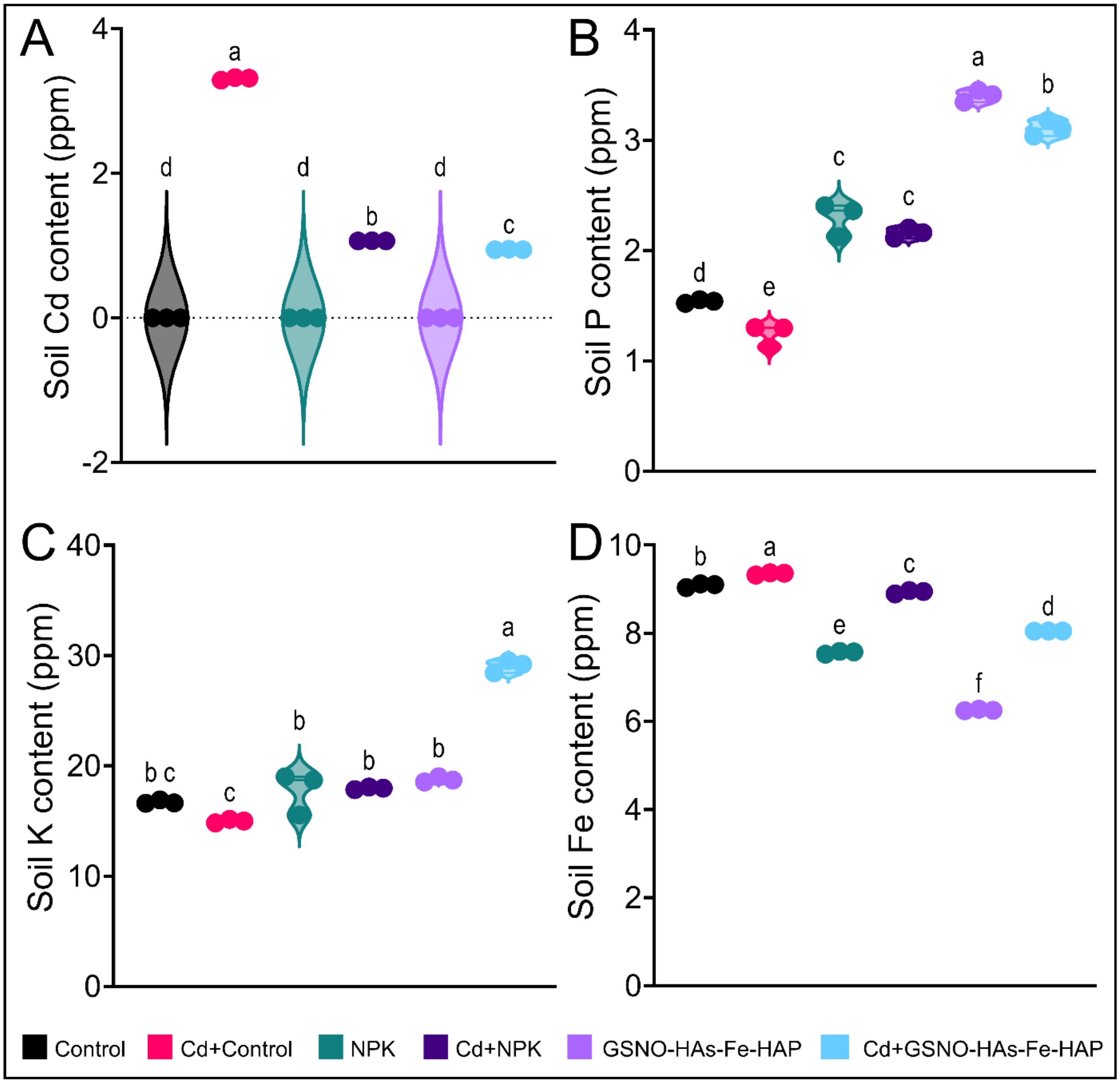

Supplementary Figure 13. Impact of GSNO-HAs-Fe-HAP NPs on grain Cd and soil P, K, and Fe contents.
The long-term Impact of GSNO-HAs-Fe-HAP NPs was determined by quantifying grain Cd (A), soil phosphorus (B), soil potassium (C), and soil iron (D) contents after harvesting the plants. Data are presented as mean ± standard deviation. Different letters indicate significant differences at $p < 0.05$, as determined using one-way ANOVA followed by Tukey's post hoc test.

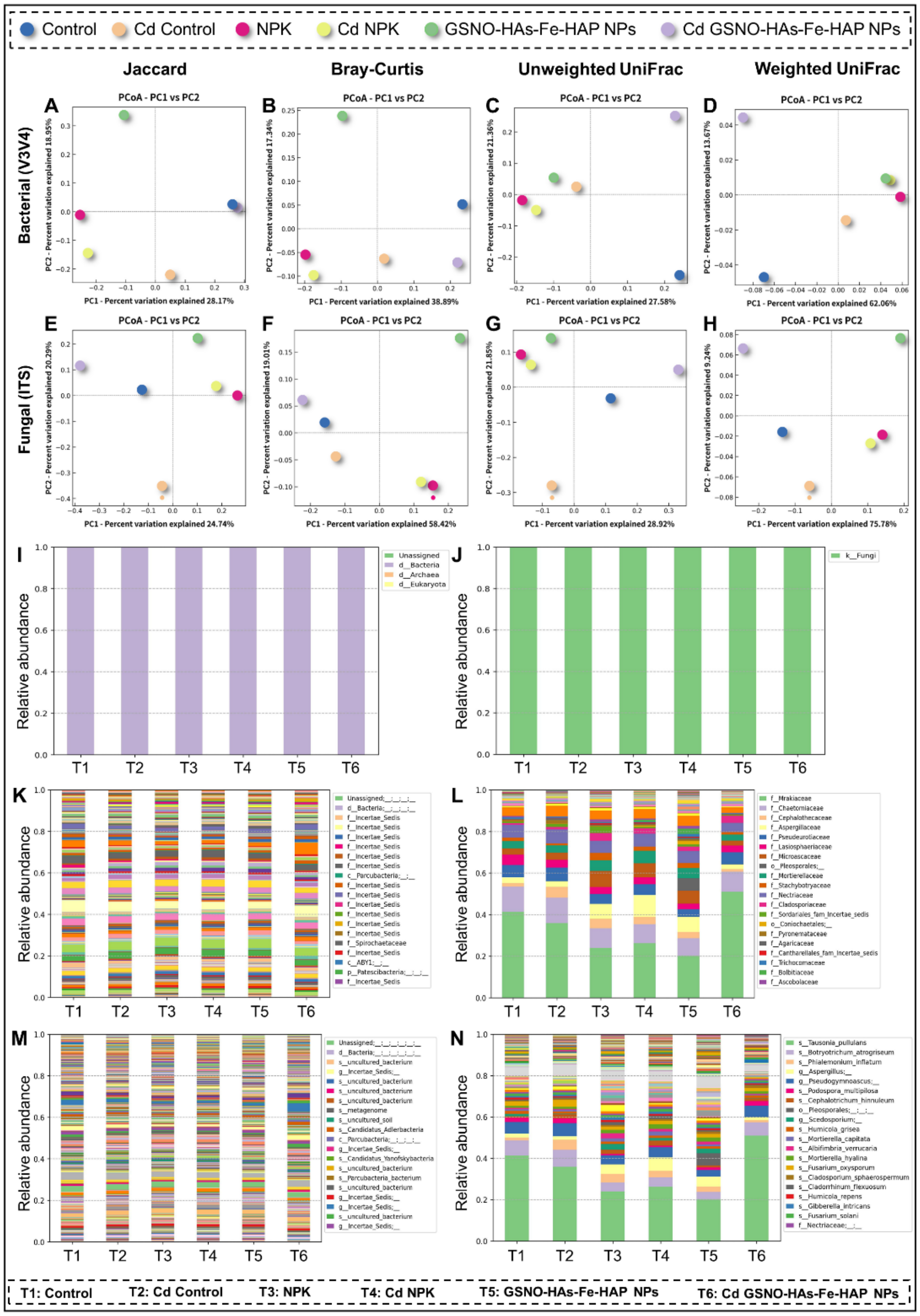

Supplementary Figure 14. Beta diversity and Hierarchical taxonomic profiling of soil rhizosphere microbiome following GSNO-HAs-Fe-HAP NPs application.

Beta diversity analysis unveiled abundance-driven and phylogenetically distinct shifts in the fungal microbiome. Principal Coordinate Analysis (PCoA) plots illustrating structural differences in bacterial (V3V4, A–D) and fungal (ITS, E–H) communities based on four distinct distance metrics to decouple ecological drivers. (A–D) Bacterial Resilience: Bacterial communities show stochastic distribution without distinct clustering across Jaccard (A), Bray-Curtis (B), Unweighted UniFrac (C), and Weighted UniFrac (D)

metrics. This confirms the structural resilience and homeostasis of the bacteriome under stress. (E–H) Fungal Remodeling: Fungal communities reveal a mechanism-specific shift. (E, G) Qualitative metrics (Jaccard, e; Unweighted UniFrac, G) explain relatively low variance (PC1 < 29%), indicating minimal species turnover. (F, H) Quantitative metrics (Bray-Curtis, F; Weighted UniFrac, H) explain substantially higher variance (PC1 > 58% and 75%, respectively). The massive separation in Weighted UniFrac (H) confirms that the microbiome shift in the Cd-GSNO-HAs-Fe-HAP NPs group is driven by the selective proliferation (bloom) of a phylogenetically distinct lineage (*Tausonia*) rather than the introduction of novel species. Hierarchical taxonomic profiling confirmed bacterial stability and species-level fungal dominance. Relative abundance of bacterial and fungal communities classified at the Domain (I, J), Family (K, L), and Species (M, N) levels across six experimental groups. (I, K, M) Bacterial Stability: The bacterial community exhibits remarkable structural consistency across all taxonomic ranks. From the Domain down to the Species level, no single taxon dominates or shifts significantly in response to Cd stress or GSNO-HAs-Fe-HAP NPs treatment, reinforcing the ecological safety and homeostasis of the bacteriome. (J, L, N) Fungal Shift: In contrast, fungal communities display a specific reconfiguration in the Cd+GSNO-HAs-Fe-HAP NPs group. This shift is driven by the explosive expansion of the family *Mrakiaceae* (L) and is identified at the species level specifically as *Tausonia pullulans* (N). This species-level resolution confirms that the "Tausonia bloom" described in the main text is attributed to a single, dominant species.

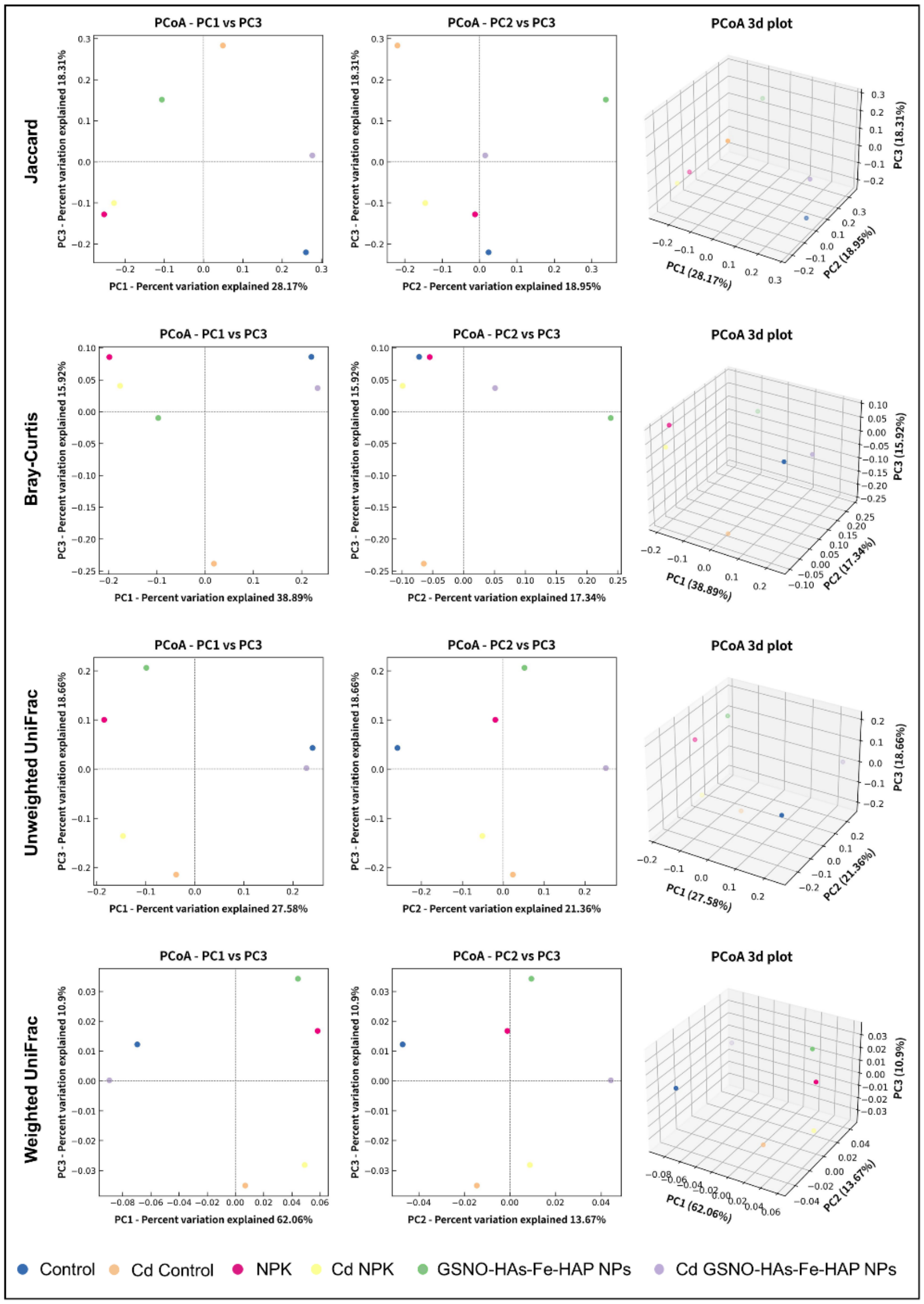

Supplementary Figure 15. Multidimensional beta diversity profiling corroborates the structural resilience of the bacterial microbiome.

Extended Principal Coordinate Analysis (PCoA) plots of bacterial communities (16S rRNA V3-V4) visualized along alternative axes (PC1 vs. PC3, PC2 vs. PC3) and in three-dimensional (3D) space using four distinct distance metrics: Jaccard, Bray-Curtis, Unweighted UniFrac, and Weighted UniFrac. Consistent with the primary PC1 vs. PC2 analysis. These extended visualizations reveal a stochastic distribution of samples without treatment-specific clustering across all dimensions. This lack of separation serves as robust evidence that GSNO-HAs-Fe-HAP NPs treatment maintains the homeostasis of the indigenous bacteriome and does not induce ecological dysbiosis, regardless of the metric or dimensional view applied.

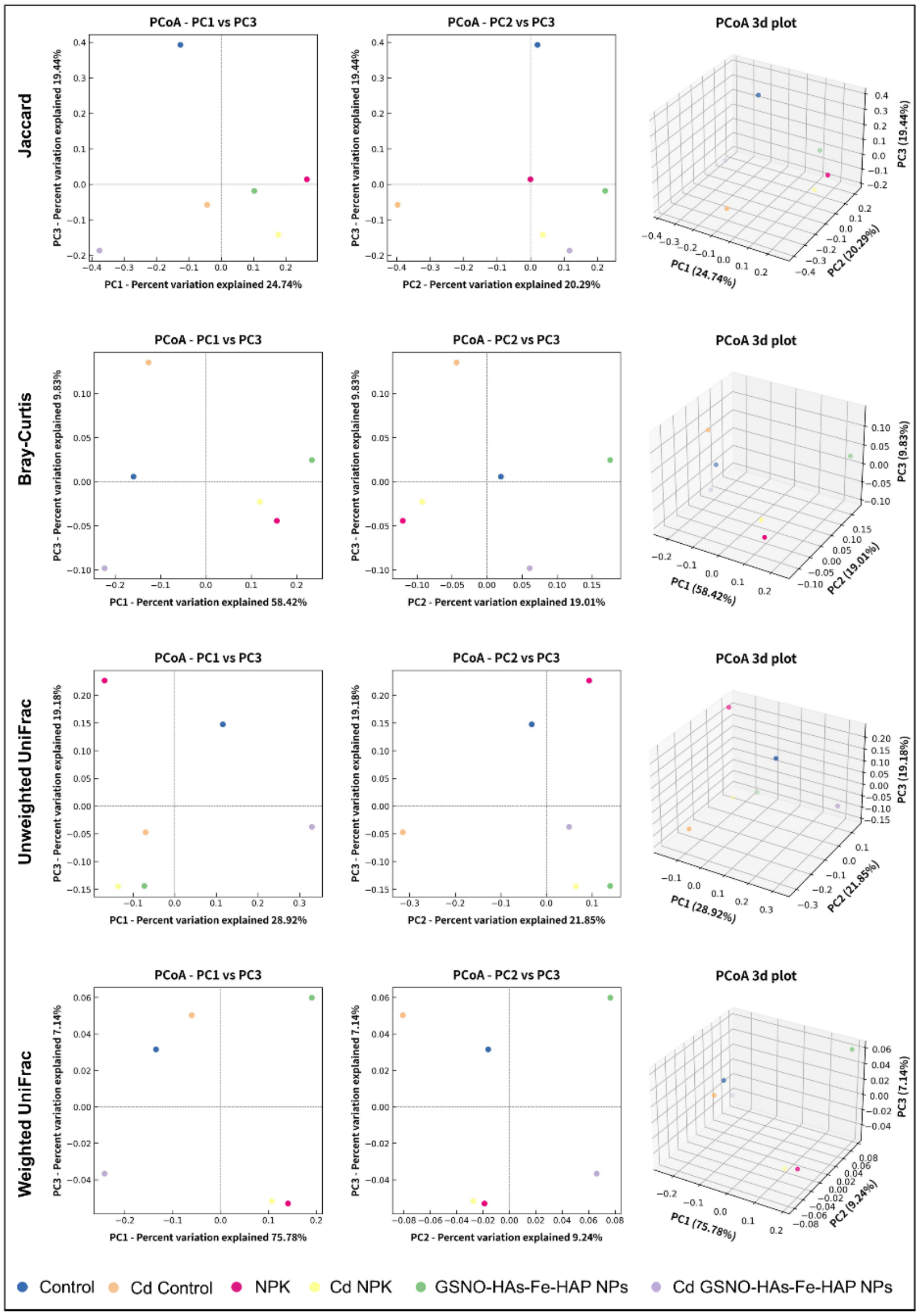

Supplementary Figure 16. Extended beta diversity analysis of fungal communities reveals abundance-driven divergence.

Extended PCoA plots of fungal communities (ITS2) visualized along alternative axes (PC1 vs. PC3, PC2 vs. PC3) and in 3D space using Jaccard, Bray-Curtis, Unweighted UniFrac, and Weighted UniFrac metrics. (Top & Third rows) Qualitative metrics (Jaccard, Unweighted UniFrac) continue to show weak separation even in higher dimensions, indicating minimal species turnover. (Second & Bottom rows) Quantitative metrics (Bray-Curtis, Weighted UniFrac) exhibit distinct clustering of the Cd+GSNO-HAs-Fe-HAP NPs group away from other treatments. The persistence of this separation in 3D space confirms that the community shift is robust and driven by the dominant proliferation of phylogenetically distinct taxa (*Tausonia*) rather than random variation.

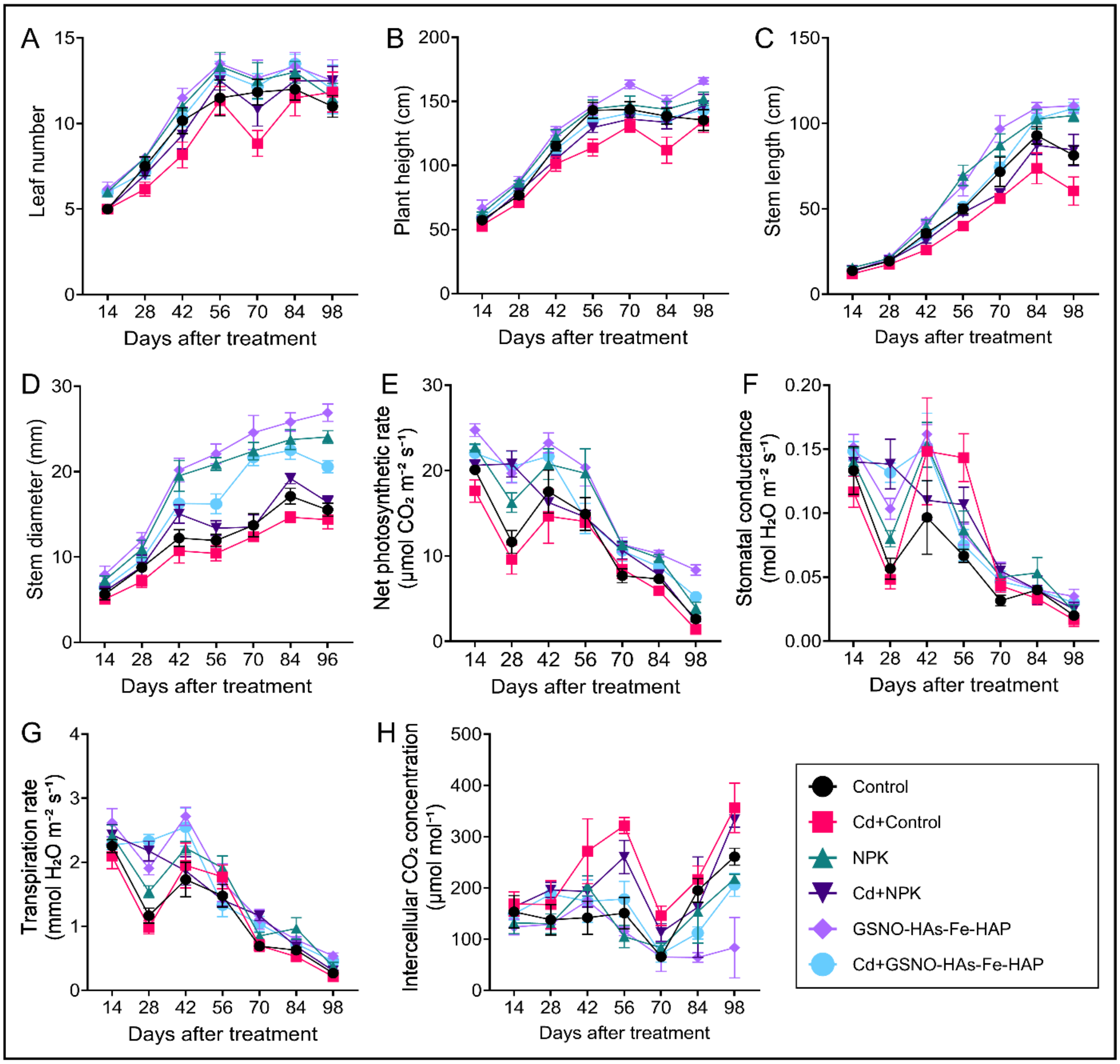

Supplementary Figure 17. Impact of GSNO-HAs-Fe-HAP NPs on grain Cd and soil P, K, and Fe contents.

The long-term impact of GSNO-HAs-Fe-HAP NP application was determined by analyzing the cadmium tolerance index of the plant shoot and root tissues (A, B), and key growth metrics including leaf number (C), plant height (D), stem length and diameter (E, F), net photosynthetic rate (G), stomatal conductance (H), transpiration rate (I), and intercellular CO_2 (J), each measured for up to 98 days. Data are presented as mean ± standard deviation. Different letters indicate significant differences at $p < 0.05$, as determined using one-way ANOVA followed by Tukey's post hoc test.

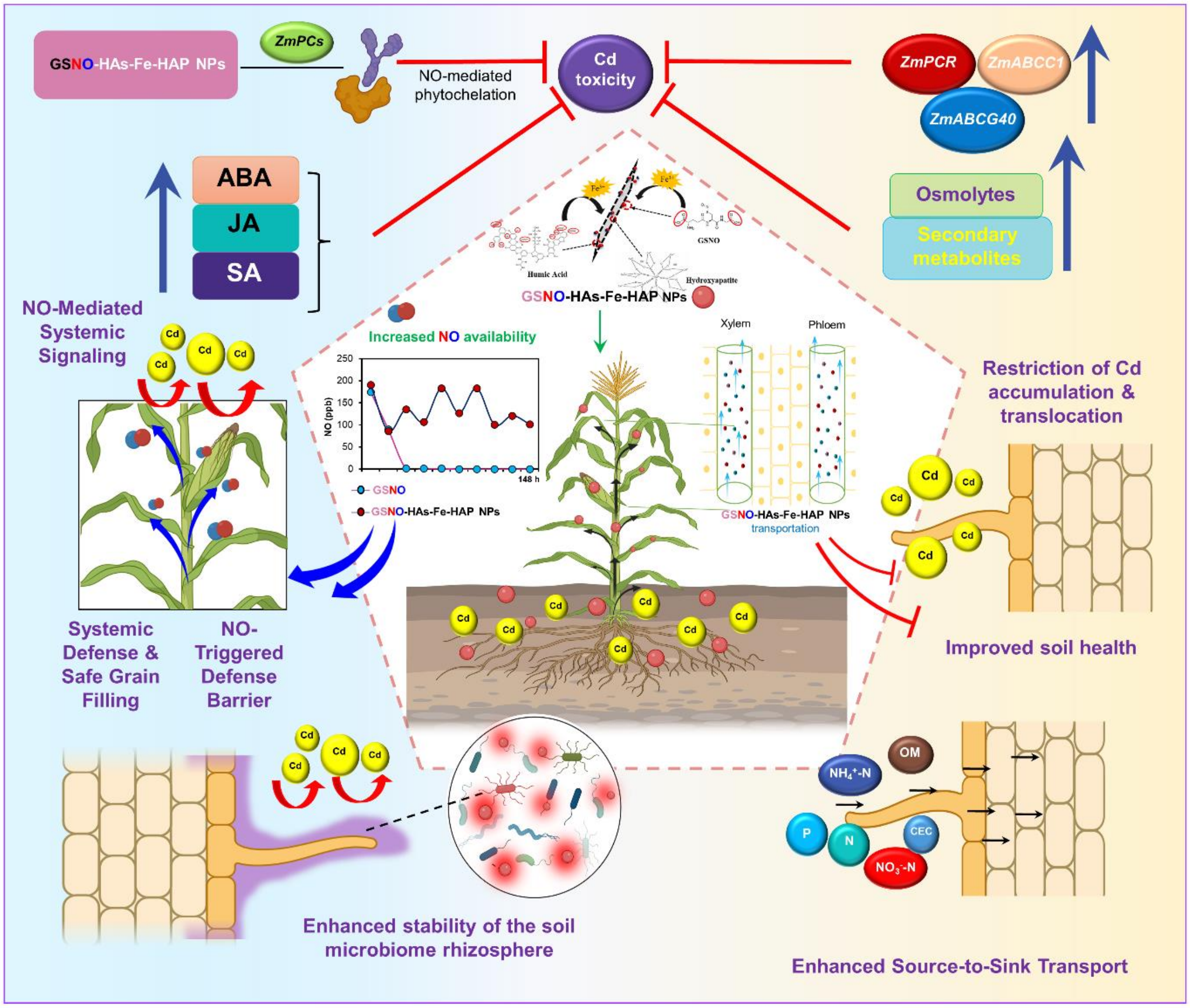

Supplementary Figure 18. Proposed model showing the effects of GSNO-HAs-Fe-HAP nanoparticle composite under cadmium stress.

The accumulation of GSNO-HAs-Fe-HAP NPs in maize seedlings effectively reduces the Cd uptake by limiting its translocation and bioavailability. The sustained release of NO from GSNO-HAs-Fe-HAP NPs enhances plant metabolic functions under Cd stress. Notably, NO-mediated phytochelation and the upregulation of metal transporter genes enhanced the Cd detoxification and tolerance. The nanocarrier system GSNO-HAs-Fe-HAP NPs delivers essential nutrients, improving soil fertility, enhancing and stabilizing the soil microbial community at the root interface, source to sink transport, and supporting overall plant health.

Supplementary Table 1. List of primers and their sequence

Name	Sequence (5' – 3')	Length (bp)	Tm (℃)
*ZmGSNOR1*_F	TAACTGATGTGCAGCCTGGG	20	58.7
*ZmGSNOR1*_R	ACTCCGGACCTTACCACAGA	20	58.8
*ZmPAL*_F	AAGGAGAAGAGGAGGGAGGG	20	59.5
*ZmPAL*_R	GAAGAAAGAGCAACGCCACA	20	57.1
*ZmPCs*_F	TGTTCAGCTTCAACTACAAACCA	23	54.9
*ZmPCs*_R	CCAGAACATTGCCGTATCATT	21	53.6
*ZmPOD*_F	TGGGATTCTACGACACCAGC	20	58.1
*ZmPOD*_R	CCTGACAAAGCAGTCGTGGA	20	58.7
*ZmSOD*_F	CTGGGCCACACTTCAATCCT	20	58.8
*ZmSOD*_R	AACACCATCTTCTCCCGCTG	20	58.6
*ZmAPX*_F	CGTCATCAGTGTTGGTCAATGT	22	56.1
*ZmAPX*_R	AGAACCCTTGGTGGCATCAG	20	58.8
*ZmCAT*_F	ACATGTTCTCCTTCCTCTTCGAC	23	57.2
*ZmCAT*_R	CTGTGGTTGGCGTAGGTGAC	20	59.9
*ZmABCC3*_F	GCCAACCATGTTCGAGGGTA	20	58.7
*ZmABCC3*_R	CACCTCCTCACCCAACTGAC	20	59.3
*ZmHMA4*_F	GAGTCCCAGGCTACATCGC	20	59.7
*ZmHMA4*_R	ATCAGCCTCTGAGTTCGGGA	20	59.2
*ZmHMA9*_F	GTCTCGGTGGACCTGGAATG	20	59.3
*ZmHMA9*_R	CTCGTCCAAGTCGATGTCGT	20	58.1
*ZmP5CR*_F	AACCTGGCCTTCGCGTC	17	60.3

*ZmP5CR*_R	CGATCTGAGGCTTCACGGAG	20	59.2
*ZmNramp5*_F	GACGGTGCCGTGGACTC	17	61
*ZmNramp5*_R	ACACGATGACCTTCTCAGCC	20	58.9
*ZmABCG*_F	TACTGGATTTGCCCACTGGC	20	59
*ZmABCG*_R	TCTGCTCACTTTCTCCAGGG	20	57.8
*ZmPCR*_F	CATCACGTTCGGGCAGACC	20	60.6
*ZmPCR*_R	GGAGTAGACGCACTGACAGC	20	59.4
*ZmABCC1*_F	TGGTCTGTCCACAATCCGTG	20	58.5
*ZmABCC1*_R	GATGCCACCCAATGTCTCCA	20	58.8
ZmActin1-F	GGGATTGCCGATCGTATGAG	20	56.8
ZmActin1-R	GAGCCACCGATCCAGACACT	20	59.6

Supplementary Table 2. Elemental contents of C and H in different particle samples, expressed as weight percentages (%), as determined by elemental analysis.

**Sample**	**C (%)**	**H (%)**
Bare HAP	ND	0.07
GSNO-HAP	ND	0.08
GSNO-HAs-HAP	1.01	0.09
GSNO-Fe-HAP	1.81	0.32
GSNO-HAs-Fe-HAP	3.43	0.36

Bare HAP, hydroxyapatite; GSNO-HAP, hydroxyapatite coated with GSNO; GSNO-HAs-HAP, hydroxyapatite coated with GSNO and humic acid; GSNO-Fe-HAP, hydroxyapatite coated with GSNO and ferric ions; GSNO-HAs-Fe-HAP, hydroxyapatite coated with humic acid, GSNO, and ferric ions.

Supplementary Table 3. Organic and inorganic parts calculated from TGA (Fig. 1B), dry weight (Fig. 1C), and ICP-OES of bare HAP, GSNO-HAP, GSNO-HAs-HAP, GSNO-Fe-HAP, and GSNO-HAs-Fe-HAP

Sample	H_2O (mg)	Total organic matter (mg)	Total Inorganic matter (mg)	GSNO (mg)	HAs (mg)	Fe (mg)	HAP (mg)
Bare HAP	25.7	18.6	955.7	0	0	0	955.7
GSNO-HAP	30.2	36.4	909.1	3.9	0	0	909.1
GSNO-HAs-HAP	24.6	50.8	774	2.5	48.3	0	774
GSNO-Fe-HAP	91.7	59.3	1013.7	59.3	0	102.3	911.4
GSNO-HAs-Fe-HAP	139.9	214.3	1095.8	85.7	128.6	143.7	952.1

Bare HAP, hydroxyapatite; GSNO-HAP, hydroxyapatite coated with GSNO; GSNO-Fe-HAP, hydroxyapatite coated with ferric ions and GSNO; GSNO-HAs-HAP, hydroxyapatite coated with humic acid and GSNO; GSNO-HAs-Fe-HAP, hydroxyapatite coated with humic acids, ferric ions, and GSNO. The contents of H_2O, total organic matter, and inorganic matter were estimated based on the dry weight and weight changes in the TGA profile over the temperature ranges of room temperature–200 °C, 200–600 °C, and 600–1,000 °C, respectively (Figure 1B and 1C). The Fe and S contents were quantified by ICP-OES after acid digestion of the particles. The GSNO content was calculated from its molecular formula and the S content, whereas the remaining organic fraction, excluding GSNO, was assigned to HAs.

Supplementary Text 1. TGA, FT-IR, and elemental analysis

Approximately 10 mg of each sample was used for TGA (Q600, TA Instruments, USA) analysis, with a heating rate of 10°C min^{-1} up to 1000°C under air conditions. Attenuated total reflection mode was employed to obtain FT-IR spectra of each product in the scan range of 400 to 4000 cm^{-1}. The freeze-dried GSNO-Fe-HAP and GSNO-HAs-Fe-HAP particles (0.5 g each) were used to get the approximated elemental composition (i.e., carbon, hydrogen), which was performed by an elemental analyzer (Vario-Micro Cube, Elementar Analysensysteme GmbH, Germany).

The nanoparticle composites (450 mg each) were digested in a mixture of 70% HNO_3 (7.5 mL) and 36% HCl (22.5 mL) at a volume ratio of 1:3 (v/v) at 200 °C for 5 h. After complete digestion, the resulting solutions were diluted with deionized water to a final volume of 100 mL and filtered through filter paper to remove any insoluble residues. The concentrations of Ca, P, Fe, and S were quantified by inductively coupled plasma-optical emission spectrometry (ICP-OES; OPTIMA 5300DV, Perkin-Elmer Instruments, USA).

Supplementary Text 2. Zeta potential, hydrodynamic size, and BET analyses

A dynamic light scattering spectrophotometer (ELS 8000, Otsuka, Japan) was utilized to evaluate the hydrodynamic size and Zeta potentials of bare HAP and GSNO-HAs-Fe-HAP. To measure surface BET surface areas, both bare HAP and GSNO-HAs-Fe-HAP powders were placed in a vacuum, where the temperature was held at 130 ℃ for 12 h. The surface areas of each sample were calculated by nitrogen adsorption-desorption isotherm (ASAP, Micromeritics).

Supplementary Text 3. Organic acid responsiveness, release behavior, and calculation of organic and inorganic parts

The GSNO-HAs-Fe-HAP powders (0.5 g) were highly suspended in deionized water (15 mL), and the pH of the water containing suspended particles was adjusted to 4.5 by adding either 1 N HCl or 1 M citric acid. The samples were placed on a roller mixer (60 rpm) and incubated at room temperature for 1 day, followed by centrifugation (4000 rpm, 10 min). Next, the supernatants were separated, and fresh water (15 mL) was

added daily to each tube to prevent saturation of soluble materials. The pH of the water containing suspended particles was adjusted to 4.5, as described above. Subsequently, an aliquot (5 mL) of the harvested supernatant was further subjected to acid digestion (7.5 mL of HNO_3 and 22.5 mL of 36% HCl, 1:3 v/v ratio; 200 ℃ for 3 h). Released Ca, P, and Fe content were then quantitatively measured by ICP-OES. Released HAs were qualitatively assessed by measuring the absorbance at 450 nm before the acid digestion.

Supplementary Text 4. SEC

GSNO-HAs-Fe-HAP powders (200 mg) were highly dispersed in distilled water (5 mL) and placed on a roller (60 rpm) at room temperature for 2 hours. An aliquot (1.2 mL) was taken and centrifuged (13 000 rpm, 5 min) to obtain released HAs. Thereafter, the supernatants (1 mL) were taken, followed by syringe filtration (0.45 μm, PTFE, Advantec). Commercial HAs powder (10 mg) was dissolved in 1 mL of phosphate buffer solution (100 mM NaH_2PO_4 at pH 6.5) containing sodium azide (4.6 mM NaN3), followed by syringe filtration (0.45 μm, PTFE, Advantec). The phosphate buffer containing sodium azide was used as the mobile phase at a flow rate of 0.6 mL min^{-1}, and the absorbance at 280 nm and 450 nm was monitored. Poly(styrene sulfonate) showing 6 520, 14 900, and 145 000 *M*w (Da) and ferulic acid were employed to make a calibration curve.

Supplementary Text 5. HPLC-ESI-Q-TOF MS

0.5 mM GSNO solution (1 mL) was syringe-filtered (0.45 μm), while GSNO-HAs-Fe-HAP powders (200 mg) were highly suspended in deionized water (5 mL), followed by incubation on a roller mixer (60 rpm) for 2 hours. The supernatants (1 mL) were harvested after centrifugation (13 000 rpm, 5 min) and syringe-filtered (0.45 μm) before analysis. Standard GSNO solution and the supernatants containing detached GSNO from the particles were analyzed via HPLC (SCL-40, Shimadzu, Japan) equipped with an ESI-Q-TOF MS (X500R, AB SCIEX, USA), where an XBridge® Amide column (2.1 × 150 mm^2, Waters) was equipped. The mobile phase flow rate and column temperature were constantly maintained at 0.3 mL min-1 and 25 ℃, respectively, and the mobile phase was composed of 0.1% formic acid in water and 0.1% formic acid in acetonitrile

at a 7:3 (v/v) ratio. The eluted samples were ionized based on negative ESI mode, and subsequent MS/MS spectra were obtained using Information-dependent acquisition (IDA) mode.

Supplementary Text 6. Transmission and Scanning Electron Microscopy Analysis of GSNO-HAs-Fe-HAP NPs

The nanostructure of GSNO-HAs-Fe-HAP NPs was examined using Transmission Electron Microscopy (TEM, Hitachi, HT7700) at an accelerating voltage of 120.0 kV. TEM samples were prepared by coating a fine-sonicated dilute suspension of the sample onto a 200-mesh carbon-coated copper grid, using acetone to minimize aggregation. To analyze the shape of the GSNO-HAs-Fe-HAP NPs, scanning electron microscopy (SEM) analysis was performed using a Hitachi SU8230 instrument at a working distance of 4.4 mm, with a 10 kV voltage and 50,000× magnification. Additionally, Energy Dispersive X-Ray Spectroscopy (EDX) analysis and elemental mapping were performed to determine the elemental composition of the synthesized material using an EDX microanalysis detector (OXFORD, Ultim Max100). Dried HA-GSNO-HAs-Fe-HAP NPs were mounted on a sample holder covered with double-sided carbon tape. To create a conductive surface, carbon-coated sputtering was performed at 20 mA under argon gas for 30 s.

Supplementary Text 7. Measurement of lipid peroxidation and H_2O_2 content

Lipid peroxidation was quantified by measuring the malondialdehyde (MDA) content following the protocol of Dhindsa and Matowe (Dhindsa & Matowe, 1981). Briefly, 100 mg of leaf, stem, and root samples were homogenized in 1.5 mL of 0.1% (w/v) trichloroacetic acid (TCA) and centrifuged at 14,000 rpm for 10 min at 4°C. From the supernatant, two 250 μL aliquots were taken, and one was mixed with 1 mL of 20% (w/v) TCA, while the other was combined with 1 mL of 20% (w/v) TCA and 0.5% (w/v) thiobarbituric acid (TBA). Both mixtures were heated in a hot bath at 95°C for 30 min, cooled, and then centrifuged at 14,000 rpm for 10 min at 4°C. Absorbance was recorded at 532 nm for specific measurements and at 600 nm for non-specific ones. MDA concentration was determined using an extinction coefficient of 155 mM^{-1} cm^{-1} and expressed as micromoles of MDA per gram of fresh weight (FW).

To quantify H_2O_2 content, the potassium iodide reaction method was employed, as detailed by Alexieva et al. (Alexieva et al., 2001). Briefly, 200 mg of the sample was homogenized with 1% (*w*/*v*) trichloroacetic acid on ice and subsequently centrifuged at 13,000 rpm for 15 min. The fresh supernatant was mixed with potassium phosphate buffer and potassium iodide reagents and then incubated in the dark for 1 h. A UV spectrophotometer (Thermo Scientific Multiskan GO Microplate Spectrophotometer, Finland) was used to check the absorbance at 390 nm. Absorbance was measured at 390 nm using a UV spectrophotometer, and H_2O_2 content was determined via a standard curve and expressed as nanomoles per gram of FW.

Supplementary Text 8. Determination of Proline Content, Total Proteins, Total Carbohydrates, and Total Soluble Sugars

Proline content in maize leaves, stems, and roots was measured following the procedure outlined by Bates et al. (Bates et al., 1973). Briefly, fresh tissue samples (100 mg) were individually solubilized in 3% aqueous sulfosalicylic acid. Subsequently, the fresh supernatant was taken in vials, and 2 mL of acid ninhydrin and glacial acetic acid were added. The mixture was vortexed and incubated in a water bath (100°C) for 1 h. After cooling the mixture, 4 mL of toluene was added, and the mixture was vortexed again to separate the toluene phase, which contained the chromophore. The absorbance of the toluene layer was recorded at 520 nm, with toluene serving as the blank. The proline content was expressed as micromoles per gram of FW.

The total soluble protein was extracted from the plant samples using 1× PBS buffer under liquid nitrogen. The extract was centrifuged at 14,000 rpm for 15 min at 4°C, and the supernatant was transferred to a 96-well plate. The protein content was measured at 595 nm using a UV spectrophotometer (Thermo Scientific Multiskan GO Microplate Spectrophotometer, Finland). Bovine serum albumin (BSA) was used as a standard for quantification (Bradford, 1976).

Total carbohydrates were assessed using a colorimetric assay based on the methods described by Dubois (DuBois et al., 1956) and Murphy (Murphy, 1958), with slight modifications. Briefly, the samples (100 mg) were extracted using 1.5 mL of 80% ethanol. After homogenization, the solutions were centrifuged at 14,000 rpm for 15 min

at 4°C, and the supernatant was transferred to a fresh tube. Subsequently, 1.8 mL of distilled water was added to 200 μL of the supernatant, and the volume was made up to 2 mL. Then, 0.5 mL of 5% phenol and 2.5 mL of 98% H_2SO_4 were added to each sample. After vortexing, the samples were incubated at room temperature for 20 min, and the absorbance was measured at 490 nm using a UV spectrophotometer (Thermo Scientific™ Multiskan™ GO Microplate Spectrophotometer, Finland). To prepare total carbohydrate standards, a glucose stock solution was prepared by dissolving 20 mg in 20 mL of distilled water. Serial glucose concentrations were prepared from this stock solution, and absorbance was measured at the same wavelength (490 nm). The total carbohydrate content was expressed as mg glucose per gram of sample.

Total sugars were measured using the anthrone reagent method described by Nath et al. (Nath et al., 2015). Fresh tissues (200 mg) were homogenized in 1.5 mL of 80% ethanol and centrifuged at 14,000 rpm for 15 min at 4°C, and the supernatant was transferred to a fresh tube. Subsequently, 40 μL of the extract was mixed with 160 μL of distilled water in a glass test tube. Next, 2.5 mL of anthrone reagent was carefully added along the side of the glass tube. Then, the mixture was vortexed and incubated in a water bath at 90°C for 15 min, after which it was immediately cooled on ice. The absorbance of the samples was measured at 620 nm using a UV spectrophotometer (Thermo Scientific Multiskan GO Microplate Spectrophotometer, Finland), with the anthrone reagent serving as the blank. A standard curve of total carbohydrates was used to determine total sugar content, expressed as mg glucose per gram of sample.

Supplementary Text 9. Estimation of Total Flavonoids, Phenolics, and Phenylalanine Ammonia-Lyase (PAL) Activity

Fresh plant samples (100 mg) were dissolved in methanol and centrifuged at 14,000 rpm for 15 min. The clear supernatant was collected and stored in an amber tube for analysis.

Total flavonoid content was measured using a colorimetric method employing aluminum chloride (Pourmorad et al., 2006; Ribarova et al., 2005). Briefly, 100 μL of the fresh supernatant was mixed with 500 μL of methanol, 50 μL of 10% aluminum chloride, and 50 μL of 1 M NaOH. This mixture was then diluted by adding 300 μL of distilled water

and mixed thoroughly. After incubation at room temperature for 30 min, the absorbance of the samples was measured at 510 nm against a blank using a UV spectrophotometer (Thermo Scientific Multiskan GO Microplate Spectrophotometer, Finland). Quercetin was used to generate a standard calibration curve, with a stock solution prepared by dissolving 2 mg of quercetin in 1 mL of methanol. Standard solutions (5 to 80 μg/mL) were prepared through serial dilutions in methanol. The total flavonoid content of the extract was expressed as mg quercetin equivalents per gram of sample.

The total phenolic content was determined using the Folin–Ciocalteu reagent-based protocol described by Singleton and Rossi (Singleton & Rossi, 1965). Briefly, 50 μL of the methanol-extracted supernatant was mixed with 1 mL of a saturated sodium carbonate solution (1 mL, 2% w/v in water) and 1 N Folin–Ciocalteu reagent. The mixture was incubated in the dark for 30 min to initiate the reaction and then centrifuged. The absorbance of the resulting, blue-colored supernatant was then measured at 760 nm. A gallic acid-based standard curve was prepared by diluting gallic acid in methanol (5–40 μg/mL), and absorbance readings were taken against the reagent blank. Total phenolic content was expressed as mg gallic acid equivalents per gram of sample.

PAL activity was assessed following the protocol outlined by Fan et al. (Fan et al., 2014). Briefly, fresh maize leaves, stems, and roots (1 g) were blended in Tris-HCl buffer (6.5 mL, 50 mM, pH 8.8) containing β-mercaptoethanol (15 mM). After centrifugation of the extract at 4,000 rpm for 1 h, supernatant was collected for the enzymatic assay. For the assay, 1 mL of the extraction buffer was mixed with 0.4 mL of distilled water, 0.5 mL of 10 mM L-phenylalanine, and 0.1 mL of the supernatant, and incubated for 1 h at 37°C. The reaction was terminated by adding 0.5 mL of ethyl acetate, followed by centrifugation. The precipitate was dissolved in NaOH (3 mL, 0.05 M), and the absorbance was measured at 290 nm. Cinnamic acid was used as the standard, prepared by dissolving 2 mg in 1,000 mL of distilled water. From this stock solution, five different concentrations of cinnamic acid (0.2, 0.4, 0.6, 0.8, and 1.0 μL) were prepared for the standard curve. PAL activity was expressed as units per gram of protein.

Supplementary Text 10. Tandem Mass Spectrometry with Liquid Chromatography and Electrospray Ionization using a Quadrupole Time-of-Flight mass spectrometer (MS/MS-LC-ESI-Q-TOF analysis)

The extraction process involved adding 1 mL of methanol to the sample, followed by 10 min of sonication to ensure thorough mixing and dissolution of the compounds. The mixture was then centrifuged at 13,000 rpm for 5 min to separate the supernatant from any solid residues. The supernatant was subsequently filtered through a 0.45 μm syringe filter to remove any remaining particulate matter. Finally, the filtered solution was analyzed quantitatively to assess the target compounds.

The LC-ESI-Q-TOF analysis was performed under specific conditions. The LC method utilized a flow rate of 0.2 mL/min in gradient mode, with mobile phases consisting of distilled water containing 0.1% formic acid and acetonitrile (ACN) containing 0.1% formic acid. The column temperature was maintained at 40°C, and the analysis was conducted over 60 min using a C18 column.

For the MS/MS analysis, the mode was positive ionization, with an m/z scan range from 50 to 1,200 Da. The ion source gas pressure was set between 1.2 and 50 psi, with the curtain gas maintained at 30 psi. Additional parameters included a Collisionally Activated Dissociation (CAD) setting of 7, a temperature of 450°C, a declustering potential (DP) of 35 V, and a collision energy (CE) of 15 V. These optimized conditions facilitated the accurate detection and analysis of various compounds (Guijarro-Díez, García et al. 2013).

Supplementary Text 11. Quantification of SNO content

SNO concentrations were measured using a Sievers NOA-280i Nitric Oxide Analyzer (Estero, FL, USA), following the method described by **Hussain** et al. (2018). Firstly, fresh samples (200 mg) of maize leaves, stem, and roots were powdered in liquid nitrogen and then homogenized with 1 mL of 1× PBS buffer (pH 7.4). The homogenized solutions were centrifuged at 14,000 rpm and 4°C for 10 min to collect the fresh supernatant. Following the manufacturer's instructions, the protein content of each

sample was measured using the Bradford assay with a Coomassie (Bradford) protein assay kit (Thermo Fisher Scientific, Waltham, MA, USA). Briefly, Coomassie dye reagent (1.5 mL) was added to 30 μL of fresh supernatant in a 96-well plate and mixed thoroughly by pipetting. The absorbance of the sample was measured at 595 nm using a UV spectrophotometer (Thermo Scientific Multiskan GO Microplate Spectrophotometer, Finland). Subsequently, the freshly extracted supernatant (100 μL) was introduced into the reaction vessel of the analyzer, which contained a CuCl/cysteine reducing agent, and the peak values were documented. A GSNO-mediated standard curve was used to determine the SNO content, which was expressed as nanomoles per microgram of protein.

Supplementary Text 12. Quantification of phytohormones (Abscisic Acid, Jasmonic Acid, and Salicylic Acid)

Abscisic Acid (ABA)

The quantification of endogenous ABA in maize plants was performed following the method outlined by Khan et al. (Khan et al., 2018). Briefly, freeze-dried shoot and root samples (100 mg) were individually mixed with an extraction solution consisting of isopropanol and glacial acetic acid. The mixture was allowed to stand for 30 min, after which 100 ng of the internal standard [(±)-3,5,5,7,7,7-d6] ABA was added. The mixture was then filtered and concentrated under reduced pressure. Then, the obtained residue was mixed with 1 N NaOH, and the pH was adjusted to 12–13. Subsequently, pigments were removed using dichloromethane (CH_2Cl_2), and the pH was again adjusted to 2.5–3.5. Then, the mixture was concentrated using ethyl acetate under reduced pressure. The resulting solution was dissolved in phosphate buffer (pH 8.0), mixed with polyvinylpolypyrrolidone (PVPP, 1 g), and then filtered. The pH was adjusted to 2.5–3.5. The supernatant was collected, concentrated, and dried using nitrogen gas (N_2). The samples were subsequently methylated using diazomethane, dissolved in CH_2Cl_2 (50 μL), and analyzed via gas chromatography-mass spectrometry (GC/MS) using a 6890N network GC system and a 5973-network mass selective detector from Agilent Technologies after injecting 1 μL of each sample.

Jasmonic Acid (JA)

The JA content was quantified using GC/MS with a doubly labeled internal standard, [9,10-2H_2]-9,10-dihydro-JA, as outlined by Baldwin et al. (McCloud & Baldwin, 1997). To extract JA, 0.1 g of freeze-dried maize sample was mixed with an extraction solution containing acetone and 50 mM citric acid and stirred for 30 min. Subsequently, the mixture was filtered using a Buchner apparatus. The resulting extract was concentrated under reduced pressure, dissolved in phosphate buffer (100 mM, pH 7.5), and then acidified to a pH of 2.5. Diethyl aminoethyl cellulose (DEAE cellulose) was added to the samples and shaken for 1 h to facilitate the removal of impurities. Subsequently, chloroform was used to separate the organic layer, which was transferred to an open column containing anhydrous sodium sulfate ($NaSO_4$) to remove any residual water. The solution was again concentrated under reduced pressure to obtain the final product, which was reconstituted in ethyl ether and subsequently transferred to an amino cartridge (Grace Pure SPE Amino, Grace Dev, IL, USA). Contaminants were purged using chloroform and iso-propanol solution, followed by a wash with a blend of ethyl ether and acetic acid. Finally, the solution was poured, dispersed, and concentrated to the desired volume under reduced pressure. The final preparation was dissolved in ethyl ether, transferred to a vial, and dehydrated using nitrogen gas. The samples were then methylated using diazomethane, dissolved in anhydrous CH_2Cl_2, and analyzed using GC/MS.

Salicylic Acid (SA)

The SA content was assessed following the procedure outlined by Seskar et al. (Seskar et al., 1998) with slight modifications. The freeze-dried maize sample (0.1 g) was extracted using 90% methanol, with the extraction process facilitated by sonication and subsequent centrifugation at 12,000 rpm for 15 min at 4°C. The fresh supernatant was then mixed with 100% MeOH and concentrated using a SpeedVac (SPD2030, Thermo Fisher Scientific, Waltham, MA, USA). The resulting residue was mixed with 5% TCA and centrifuged. The supernatant obtained was mixed with a reaction mixture containing ethyl acetate, cyclopentane, and isopropanol. This solution was particularly enriched with the SA component and was subsequently dehydrated using nitrogen gas. The

resulting precipitates were redissolved in 100% MeOH (1 mL), and 20 μL of this solution was injected into the HPLC system for final quantitative estimation.
