# Supplementary material for: GSNO-functionalized hydroxyapatite nanofertilizer for prolonged delivery, detoxification, and yield improvement in maize
Source: Nat Commun. 2026 Sep 16;17:10896. doi: 10.1038/s41467-026-77610-0 (PMC13677726; doi:10.1038/s41467-026-77610-0)
Supplement: Supplementary file 2 [file 41467_2026_77610_MOESM2_ESM.pdf]

nature portfolio

Corresponding author(s): Byung-Wook Yun

Last updated by author(s): 09-April-2026

Reporting Summary

Nature Portfolio wishes to improve the reproducibility of the work that we publish. This form provides structure for consistency and transparency in reporting. For further information on Nature Portfolio policies, see our Editorial Policies and the Editorial Policy Checklist.

Statistics

For all statistical analyses, confirm that the following items are present in the figure legend, table legend, main text, or Methods section.

n/a	Confirmed	
☐	☒	The exact sample size (*n*) for each experimental group/condition, given as a discrete number and unit of measurement
☐	☒	A statement on whether measurements were taken from distinct samples or whether the same sample was measured repeatedly
☐	☒	The statistical test(s) used AND whether they are one- or two-sided<br>*Only common tests should be described solely by name; describe more complex techniques in the Methods section.*
☒	☐	A description of all covariates tested
☒	☐	A description of any assumptions or corrections, such as tests of normality and adjustment for multiple comparisons
☐	☒	A full description of the statistical parameters including central tendency (e.g. means) or other basic estimates (e.g. regression coefficient) AND variation (e.g. standard deviation) or associated estimates of uncertainty (e.g. confidence intervals)
☐	☒	For null hypothesis testing, the test statistic (e.g. *F*, *t*, *r*) with confidence intervals, effect sizes, degrees of freedom and *P* value noted<br>*Give P values as exact values whenever suitable.*
☒	☐	For Bayesian analysis, information on the choice of priors and Markov chain Monte Carlo settings
☒	☐	For hierarchical and complex designs, identification of the appropriate level for tests and full reporting of outcomes
☐	☒	Estimates of effect sizes (e.g. Cohen's *d*, Pearson's *r*), indicating how they were calculated

Our web collection on statistics for biologists contains articles on many of the points above.

Software and code

Policy information about availability of computer code

Data collection	No software was used
Data analysis	Analysis of variance (ANOVA), Post hoc comparisons using Tukey's test at p < 0.05 was conducted using Statistix 10. Graphs were generated using GraphPad Prism 9 Circular heatmap was generated using SRplot (http://www.bioinformatics.com.cn/srplot). Pearson's correlation matrix was visualized using the metal package in R Studio (R 4.4.3). Some of the content in Figure 11 is from BioRender for which license was obtained.

For manuscripts utilizing custom algorithms or software that are central to the research but not yet described in published literature, software must be made available to editors and reviewers. We strongly encourage code deposition in a community repository (e.g. GitHub). See the Nature Portfolio guidelines for submitting code & software for further information.

Data

Policy information about availability of data

All manuscripts must include a data availability statement. This statement should provide the following information, where applicable:

- Accession codes, unique identifiers, or web links for publicly available datasets
- A description of any restrictions on data availability
- For clinical datasets or third party data, please ensure that the statement adheres to our policy

All relevant data is available within the manuscript. Any additional data can be provided upon request to the corresponding authors.

Research involving human participants, their data, or biological material

Policy information about studies with human participants or human data. See also policy information about sex, gender (identity/presentation), and sexual orientation and race, ethnicity and racism.

Reporting on sex and gender	N/A
Reporting on race, ethnicity, or other socially relevant groupings	N/A
Population characteristics	N/A
Recruitment	N/A
Ethics oversight	Kyungpook National University, South Korea

Note that full information on the approval of the study protocol must also be provided in the manuscript.

Field-specific reporting

Please select the one below that is the best fit for your research. If you are not sure, read the appropriate sections before making your selection.

☒ Life sciences ☐ Behavioural & social sciences ☐ Ecological, evolutionary & environmental sciences

For a reference copy of the document with all sections, see nature.com/documents/nr-reporting-summary-flat.pdf

Life sciences study design

All studies must disclose on these points even when the disclosure is negative.

Sample size	Experiments were performed with multiple replicates each time. Some of the measurements needed destructive sampling. Repetitions with multiple replicates were considered as one replication (n = 1). The minimum number of replicates used for data analysis and making the graphs was three (n = 3).
Data exclusions	No data excluded
Replication	Experiments were performed at least three times independently. Experiments were performed in controlled environment to ensure same experimental/growth conditions. All attempts at reproducibility were successful.
Randomization	Experiments involving plants had at least 12 plants in each group. One group was considered a single replicate.
Blinding	Most of the measurements were performed by machines e.g. PCRs, Spectrophotometers, SEM/TEM etc. So blinding was not necessary. The measurements were entirely objective and automated.

Reporting for specific materials, systems and methods

We require information from authors about some types of materials, experimental systems and methods used in many studies. Here, indicate whether each material, system or method listed is relevant to your study. If you are not sure if a list item applies to your research, read the appropriate section before selecting a response.

Materials & experimental systems

n/a	Involved in the study	
☒	☐	Antibodies
☒	☐	Eukaryotic cell lines
☒	☐	Palaeontology and archaeology
☒	☐	Animals and other organisms
☒	☐	Clinical data
☒	☐	Dual use research of concern
☐	☒	Plants

Methods

n/a	Involved in the study	
☒	☐	ChIP-seq
☒	☐	Flow cytometry
☒	☐	MRI-based neuroimaging

Dual use research of concern

Policy information about dual use research of concern

Hazards

Could the accidental, deliberate or reckless misuse of agents or technologies generated in the work, or the application of information presented in the manuscript, pose a threat to:

No	Yes	
☐	☒	Public health
☒	☐	National security
☐	☒	Crops and/or livestock
☐	☒	Ecosystems
☒	☐	Any other significant area

Hazards: Here we developed a nanotechnology based fertilizer for improving crop yield. However, the RECKLESS misuse of this technology (or any other technology) may post threat to public health, crops and/or livestock. The effects of the RECKELSS misuse may be acute (quick or short term) or may appear after a long time. We have not shown any such studies in the manuscript.

For examples of agents subject to oversight, see the United States Government Policy for Institutional Oversight of Life Sciences Dual Use Research of Concern.

Experiments of concern

Does the work involve any of these experiments of concern:

No	Yes	
☒	☐	Demonstrate how to render a vaccine ineffective
☒	☐	Confer resistance to therapeutically useful antibiotics or antiviral agents
☒	☐	Enhance the virulence of a pathogen or render a nonpathogen virulent
☒	☐	Increase transmissibility of a pathogen
☒	☐	Alter the host range of a pathogen
☒	☐	Enable evasion of diagnostic/detection modalities
☒	☐	Enable the weaponization of a biological agent or toxin
☒	☐	Any other potentially harmful combination of experiments and agents

Precautions and benefits

Biosecurity precautions	Our study did not involve any human or plant pathogens or GMOs. However, all the experiments were performed in BSL2 laboratories.
Biosecurity oversight	N/A
Benefits	Improve plant health and increase the overall yield of crops. Reduce the cost of production. Improve soil health, Long term positive effects.
Communication benefits	The technology will have long-lasting positive effects on crop production. No risks were found with the use of the technology using the prescribed methods.

Plants

Seed stocks	The commercially available hybrid Maize variety, 'Jeil Alrokichal', was obtained from Jeilseed Company in South Korea.
Novel plant genotypes	N/A
Authentication	N/A
