# Supplementary material for: GSNO-functionalized hydroxyapatite nanofertilizer for prolonged delivery, detoxification, and yield improvement in maize
Source: Nat Commun. 2026 Sep 16;17:10896. doi: 10.1038/s41467-026-77610-0 (PMC13677726; doi:10.1038/s41467-026-77610-0)
Supplement: Supplementary file 3 [file 41467_2026_77610_MOESM3_ESM.pdf]

nature portfolio

Peer Review File

GSNO-Functionalized Hydroxyapatite Nanofertilizer for Prolonged Delivery Detoxification and Yield Improvement in Maize

Corresponding Author: Dr Byung-Wook Yun

This file contains all reviewer reports in order by version, followed by all author rebuttals in order by version.

Version 1:

Reviewer comments:

Reviewer #1

(Remarks to the Author)
This study developed a multifunctional GSNO-HAs-Fe-HAP nanocomposite, which enables sustained and smart-responsive co-release of essential nutrients. Under cadmium stress, this nanofertilizer effectively suppresses Cd translocation and oxidative stress by enhancing the accumulation of phytochelatins and S-nitrosothiols. However, to strengthen the mechanistic argumentation and address experimental limitations, moderate revisions are suggested:
The experiment utilized a high-concentration stress of 400 μM $CdCl_2$ for two weeks, which may overemphasize short-term detoxification effects and not accurately represent the chronic, low-concentration stress typical of agricultural environments.
Direct evidence is lacking to establish a causal link between S-nitrosylation modifications of ZmPCS or other transporter proteins by S-nitrosothiols (SNOs) and their enhanced functional activity.
There is no assessment of the long-term soil application of nanoparticles, including their fate, persistence, and potential ecological toxicity to non-target rhizosphere microbial communities.
To clarify whether nanoparticles regulate the redox system primarily at the transcriptional level or via post-translational modifications, Western blot analysis is suggested to confirm protein abundance of key antioxidant enzymes (e.g., POD, APX), coupled with enzyme activity kinetics studies to evaluate the direct effects of exogenous GSNO on purified enzymes.
To verify long-term agronomic benefits and environmental safety for commercialization, a long-term pot experiment is recommended: using low Cd concentrations (e.g., 20–50 μM) until the V6 stage or maturity harvest of maize to assess Cd accumulation in grains, supplemented with microbiome analysis.

Reviewer #2

(Remarks to the Author)
The manuscript titled “Beyond Traditional Fertilizers: GSNO-Functionalized Hydroxyapatite Nanofertilizer for Prolonged NO Delivery, Metal Detoxification, and Crop Resilience” presents an approach to immobilize S-nitrosoglutathione (GSNO) onto hydroxyapatite (HAP) ions and humic acid nanoparticles (NPs). The nanoparticles demonstrated prolonged nitric oxide (NO) release (over 6 days), which was enhanced under acidic and cadmium (Cd) stress conditions. Characterization of the resulting nanocomposite was conducted, and the findings are highly valuable and of significant practical relevance. However, from a physiological perspective, several issues should be more precisely defined before the manuscript is considered for publication in Nature Communications.

Title – The term crop resilience is not appropriate in this context. The study only examined the effect of GSNO-HAs-Fe-HAP nanoparticles on selected elements of tolerance mechanisms.

Missing Control – The manuscript lacks a crucial control. The authors analyze various parameters and attribute the observed effects to NO released from the GSNO nanocomposite. However, there is no experimental variant in which NO is directly scavenged to confirm that the effects are indeed mediated by NO. The effects of the HAs-Fe-HAP composite should be included, or at the very least, an NO scavenger should be used.

Cadmium Stress Intensity – The authors used a specific concentration of Cd (400 μM $CdCl_2$), but the stress intensity it represents is unclear. A tolerance index should be provided.

NO Release Kinetics (Fig. 2G) – The figure compares the rate and kinetics of NO release from GSNO using the same measurement method. Does the figure show NO generation immediately upon solution preparation? The experiment indicates that the maximum NO level/concentration occurs at 0 min, with no NO detected after 30 min. However, NO is typically released from GSNO gradually, and the release depends on external factors such as light. It has been documented that the half-life of GSNO in solution is approximately 7 hours. Under what conditions was this experiment conducted?

Photosynthetic Efficiency – Chlorophyll content alone does not indicate photosynthetic efficiency. It would be beneficial to determine Fv/Fm, a key indicator of photosynthetic performance and plant stress status, reflecting the maximum quantum efficiency of photosystem II (PSII).

Redox Homeostasis – The authors suggest that GSNO-HAs-Fe-HAP regulates redox homeostasis in maize. However, they did not analyze crucial parameters related to redox balance under cadmium stress and NO release from GSNO. Specifically, the GSH and GSH/GSSG ratio are missing.

Cadmium Toxicity Reference – Nitric oxide has also been shown to contribute to cadmium toxicity in Arabidopsis by promoting cadmium accumulation in roots and regulating genes involved in iron uptake. The authors should reference this work.

Version 2:

Reviewer comments:

Reviewer #1

(Remarks to the Author)
The authors have adequately responded to the reviewers' concerns and supplemented select experiments, thereby improving the paper's readability, innovation, and theoretical framework. Consequently, the manuscript is considered suitable for acceptance in Nature Communications .

Reviewer #2

(Remarks to the Author)
Although the revised version of the manuscript has been improved and enriched compared with the previous submission, the study still contains several conceptual and methodological weaknesses that, in my opinion, prevent it from being ready for publication in its current form.

1. Title – Although “crop improvement” better reflects the presented results than the previous version, the title still requires substantial revision. At present, it remains overly broad and overgeneralized. Since all experiments were conducted exclusively on a single monocotyledonous species, maize (Zea mays), it is premature to broadly refer to “crop improvement” or imply general applicability across crop species. Responses to Cd stress, NO signaling, and nanoparticle treatments are strongly species- and genotype-dependent, and the presented data support conclusions only for the investigated species. Importantly, mechanisms underlying yield formation and stress adaptation vary substantially among crops and cannot be generalized from a single-species study. While the Discussion may address the potential applicability of this approach to other species, such implications currently remain hypothetical and experimentally unverified.

Importantly, the manuscript still does not provide sufficient information regarding the maize genotype used in the study. In the Materials and Methods section, the authors refer only to “maize seeds,” without specifying the cultivar, hybrid, inbred line, or seed source. Consequently, the study operates solely at the species level without identifying the genetic background, which substantially limits reproducibility and weakens interpretation of the results in the context of genotypic variability. This is particularly relevant because responses to Cd stress and NO-related signaling are well known to be strongly genotype dependent.

For these reasons, I strongly recommend revising the title to explicitly indicate maize as the experimental species and to avoid overgeneralization beyond the scope of the presented dataset.

2. Cadmium Stress Intensity – There is a clear discrepancy between the authors’ description of the treatment as “acute, high-concentration cadmium stress (400 µM for two weeks)” and the actual phenotypic outcomes presented in the manuscript, particularly in Supplementary Figure S17. In plant stress physiology, acute stress generally implies rapid and severe toxicity resulting in strong growth inhibition and often irreversible damage. However, the plants described in this study survive the treatment, resume growth, complete their life cycle, and maintain photosynthetic activity over an extended period. These observations are more consistent with a transient and recoverable stress rather than a truly acute one. Therefore, the use of the term “acute” appears to overstate the severity of the stress conditions and consequently amplifies the perceived effectiveness of the nanoparticle treatment.

Importantly, the manuscript emphasizes stress mitigation effects without first quantitatively establishing the actual severity of stress experienced by the plants. Integrative physiological parameters, such as the tolerance index, should be presented earlier in the Results section to clearly demonstrate stress intensity before discussing the protective effects of the

nanoparticles.

In addition, at which developmental stage was the tolerance index determined? This should be explicitly clarified.

Furthermore, the Materials and Methods section describing the long-term experiment still lacks essential details regarding nanoparticle application. It remains unclear whether the nanoparticles were applied only once at the beginning of the experiment or repeatedly during plant growth.

3. NO Release Kinetics (Fig. 2G) – Essential methodological details should be included directly in the manuscript rather than referring readers to previous publications. At present, the experimental conditions used for measuring NO release from GSNO remain insufficiently described. Specifically, it is unclear:
• how long after GSNO addition to the reducing buffer NO detection was initiated,
• what is meant by the term “reducing buffer,”
• what the redox characteristics or redox potential of this buffer were,
• and whether the measurements were conducted under light or dark conditions.

Version 3:

Reviewer comments:

Reviewer #2

(Remarks to the Author)
The revised manuscript has been substantially improved, and I appreciate the considerable effort the authors have made to address the majority of the concerns raised during the previous review round. In particular, the manuscript now provides important methodological details regarding the maize genotype, experimental design, tolerance index assessment, and NO-release measurements.

1. I have one remaining comment concerning the NO-release experiment presented in Figure 2G. As a researcher who routinely works with various nitric oxide detection and quantification techniques, I found the additional methodological information helpful. However, the mechanistic interpretation of the NO-release kinetics remains unclear. The authors state that the measurements were performed in a sealed chamber, under dark conditions, and without the use of a strong reducing buffer, using 1 M citric acid as the reaction medium. Under these conditions, it is not immediately evident which physicochemical processes are responsible for the temporally variable NO-release profile observed over several days daily fluctuations in case of GSNO-HAs-Fe-HAP). While the manuscript proposes a potential role for humic acids and Fe-mediated interactions in GSNO transformation and NO release, this explanation remains largely speculative. Therefore, I encourage the authors to expand the discussion of Figure 2G and provide a more detailed explanation of the mechanisms that may underlie the observed NO-release dynamics. Such clarification would strengthen one of the central findings of the study and improve the interpretation of the NO-release data.

2. The presentation of the Cd tolerance index in Figure 4A,B is somewhat confusing. Since the tolerance index is intended to assess plant performance under cadmium stress relative to unstressed controls, it appears that all treatments included in these panels were exposed to Cd. If this is the case, the labels “Control”, “NPK”, and “GSNO-HAs-Fe-HAP” may be misleading and should be revised to indicate Cd-treated groups as in other parameters.

Open Access This Peer Review File is licensed under a Creative Commons Attribution 4.0 International License, which permits use, sharing, adaptation, distribution and reproduction in any medium or format, as long as you give appropriate credit to the original author(s) and the source, provide a link to the Creative Commons license, and indicate if changes were made.
In cases where reviewers are anonymous, credit should be given to 'Anonymous Referee' and the source.
The images or other third party material in this Peer Review File are included in the article's Creative Commons license, unless indicated otherwise in a credit line to the material. If material is not included in the article's Creative Commons license and your intended use is not permitted by statutory regulation or exceeds the permitted use, you will need to obtain permission directly from the copyright holder.
To view a copy of this license, visit https://creativecommons.org/licenses/by/4.0/

RESPONSE TO REVIEW COMMENTS

We thank the editor(s) and the reviewers for their time, effort, and insightful critique on our manuscript. Their comments have certainly improved the manuscript. We have now performed fresh experiments, generating extensive new data. The new data have been incorporated into the manuscript, and the comments of the reviewers have been addressed. All the changes in the manuscript have been highlighted in green. Below, we provide a point-to-point response to the review comments. We hope that the editors/reviewers will find these changes satisfactory.

Reviewer #1 (Remarks to the Author):

This study developed a multifunctional GSNO-HAs-Fe-HAP nanocomposite, which enables sustained and smart-responsive co-release of essential nutrients. Under cadmium stress, this nanofertilizer effectively suppresses Cd translocation and oxidative stress by enhancing the accumulation of phytochelatins and S-nitrosothiols. However, to strengthen the mechanistic argumentation and address experimental limitations, moderate revisions are suggested:

1. The experiment utilized a high-concentration stress of 400 μM $CdCl_2$ for two weeks, which may overemphasize short-term detoxification effects and not accurately represent the chronic, low-concentration stress typical of agricultural environments.

Response: We thank the reviewer for this insightful observation. We agree that a 400 μM $CdCl_2$ treatment for two weeks represents an acute, high-stress condition, and it may not mirror the chronic, low-concentration cadmium exposure. The primary goal of this study was to conduct a proof-of-concept evaluation of the cadmium-mitigating potential of GSNO-HAs-Fe-HAP NPs under conditions that would produce rapid, unambiguous effects. High-concentration, short-duration stress protocols are widely used in early-stage nano-remediation research to screen for efficacy and identify underlying mechanistic pathways. Such studies also help to establish a clear "worst-case scenario" benchmark before proceeding to test other variations in the treatments. In our case, the 400 μM level was chosen because preliminary experiments (and

published literature) confirmed that lower concentrations (e.g., 50–100 µM) produced only marginal growth reductions in maize within a two-week window, making it difficult to statistically distinguish treatment effects. Literature shows that scientists have used up to 400 µM and 800 µM Cd (https://doi.org/10.1038/s41598-025-94385-4). Furthermore, the above high concentrations are applied via hydroponic systems, where it is generally more bioavailable and hence more robust/toxic compared to soil treatment (as in this study), which is a relatively more variable medium. Our acute stress model emphasizes short-term detoxification mechanisms. The nanoparticles designed in our lab mitigate the stress induced by a significantly higher Cd concentration (400 µM). To reflect on the chronic, long-term field conditions, we have measured several long-term parameters related to plant yield and impact on soil properties and soil microbiota that clearly support our findings. Nevertheless, we understand the reviewer's concern. We have now added the following statement to the Conclusion section of the revised manuscript.

"*While the present study demonstrates the efficacy of GSNO-HAs-Fe-HAP NPs under acute, high-concentration cadmium stress (400 µM for two weeks), other, low-dose treatments my be tested to represent the chronic, low-concentration exposure. Future studies should therefore evaluate the performance of these nanoparticles under variable Cd concentrations or Cd-contaminated soils*".

2. Direct evidence is lacking to establish a causal link between S-nitrosylation modifications of ZmPCS or other transporter proteins by S-nitrosothiols (SNOs) and their enhanced functional activity.

Response: Agreed. The current manuscript does not provide direct evidence of ZmPCS being S-nitrosylated. The NPs we designed release NO, which increased the global SNO content of plants. We have demonstrated that the higher SNOs (Figure 7) improved cadmium stress mitigation via quantitative measurements of several parameters, including expression of ZmPCS and several other genes. Fresh experimental evidence in the revised manuscript involving the NO scavenger CPTIO shows significantly lower SNO content (Supplementary Figure S9) with concomitant effects on several parameters, clearly supporting our hypothesis.

Furthermore, we have now performed advanced computational analysis of ZmPCS via GPS-SNO, which identified three cysteine residues within ZmPCS as potential S-nitrosylation targets with significantly higher confidence (see results section 3.8 of the revised manuscript. Further analysis of the protein to find free solvent-accessible cysteines indicated that all three of the identified cysteine molecules showed high free SASA values and side-chain SASA values, further supporting the availability of these cysteines to SNO donors. Taken together, these computational predictions suggest that ZmPCS is structurally capable of being S-nitrosylated at specific cysteine residues.

3. There is no assessment of the long-term soil application of nanoparticles, including their fate, persistence, and potential ecological toxicity to non-target rhizosphere microbial communities.

Response: We have now performed fresh experiments, including metagenomic analysis, across six different experimental groups to determine the impact of the NPs on the rhizosphere bacterial and fungal microbiome, specifically under Cd stress. The analysis involves alpha and beta diversity analysis, relative abundance of the bacterial and fungal microbes at the phylum, genus, and species levels. Shanon index, Jaccard, Bray-Curtis, Unweighted UniFrac, and Weighted UniFrac principal component analyses have also been performed (Figure 10 and Supplementary Figures S13 to S15). Furthermore, soil attributes such as, EC, CEC, soil N, P, K contents, pH, Ca, Mg, and organic matter (OM) have also been measured and shown in Figure 10 and Supplementary Figure S13.

4. To clarify whether nanoparticles regulate the redox system primarily at the transcriptional level or via post-translational modifications, Western blot analysis is suggested to confirm protein abundance of key antioxidant enzymes (e.g., POD, APX), coupled with enzyme activity kinetics studies to evaluate the direct effects of exogenous GSNO on purified enzymes.

Response: We thank the reviewer for this excellent and mechanistically important suggestion. We agree that distinguishing between transcriptional and translational/post-translational regulation of antioxidant enzymes is critical for understanding how GSNO-

HAs-Fe-HAP NPs exert their protective effects. We regret that we cannot perform the suggested Western blot and purified enzyme kinetics at this stage due to time constraints and lack of funds. However, our existing data provide indirect clues:

Measurement	What It Shows	Relevance to Reviewer's Question
Enzyme activity (POD, APX, SOD, CAT)	Functional output is increased	Both mechanisms could explain this.
SNO content (NOA analysis) + GPS-SNO analysis + Solvent accessibility analysis.	SNO levels are elevated under NP treatment.<br>Three cysteine molecules are solvent-exposed and could be the targets of S-Nitrosylation with high confidence	Supports potential for post-translational S-nitrosylation.
GSH/GSSG ratio.	Redox state is altered.	Post-translational regulation (e.g., via redox-sensitive cysteines) is plausible
Experiments involving cPTIO (NO scavenger)	The NO released by the NPs plays an essential role in their effects on plants, soil, microbiome etc.	Suggests post-translational S-nitrosylation may be involved

Our data demonstrate that GSNO-HAs-Fe-HAP NPs enhance the activity of antioxidant enzymes (POD, APX, SOD, CAT) under cadmium stress. Increased enzyme activity could arise from transcriptional upregulation (increased protein abundance), post-translational modification (e.g., direct S-nitrosylation of active-site cysteines), or both. Our observation that NP treatment elevates endogenous SNO levels, combined with the fact that the NO scavenger cPTIO abolishes the protective effect, is consistent with a post-translational mechanism involving S-nitrosylation. However, we cannot rule out transcriptional contributions. Definitive resolution via Biotin-Switch + Western blot alongside activity kinetics on purified enzymes exposed to exogenous GSNO—experiments are indeed critical directions for future mechanistic follow-up studies."

5. To verify long-term agronomic benefits and environmental safety for commercialization, a long-term pot experiment is recommended: using low Cd concentrations (e.g., 20–50 μM) until the V6 stage or maturity harvest of maize to assess Cd accumulation in grains, supplemented with microbiome analysis.

Response: To determine the long-term agronomic benefits and environmental safety concerns, we have performed additional experiments generating fresh data. These involve quantitative measurements of yield-related parameters such as cob length, rows/cob, grains/row, 100 grain weight (Figure 10), number of leaves, plant height, stem length/diameter (Figure S17). Furthermore, data on physiological parameters have also been collected from the start of the experiment up to physiological maturity/termination of experiments. These include the net photosynthetic rate, stomatal conductance, transpiration rate, and intercellular CO_2 concentration (Figure S17). In addition, several grain parameters have also been measured, including carbohydrates, proteins, phosphorus, and potassium content in the grains (Figure 10). Cadmium tolerance index of the shoots and roots has also been measured. These new data support and further strengthen our previous findings and significantly improved the manuscript.

Reviewer #2 (Remarks to the Author):

1. The manuscript titled "Beyond Traditional Fertilizers: GSNO-Functionalized Hydroxyapatite Nanofertilizer for Prolonged NO Delivery, Metal Detoxification, and Crop Resilience" presents an approach to immobilize S-nitrosoglutathione (GSNO) onto hydroxyapatite (HAP) ions and humic acid nanoparticles (NPs). The nanoparticles demonstrated prolonged nitric oxide (NO) release (over 6 days), which was enhanced under acidic and cadmium (Cd) stress conditions. Characterization of the resulting nanocomposite was conducted, and the findings are highly valuable and of significant practical relevance. However, from a physiological perspective, several issues should be more precisely defined before the manuscript is considered for publication in Nature Communications.

2. Title – The term crop resilience is not appropriate in this context. The study only examined the effect of GSNO-HAs-Fe-HAP nanoparticles on selected elements of tolerance mechanisms.

   Response: Following the facile one-pot synthesis of GSNO-HAs-Fe-HAP-NPs, the nanoparticles were characterized (SEM/TEM, FTIR, EDX, and TGA, nutrient release profiling via ICP-OES, SEC, NO-release kinetics, and plant uptake of FITC-labelled NPs).

   Afterwards, the following measurements were done.

- **Physiological & Biochemical Assays**: Photosynthetic efficiency, ROS detection (NBT/DAB staining), antioxidant enzyme activity (APX, SOD, POD, CAT), and osmolyte (proline, sugars) quantification.
- **Molecular Analyses**, including qRT-PCR analysis to determine changes in the expression of metal transporters, antioxidants, and phenylpropanoid pathway genes. MS/MS-LC-ESI-Q-TOF and GC-MS for metabolite profiling (phenols, flavonoids, ABA, JA, SA) and plant Cd tolerance index.
- **Soil Health Metrics**: Analysis of organic matter, CEC, and nitrogen forms (NO_3^-, NH_4^+), P, K, and the impact of NPs on bacterial and fungal rhizosphere microbiome via metagenomics analysis.

- **Yield-related parameters** to determine the long-term impact of NPs, such as cob length, rows/cob, grains/row, 100 grain weight (Figure 10), number of leaves, plant height, and stem length/diameter (Figure S17). Furthermore, data on physiological parameters have also been collected from the start of the experiment up to physiological maturity/termination of experiments to get an idea about the long-term impact of the NPs. These include the net photosynthetic rate, stomatal conductance, transpiration rate, and intercellular CO_2 concentration (Figure S17). In addition, several grain parameters have also been measured, including carbohydrates, proteins, phosphorus, and potassium content in the grains (Figure 10).

Data on the broad spectrum of parameters described above indicated that the designed NPs not only enabled the maize plants to resist cadmium-induced stress but also significantly improved the overall crop health and soil attributes. This is why we used the word "resilience" in the title. However, we have now replaced this word with "improvement." Hence, the revised title is now **"Beyond Traditional Fertilizers: GSNO-Functionalized Hydroxyapatite Nanofertilizer for Prolonged NO Delivery, Metal Detoxification, and Crop Improvement".**

3. Missing Control – The manuscript lacks a crucial control. The authors analyze various parameters and attribute the observed effects to NO released from the GSNO nanocomposite. However, there is no experimental variant in which NO is directly scavenged to confirm that the effects are indeed mediated by NO. The effects of the HAs-Fe-HAP composite should be included, or at the very least, an NO scavenger should be used.

Response: To confirm that the effects are indeed mediated by NO, we have now performed additional experiments involving the NO scavenger cPTIO. These experiments have generated extensive fresh data spanning over five new supplementary figures. The data include measurement of phenotypic parameters, chlorophyll and carotenoid contents (Figure S3), ROS, including leaf/stem/root H_2O_2, MDA contents, antioxidant enzyme (*SOD, POD, APX, CAT, P5CR1, PCS, GSNOR1, ABCC1, ABCG40*) activities (Figure S4), gene expression (SOD, POD, APX, CAT)

presented as Figure S5, leaf/stem/root metabolite contents (proline, total proteins, carbohydrates, soluble sugars) presented as Figure S6, measurements of oxidized and reduced glutathione (Figure S9), and S-nitrosothiol levels (Figure S9). The fresh data corroborate and support the findings of the study, attributing the observed effects to NO released from the GSNO nanocomposite. Results from the fresh data have been described in the revised manuscript and highlighted in green where comparisons are drawn with the cPTIO control.

4. Cadmium Stress Intensity – The authors used a specific concentration of Cd (400 µM $CdCl_2$), but the stress intensity it represents is unclear. A tolerance index should be provided.

   Response: Tolerance index has been calculated. Please refer to sections 2.8 and 3.12 of the revised manuscript along with supplementary figure S17.

5. NO Release Kinetics (Fig. 2G) – The figure compares the rate and kinetics of NO release from GSNO using the same measurement method. Does the figure show NO generation immediately upon solution preparation? The experiment indicates that the maximum NO level/concentration occurs at 0 min, with no NO detected after 30 min. However, NO is typically released from GSNO gradually, and the release depends on external factors such as light. It has been documented that the half-life of GSNO in solution is approximately 7 hours. Under what conditions was this experiment conducted?

   Response: S-nitrosothiol (SNO) levels in the plant samples were quantified using a Sievers NOA-280i Nitric Oxide Analyzer (Section 2.6 of the manuscript). This follows the same protocol used for SNO measurements. Please refer to our previously published protocol (https://doi.org/10.1007/978-1-4939-7695-9_17). The release rate/kinetics of NO from GSNO depend on several conditions, i.e., concentration of the solution, the solvent used, pH, temperature, and light. Hence, the half-life of GSNO can range from short (a few seconds) to significantly longer durations (up to 49 days) depending upon conditions (Please refer to https://doi.org/10.1016/j.niox.2019.01.002 and https://doi.org/10.1016/j.niox.2019.08.002 for further details). Furthermore, the half-

life of the GSNO solution (and any other NO donor) is determined under base conditions without any reducing agents, where spontaneous NO release is recorded. In this study, we used the NO analyzer where the GSNO solution (or any other NO donor) is added to a reducing buffer. The reducing buffer chemically induces NO release via reductive cleavage of the S–NO bond. The near-instantaneous NO release from GSNO observed in the Sievers NOA assay reflects the chemical reduction of the S–NO bond under forced reducing conditions, not the spontaneous decomposition rate under physiological or environmental conditions. In the absence of exogenous reducing agents, GSNO has a reported half-life of approximately 49 days at 25°C in the dark. On the other hand, the GSNO-HAs-Fe-HAP NPs are designed to protect GSNO from premature NO release, thereby achieving prolonged NO delivery in the soil-plant system—a claim supported by a sustained NO release for over 6 days and the physiological effects observed over the entire growth period.

6. Photosynthetic Efficiency – Chlorophyll content alone does not indicate photosynthetic efficiency. It would be beneficial to determine Fv/Fm, a key indicator of photosynthetic performance and plant stress status, reflecting the maximum quantum efficiency of photosystem II (PSII).

   Response: Fresh experiments were performed, and data were collected to determine Fv/Fm. Please refer to section 3.4 and Figure S3F-H of the revised manuscript.

7. Redox Homeostasis – The authors suggest that GSNO-HAs-Fe-HAP regulates redox homeostasis in maize. However, they did not analyze crucial parameters related to redox balance under cadmium stress and NO release from GSNO. Specifically, the GSH and GSH/GSSG ratio are missing.

Response: Fresh experiments were performed, and data were collected to determine oxidized glutathione (GSSG), reduced glutathione (GSH) and GSH/GSSG ratio. Please refer to section 3.8 and Figure S8 of the revised manuscript. We recorded a significantly higher GSH/GSSG ratio in response to Cd+ NPs and NP application alone (Figure S8G-I). A high GSH:GSSG ratio indicates a reducing environment, which is essential for

maintaining protein thiols in their reduced (–SH) state, protection against oxidative damage and supporting enzyme function.

8. Cadmium Toxicity Reference – Nitric oxide has also been shown to contribute to cadmium toxicity in Arabidopsis by promoting cadmium accumulation in roots and regulating genes involved in iron uptake. The authors should reference this work.

Response: The study was published by Besson-Bard et al. It is indeed an interesting study. The paper has been cited in the discussion section, and a brief discussion paragraph has also been added. NO-biology in plants is indeed highly complex. The study by Besson-Bard et al (Plant Physiology) demonstrated that NO production exacerbates Cd toxicity in Arabidopsis by promoting Cd accumulation in the roots via up-regulation of Cd/Fe transporter *IRT1* and promoting Cd uptake and reducing Ca^{2+} uptake. The findings of this study are not contradictory but rather highlight a critical distinction with Bensson-Bard et al. While uncontrolled endogenous NO may facilitate metal uptake, the controlled, exogenous delivery of NO and subsequent SNOs via our GSNO-HAs-Fe-HAP nanocomposite bypasses this pathological pathway, instead enhancing antioxidant defense and directly chelating Cd^{2+}, thereby reversing the toxic cascade observed in the above study.

RESPONSE TO REVIEWER COMMENTS

We thank the editor(s) and the reviewers for their time, effort, and valuable critique on our manuscript. Their comments have certainly improved the manuscript. Reviewer 1 has already accepted the manuscript for publication. The second reviewer's comments are very insightful and encouraging. We have provided a point-to-point response to their comments below and further improved the manuscript. All the changes in the manuscript have been highlighted in green. We hope that the editors/reviewers will find these changes satisfactory.

Reviewer #1 (Remarks to the Author):

The authors have adequately responded to the reviewers' concerns and supplemented select experiments, thereby improving the paper's readability, innovation, and theoretical framework. Consequently, the manuscript is considered suitable for acceptance in Nature Communications.

Response: Thanks very much for the time and effort the reviewer has put into improving our manuscript.

Reviewer #2 (Remarks to the Author):

Although the revised version of the manuscript has been improved and enriched compared with the previous submission, the study still contains several conceptual and methodological weaknesses that, in my opinion, prevent it from being ready for publication in its current form.

1. Title – Although "crop improvement" better reflects the presented results than the previous version, the title still requires substantial revision. At present, it remains overly broad and overgeneralized. Since all experiments were conducted exclusively on a single monocotyledonous species, maize (Zea mays), it is premature to broadly refer to "crop improvement" or imply general applicability across crop species. Responses to Cd stress, NO signaling, and nanoparticle treatments are strongly species- and genotype-dependent, and the presented data support conclusions only for the investigated species. Importantly, mechanisms underlying yield formation and stress adaptation vary substantially among crops and cannot be generalized from a single-species study. While the Discussion may address the potential applicability of this approach to other species, such implications currently remain hypothetical and experimentally unverified.

Response: We completely agree with the reviewer's insightful comment. The study involves experiments conducted exclusively on maize (*Zea mays*). Furthermore, responses to Cd stress and NO signaling may vary among species/genotypes to some extent. Accordingly, we have revised the title to explicitly indicate maize (*Zea mays* L.) as the experimental species. The revised title of the manuscript is "**Beyond Traditional Fertilizers: GSNO-Functionalized Hydroxyapatite Nanofertilizer for Prolonged NO Delivery, Metal Detoxification, and Yield Improvement in Maize**".

2. Importantly, the manuscript still does not provide sufficient information regarding the maize genotype used in the study. In the Materials and Methods section, the authors refer only to "maize seeds," without specifying the cultivar, hybrid, inbred line, or seed source. Consequently, the study operates solely at the species level without identifying the genetic background, which substantially limits reproducibility and weakens interpretation of the results in the context of genotypic variability. This is particularly relevant because responses to Cd stress and NO-related signaling are well known to be strongly genotype-dependent. For these reasons, I strongly recommend revising the title to explicitly indicate maize as the experimental species and to avoid overgeneralization beyond the scope of the presented dataset.

Response: The relevant section 5.3 in Materials and Methods has now been updated. We have added information regarding the variety of the maize cultivar used in this study. Specifically, the following text has been added to section 5.3 of the materials and methods section in the revised manuscript "Maize seeds [variety Suwonchal 97 (a single-cross F1 hybrid developed by the Rural Development Administration - RDA, South Korea, currently awaiting official cultivar registration)]"….Furthermore, we do

have a cultivar summary sheet from the Maize Breeding Team describing the characteristics of Suwonchal 97. However, we have been informed that information regarding the parental lines (female and male parents) cannot be disclosed at this time because an official publication describing the cultivar is awaiting.

3. Cadmium Stress Intensity – There is a clear discrepancy between the authors' description of the treatment as "acute, high-concentration cadmium stress (400 µM for two weeks)" and the actual phenotypic outcomes presented in the manuscript, particularly in Supplementary Figure S17. In plant stress physiology, acute stress generally implies rapid and severe toxicity resulting in strong growth inhibition and often irreversible damage. However, the plants described in this study survive the treatment, resume growth, complete their life cycle, and maintain photosynthetic activity over an extended period. These observations are more consistent with a transient and recoverable stress rather than a truly acute one. Therefore, the use of the term "acute" appears to overstate the severity of the stress conditions and consequently amplifies the perceived effectiveness of the nanoparticle treatment.

Response: The term "Acute" has been removed from the revised manuscript.

4. Importantly, the manuscript emphasizes stress mitigation effects without first quantitatively establishing the actual severity of stress experienced by the plants. Integrative physiological parameters, such as the tolerance index, should be presented earlier in the Results section to clearly demonstrate stress intensity before discussing the protective effects of the nanoparticles.

   In addition, at which developmental stage was the tolerance index determined? This should be explicitly clarified.

   Furthermore, the Materials and Methods section describing the long-term experiment still lacks essential details regarding nanoparticle application. It remains unclear whether the nanoparticles were applied only once at the beginning of the experiment or repeatedly during plant growth.

Response: We appreciate the reviewer's constructive and insightful feedback. We further agree that establishing the severity of Cd stress earlier in the manuscript would provide a much stronger logical foundation for evaluating the nanoparticles' subsequent protective effects. In accordance with the reviewer's suggestion, we have restructured the Results section to present the cadmium tolerance index at the beginning (section 2.4). This establishes a quantitative baseline for the actual stress intensity prior to discussing the long-term mitigation effects.

Furthermore, we have clarified in the revised text (section 5.6) that the Cd tolerance index was determined **at the V5 vegetative stage (five fully developed leaves)**. Since this data was presented in the manuscript following the first round of review, it was placed in the new figures. Although it's a relatively early-stage stress assessment and

the data were collected at this earlier stage of growth, it was inadvertently described alongside the long-term data in Figure S17, which shows "long-term impact" (98-day physiological data). Therefore, as suggested by the reviewer, the Cd-tolerance index data (Figure S17A,B) are now presented earlier in the revised manuscript (Figure 4A,B). The associated text has also been shifted to section 2.4 of the results section. By distinguishing between the initial stress assessment (V5 stage) and the subsequent long-term physiological monitoring (up to 98 days), the revised manuscript now features a much clearer and chronological narrative.

Additional details regarding the long-term experiment have been added to the methodology section 5.3 of the revised manuscript, where we now explicitly detail that the nanoparticle and fertilizer treatments were applied twice during the entire growth period: an initial application at the time of transplantation and a second application at the tasseling stage.

5. NO Release Kinetics (Fig. 2G) – Essential methodological details should be included directly in the manuscript rather than referring readers to previous publications. At present, the experimental conditions used for measuring NO release from GSNO remain insufficiently described. Specifically, it is unclear:
   • how long after GSNO addition to the reducing buffer NO detection was initiated,
   • what is meant by the term "reducing buffer,"
   • what the redox characteristics or redox potential of this buffer were,
   • and whether the measurements were conducted under light or dark conditions.

Response: We are a Nitric Oxide Research group. A number of methods for detecting and measuring nitric oxide in biological samples have been described so far, each with distinct advantages and trade-offs. These methods are of FIVE common types: **fluorescence**, **electrochemical sensors**, **Griess assay, electron paramagnetic resonance (EPR),** and **chemiluminescence.**

Among all these methods, the chemiluminescence-based NO measurement using Siever's Nitric Oxide Analyzer (Siever's NOA) is one of the most sophisticated methods for NO measurements with a detection sensitivity of ~1 picomole (pM) for liquid samples. It's the method of choice for absolute quantification and the gold standard of accuracy. It is ideal for measuring gaseous NO and conducting real-time kinetic studies of NO release. It can also be used to analyze samples to distinguish among different NO-related molecules (such as nitrite, nitrate, and S-nitrosothiols).
The instrument can be used in "**Aqueous mode**" [(for liquid samples – where the sample is reduced by a strong copper or iodide-based reducing buffer to release all its NO quickly – as in the case of measuring the total S-nitrosothiols (NO attached endogenous proteins)] and the "**Gas mode**" (a strong reducing buffer is not involved, and liquid, dry, or live samples can be placed in the chamber to measure NO release over a longer duration). In such cases, depending upon the sample under study, the NO is either released spontaneously, via photoreduction or due to mild acidic environment.

Our study involved both the “Gas NO” measurements (Figure 2G) and S-Nitrosothiol measurements (Supplementary Figure S9).

Furthermore, we have designed customized chambers for the instrument so that it can be used to measure NO gas released from dry powder, and even live plants. For reference, I am providing two images below (only for the reviewer – not for the manuscript). Notice the four transparent chambers in the center of the 1st image or the falcon tube in the second image. We use these custom chambers to operate the machine in “Gas Mode”. Samples ranging from small to large can be kept in these chambers to measure the amount of NO released. We regularly used these methods for NO measurements, and that is why we referred to previously published literature. However, we have now added a detailed methodology for measuring NO release kinetics to the materials and methods section of the revised manuscript (section 5.2).

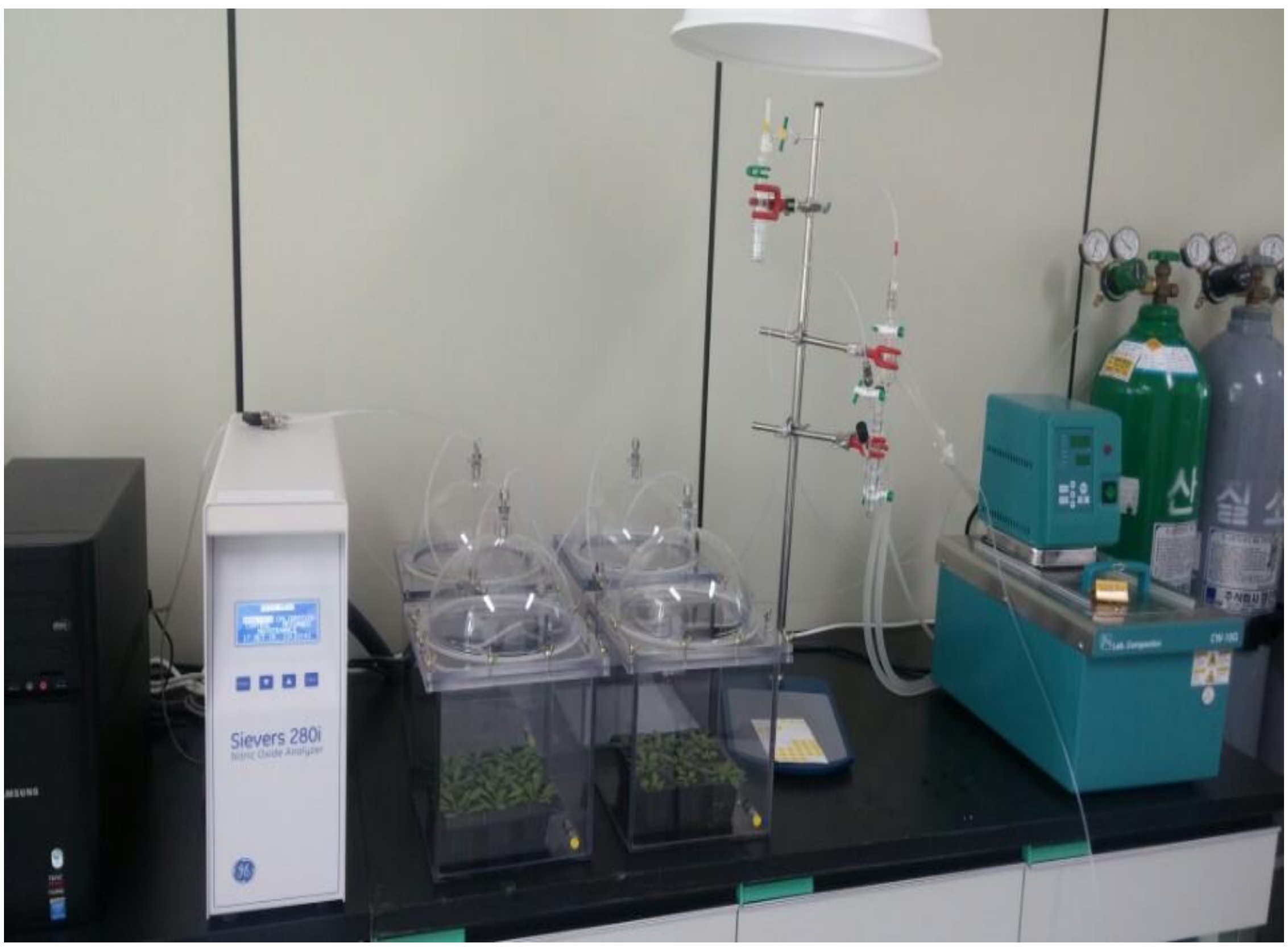

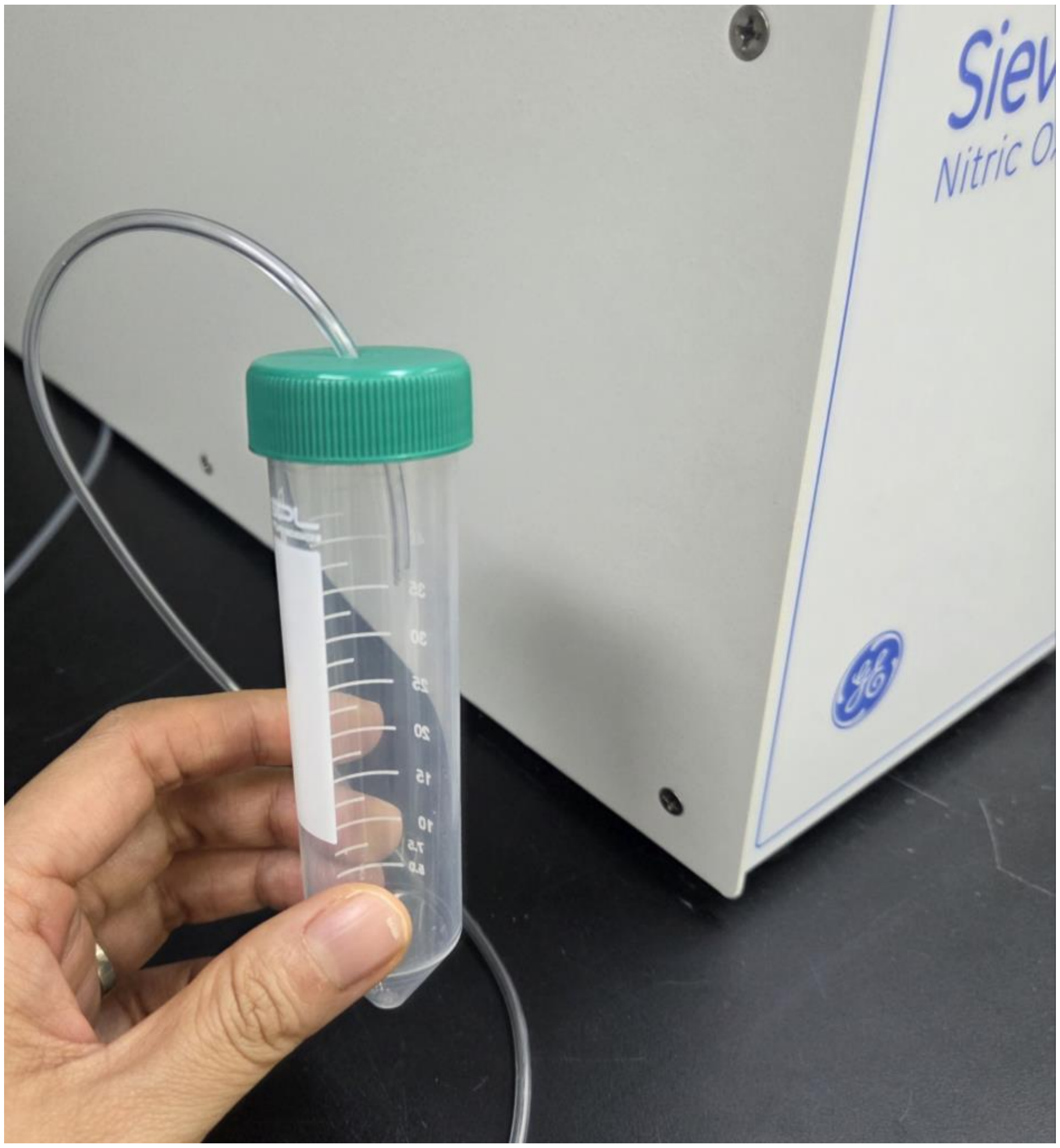

.
So, to address the reviewer's comment bullet-wise;

- NO detection was initiated immediately after the addition of samples to the chamber. As shown in figure 2G, measurements started at “zero” or instantly after adding the samples.
- As described above, when the equipment is being used in “Aqueous mode” samples are added to a “strong reducing buffer" (e.g., Cu, ascorbate or iodide). However, we did not use a strong reducing buffer. Instead, the measurements were performed in a simple, mildly acidic aqueous solution. This condition was specifically chosen to test the intrinsic properties of our nanocomposite. As stated in our manuscript, *"Given the critical role of ferric ions in GSNO immobilization and the presence of electron-donating groups in humic acids, we hypothesized that HAs may also mediate GSNO reduction, thereby contributing to NO release."* To evaluate this hypothesis without reduction by strong reducing buffers, we analyzed our samples for NO release kinetics under mild acid conditions, inside a sealed chamber.
- The GAS NO measurements were conducted under dark conditions as GSNO is also known to release NO due to photo-reduction.

The revised manuscript has now been modified, and the following text has been added to the materials and methods section 5.2.

To evaluate the NO release kinetics on a strictly 1:1 equivalent basis, the amount of pure GSNO used as a control was quantitatively determined based on Supplementary Table S3. Since 1 g of the GSNO-HAs-Fe-HAP nanocomposite contains 14.2 mg of GSNO, exactly 14.2 mg of pure GSNO powder was used as a control. Hence, 1 g of the nanocomposite and the equivalent 14.2 mg of pure GSNO powder were added to 1 mL of 1 M citric acid in a sealed chamber to measure the NO gas released from GSNO and the NP composite under dark conditions. *For measurements of S-nitrosothiols, the detailed methodology is given in Supplementary Text S11.*

Response to review comments

We thank the editor(s) and the reviewers for their time, effort, and valuable critique of our manuscript. Their comments have certainly improved the manuscript. Reviewer 1 has already accepted the manuscript for publication. The second reviewer's comments are very insightful and encouraging. We have provided a point-to-point response to their comments below and further improved the manuscript. All the changes in the manuscript have been highlighted in green. We hope that the editors/reviewers will find these changes satisfactory.

Reviewer 2

1. I have one remaining comment concerning the NO-release experiment presented in Figure 2G. As a researcher who routinely works with various nitric oxide detection and quantification techniques, I found the additional methodological information helpful. However, the mechanistic interpretation of the NO-release kinetics remains unclear. The authors state that the measurements were performed in a sealed chamber, under dark conditions, and without the use of a strong reducing buffer, using 1 M citric acid as the reaction medium. Under these conditions, it is not immediately evident which physicochemical processes are responsible for the temporally variable NO-release profile observed over several days daily fluctuations in case of GSNO-HAs-Fe-HAP). While the manuscript proposes a potential role for humic acids and Fe-mediated interactions in GSNO transformation and NO release, this explanation remains largely speculative. Therefore, I encourage the authors to expand the discussion of Figure 2G and provide a more detailed explanation of the mechanisms that may underlie the observed NO-release dynamics. Such clarification would strengthen one of the central findings of the study and improve the interpretation of the NO-release data.

Response: Thank you for your constructive comments. First, we would like to clarify that the NP composite was not treated with a solution containing 1 M citric acid. Rather, a 1 M citric acid stock solution was used only to adjust the pH of the aqueous suspension containing the NP composite to pH 4.5. This acidic condition was selected to promote particle dissolution and, to some extent, to mimic the acidic microenvironment that may develop in the rhizosphere owing to the secretion of organic acids from plant roots. Dissolution of the NP composite subsequently resulted in the gradual release of the incorporated GSNO, Fe, and humic acids.

We propose two possible explanations for the more sustained NO release observed in the presence of Fe and humic acids. First, the prolonged release may be attributed to the gradual liberation of structurally stabilized GSNO from the surface or matrix of the NP composite. GSNO may remain relatively stable while physically associated with humic acids and coordinated with Fe ions within the particles. However, once it is

released during particle dissolution, GSNO may undergo decomposition or transformation, thereby generating NO.

Second, the reducing capacity of the co-released humic acids may promote the transformation of GSNO. The humic acids used in this study contained phenolic functional groups, as experimentally confirmed in another research. These phenolic moieties can be oxidized to quinone-like structures, and phenol–quinone redox cycling in humic substances is widely recognized as an important mediator of environmental redox reactions. Accordingly, reductive reactions mediated by the co-released humic acids may facilitate GSNO decomposition and contribute to sustained NO generation. In fact, it has been reported that redox reactions can facilitate NO release from GSNO (See the reference below).

Decomposition of S-nitrosoglutathione in the presence of copper ions and glutathione. Arch. Biochem. Biophys. 1996 Jun 15;330(2):219-28.

Based on these considerations, we have revised the manuscript by incorporating the above information in the discussion section.

2. The presentation of the Cd tolerance index in Figure 4A,B is somewhat confusing. Since the tolerance index is intended to assess plant performance under cadmium stress relative to unstressed controls, it appears that all treatments included in these panels were exposed to Cd. If this is the case, the labels “Control”, “NPK”, and “GSNO-HAs-Fe-HAP” may be misleading and should be revised to indicate Cd-treated groups as in other parameters.

Response: Apologies for the confusion. Yes, all the treatments were exposed to Cd. We have now updated the figure 4 legend labels to include Cd.
